# Adverse Events Following Short-Course Systemic Corticosteroids Among Children and Adolescents: A Systematic Review and Meta-Analysis

**DOI:** 10.1001/jamanetworkopen.2025.34953

**Published:** 2025-09-30

**Authors:** João Pedro Lima, Saifur R. Chowdhury, Wimonchat Tangamornsuksan, Chunjuan Zhai, Xiajing Chu, Jessyca Matos Silva, Humayun Kabir, Mahmudur Rahman Chowdhury, Rachel Couban, Michael Walsh, Bram Rochwerg, Mohamed Eltorki, Gordon H. Guyatt, Derek Chu

**Affiliations:** 1 Department of Health Research Methods, Evidence, and Impact, McMaster University, Hamilton, Ontario, Canada; 2 Department of Anesthesia, McMaster University, Hamilton, Ontario, Canada; 3 Michael G. DeGroote Centre for Transfusion Research, McMaster University, Hamilton, Ontario, Canada; 4 Department of Public Health, North South University, Dhaka, Bangladesh; 5 Department of Pediatrics, McMaster University, Hamilton, Ontario, Canada; 6 Department of Pediatrics, Cumming School of Medicine, University of Calgary, Alberta, Canada; 7 Department of Medicine, McMaster University, Hamilton, Ontario, Canada; 8 MAGIC Evidence Ecosystem Foundation, Oslo, Norway; 9 Department of Cardiology, Shandong Provincial Hospital, Shandong First Medical University, Jinan, Shandong, China; 10 Population Health Research Institute, Hamilton Health Sciences, McMaster University, Hamilton, Ontario, Canada

## Abstract

**Importance:**

Short courses of systemic corticosteroids are used in the management of a number of acute clinical conditions, including Bell palsy, croup, and pneumonia, but research on associations of corticosteroid use with adverse events (AEs) in children is limited.

**Objective:**

To document AEs associated with short-term (≤14 days) use of systemic corticosteroids in children and adolescents (1 to younger than 18 years) across different clinical conditions.

**Data Sources:**

MEDLINE, Embase, and the Cochrane Central Register of Controlled Trials (CENTRAL) databases were searched from inception to February 2025. Reference lists of eligible articles and related systematic reviews were also searched.

**Study Selection:**

Randomized clinical trials evaluating AEs (any unfavorable and unintended signs, symptoms, or syndromes that occurred during the period of using an investigational product) after use of short-course systemic corticosteroids in children and adolescents were included.

**Data Extraction and Synthesis:**

Using the Preferred Reporting Items for Systematic Reviews and Meta-Analyses reporting guideline, pairs of reviewers independently reviewed abstracts, extracted data, and assessed risk of bias of eligible trials. Pairwise, fixed-effects meta-analyses were performed using Mantel-Haenszel methods with risk difference (RD).

**Main Outcomes and Measures:**

The primary outcomes were serious AEs (events resulting in death, life-threatening conditions, hospitalization, or substantial disability), AEs leading to discontinuation, hyperglycemia, sleep disturbances, change in behavior, and gastrointestinal bleeding. RDs were reported as AEs per 1000 patients with 95% CIs. The Grading of Recommendations Assessment, Development and Evaluation approach was used to assess the certainty of evidence (high, moderate, low, or very low certainty).

**Results:**

A total of 45 eligible trials that included 6470 children (mean [SD] age, 5.57 [3.62] years; 3753 male [58%]) were identified. In pooled analysis, there was moderate certainty evidence that compared with usual care, corticosteroids were not associated with serious AEs (RD, 1 fewer AE per 1000 patients [95% CI], 9 fewer to 7 more AEs per 1000 patients]), AEs leading to discontinuation (RD, 4 more AEs per 1000 patients [95% CI, 3 fewer to 11 more AEs per 1000 patients]), or change in behavior (RD, 8 more AEs per 1000 patients [95% CI, 5 fewer to 21 more AEs per 1000 patients]). With moderate certainty evidence, corticosteroids were associated with an increased risk of hyperglycemia (RD, 38 more AEs per 1000 patients [95% CI, 11 to 64 more AEs per 1000 patients]) and sleep problems (RD, 15 more AEs per 1000 patients [95% CI, 1 to 28 more AEs per 1000 patients]). Corticosteroid use was also associated with an increased risk of gastrointestinal bleeding (RD, 13 more per AEs per 1000 patients [95% CI, 3 to 23 more AEs per 1000 patients]), but the certainty of evidence was low.

**Conclusions and Relevance:**

In this systematic review and meta-analysis of randomized trials, there was moderate certainty evidence that corticosteroids were associated with an increased risk of hyperglycemia and sleep problems and low certainty evidence that corticosteroids were associated with increased risk of gastrointestinal bleeding, but these AEs were very seldom serious. These findings suggest that an individualized approach to short-term corticosteroid use may be warranted and that further research is needed to obtain better quality of evidence.

## Key Points

**Question:**

What adverse events are associated with the use of systemic corticosteroids for 14 days or less in children and adolescents?

**Findings:**

This systematic review and meta-analysis including 45 eligible trials involving 6470 children and adolescents found with moderate quality of evidence that corticosteroids were associated with an increased risk of hyperglycemia and sleep disturbance. Corticosteroids were also associated with increased risk of gastrointestinal bleeding, but the quality of evidence was low.

**Meaning:**

These findings suggest that an individualized approach to short-term corticosteroid use in children and adolescents may be warranted and that further research is needed to obtain better quality of evidence.

## Teaser

This systematic review and meta-analysis investigates whether short-term use of systemic corticosteroids is associated with adverse events among children and adolescents.

## Introduction

Corticosteroids are immunosuppressant anti-inflammatory drugs that, over the decades, have been widely used to treat a variety of children’s illnesses including asthma, croup, and urticaria.^1,2,3,4^ From 2012 to 2017, corticosteroid prescriptions in Canada for children between 3 and 19 years totaled 55 000, with a prevalence of 0.9%.^5^ Systematic reviews have investigated the harms of short-term use of corticosteroids in children; one study^6^ synthesized safety outcomes of corticosteroids but restricted to specific medical conditions, such as acute respiratory conditions, while another study^7^ restricted to only oral routes of corticosteroids.

In adults, the treatment of corticosteroid-associated adverse events (AEs) represents a substantial financial burden to patients and health care systems.^8,9^ This burden is expected to be comparable in pediatric populations. To our knowledge, there is no systematic review and meta-analysis available examining the harms of short-course systemic corticosteroids in a pediatric population across different illnesses. AEs are frequently misreported and underrepresented in systematic reviews when compared with effective outcomes.^10,11^ Observational studies are limited by uncertainty of whether apparent AEs are actually symptoms arising from the illness or are related to the drug themselves.^12^

A major limitation of prior individual studies, and even systematic reviews of studies of individual clinical conditions, is relatively small numbers of patients, resulting in imprecision of estimates and low certainty evidence. While effectiveness of corticosteroids is likely to differ across clinical conditions, adverse effects are likely to be very similar. Therefore, we undertook a systematic review and meta-analysis of randomized clinical trials (RCTs) to investigate the harms of short-term use of systemic corticosteroids in patients between 1 and younger than 18 years that can be generalized across a variety of pediatric clinical conditions. We limited our population to those older than 1 year because of the rapid physiological and behavioral changes that occur before 1 year but not after that age.

## Methods

This systematic review and meta-analysis adhered to the 2020 Preferred Reporting Items for Systematic Reviews and Meta-Analyses (PRISMA) reporting guideline^13^ and PRISMA harms checklist.^14^ We registered this systematic review in PROSPERO (CRD42023400934).

### Literature Search

With the help of a health science librarian, we searched MEDLINE, Embase, and the Cochrane Central Register of Controlled Trials (CENTRAL) databases from inception to February 2025 using a combination of keywords and medical subject headings terms related to systemic corticosteroids and AEs (eMethods in Supplement 1). We also searched for additional eligible studies from the reference lists of eligible articles and related systematic reviews.^1,6,7,15^ We did not use any language restrictions.

### Eligibility Criteria

We included published RCTs evaluating the harms of short-course systemic (oral, sublingual, rectal, intravenous, intramuscular, and subcutaneous) corticosteroids in any condition. Eligible RCTs included noncritically ill children aged 1 to younger than 18 years and reported on AEs during short-course use of systemic corticosteroids—dexamethasone, prednisolone, prednisone, and methylprednisolone plus prednisone and hydrocortisone at any dose—vs placebo or nonsteroidal standard of care. We defined AEs as any unfavorable and unintended signs, symptoms, or syndromes that occur during the period of using an investigational product, regardless of their perceived relationship to the product^16^ and short-course as the use of the drug for no more than 14 days at least once daily. Given the inconsistency in the terminology used to refer to AEs in RCTs, we considered all studies that reported on *adverse events*, *adverse drug reactions*, *side effects*, *harms*, *safety*, or *toxicity* of corticosteroids. Serious AEs (SAEs) were defined as events resulting in death, life-threatening conditions, hospitalization, or substantial disability, while AEs leading to discontinuation were events that caused cessation of corticosteroid treatment due to their severity or persistence. We included studies with a population of at least 80% of children within the age criteria. We excluded trials involving inhaled interventions, perioperative corticosteroids for surgical patients, immunosuppressed patients, and patients with HIV or AIDS and cancer.

### Study Selection

Using Covidence systematic review software (Veritas Health Innovation), pairs of reviewers (S.R.C., W.T., C.Z., X.C., J.M.S., H.K., and M.R.C.) worked independently and in duplicate to screen titles and abstracts and, subsequently, full texts of the potentially eligible trials. Reviewers resolved disagreements by consensus or, if necessary, by consultation with a third reviewer (J.P.L.).

### Data Extraction

Pairs of reviewers (S.R.C., W.T., C.Z., X.C., J.M.S., H.K., and M.R.C.) independently extracted data from the eligible trials using a standardized data extraction form and resolved disagreements by discussion or, when necessary, through adjudication by a third reviewer (J.P.L.). Reviewers collected information describing trial characteristics (publication year, trial registration, designs, and country), patient characteristics (age, sex, comorbidities, setting, and conditions for which corticosteroids were used), intervention descriptions (corticosteroid molecule, route of administration, and doses), and extracted data on all AEs reported in the trials from the intention-to-treat population, and if not reported, in preferred order: modified intention-to-treat, per-protocol, and as-treated population. If authors reported data at different time points, we extracted data from the latest time point at which the intervention was still in use.

### Risk of Bias Assessment

Pairs of reviewers (S.R.C., W.T., C.Z., X.C., J.M.S., H.K., and M.R.C.) independently performed the risk of bias assessment using the modified Cochrane tool for assessing the risk of bias in randomized trials, and resolved any disagreements, when necessary, by consensus or consultation with another reviewer (J.P.L.). We considered bias arising in the randomization process; bias due to inadequate allocation concealment; bias owing to unblinding of patients, health care practitioners, and outcome assessors; and bias from missing outcome data. We rated each domain as either low risk of bias, probably low risk of bias, probably high risk of bias, or high risk of bias.

### Statistical Analysis

We performed all analyses using the meta and metafor packages in R version 4.03 (R Foundation for Statistical Computing). A 2-sided P < .05 was considered significant.

#### Primary Analysis

We conducted pairwise meta-analysis for all AEs, in which at least 2 studies reported at least 1 event (eFigure 1 in Supplement 1). Based on the framework proposed by Xu et al^17^ for meta-analysis containing 0 events occurring in both single and double groups, we performed a fixed-effects meta-analysis of risk difference (RD) using the Mantel-Haenszel methods without continuity correction. We summarized the outcomes of interventions using RDs (AEs per 1000 patients with associated 95% CIs). For outcomes with 10 or more studies, we assessed for small study effect sizes using visual inspection of funnel plots (eFigure 2 in Supplement 1).

#### Subgroup and Sensitivity Analyses

When there were at least 2 studies per subgroup reporting at least 1 event, we performed a priori subgroup analyses based on medication route (oral vs other routes), dose (high-dose vs low-dose; high-dose defined as ≥1 mg/kg/d of prednisone), treatment duration (≤7 days vs >7 to 14 days), and clinical condition. We hypothesized that intravenous or intramuscular, more than 7 days’ use, and higher doses of corticosteroids would be associated with a higher risk of AEs. To determine the glucocorticoid outcome, we used the following corticosteroid conversions: 1 mg of dexamethasone = 26.7 mg of hydrocortisone = 5.3 mg of methylprednisolone or prednisolone = 6.7 mg of prednisone.^18,19^ We assessed the credibility of statistically significant subgroups using the Instrument for Assessing the Credibility of Effect Modification Analyses (ICEMAN).^20^ To test the robustness of our analyses and assess whether corticosteroid risk was invariant of baseline risks, we performed a sensitivity analysis using the Peto odds ratio (OR).

#### Certainty of Evidence Assessment

The Grading of Recommendations Assessment, Development and Evaluation (GRADE) approach guided the certainty of evidence assessment for each outcome. The outcomes were rated as high, moderate, low, or very low certainty of evidence. Eligible trials started as high certainty of evidence and were rated down for risk of bias, inconsistency, imprecision, indirectness, or publication bias. Based on a recent GRADE guidance,^21,22^ we rated imprecision using the null effect (RD = 0) as the threshold of interest. Pairs of reviewers (J.P.L. and S.R.C.) assessed the certainty of evidence independently and, when necessary, resolved disagreements through consensus or discussion with another reviewer (G.H.G. and D.C.).

We developed a summary of findings table using the MAGICapp. In the table, AEs were grouped together based on the system organ classes (eg, gastrointestinal disorders) from the Medical Dictionary for Regulatory Activities.^23^

## Results

**Figure 1.**
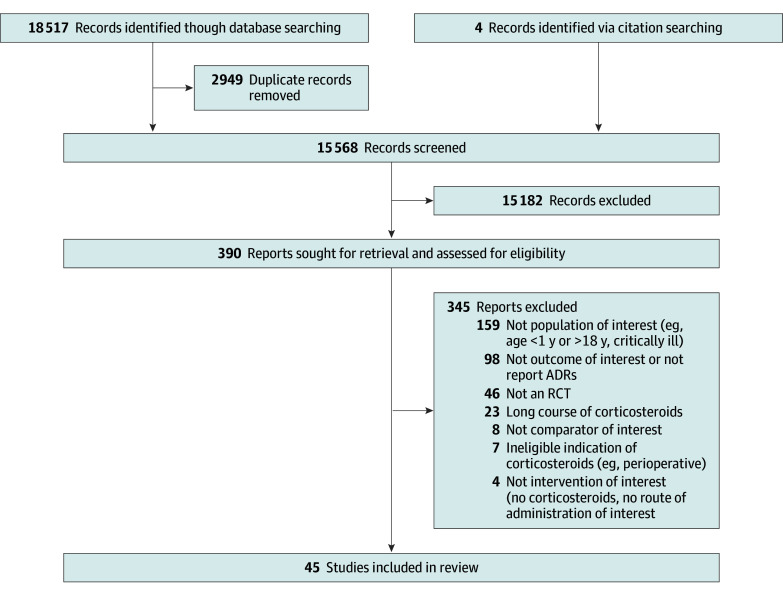
Flow Diagram of Selected Studies ADR indicates adverse drug reaction; RCT, randomized clinical trial.

Our search identified 18 517 articles from database searching and 4 records from citation searching. After removing duplicates, we screened titles and abstracts of 15 572 articles and excluded 15 182 articles. The main reasons for exclusion at this stage were the absence of randomization procedures, inclusion of adult populations, and corticosteroid use exceeding 14 days. After full text review, 45 RCTs proved eligible.^24,25,26,27,28,29,30,31,32,33,34,35,36,37,38,39,40,41,42,43,44,45,46,47,48,49,50,51,52,53,54,55,56,57,58,59,60,61,62,63,64,65,66,67,68^ Figure 1 summarizes the study selection process.

### Trial and Participants Characteristics

Forty-five studies included 6470 children with a mean (SD) age of 5.57 (3.62) years, and 3753 (58%) were male. The majority were conducted in Europe and North America (31 studies^25,26,27,28,29,30,32,33,37,38,39,40,41,42,43,44,45,47,48,49,51,52,53,57,58,59,61,62,63,66,67^ [69%]), followed by Asia (12 studies^34,35,36,46,50,54,55,56,60,64,65,68^ [27%]) and Australia and New Zealand (2 studies^24,31^ [4%]). The most common indications for corticosteroids included were encephalitis or meningitis (6 trials^36,40,41,44,55,58^ [13%]), croup (5 trials^26,39,42,45,62^ [11%]), and pneumonia (5 trials^46,48,60,63,68^ [11%]).

Of all trials, 17 (including 2421 participants) investigated dexamethasone,^25,26,34,35,36,39,40,41,42,44,45,51,55,58,59,62,63^ 13 trials (964 participants) investigated methylprednisolone,^27,33,46,48,49,50,54,60,61,65,66,67,68^ 12 trials (2313 participants) investigated prednisolone,^24,28,29,30,31,32,38,43,47,56,57,64^ 2 trials (727 participants) examined prednisone,^37,52^ and 1 study (45 participants) investigated hydrocortisone, dexamethasone, or betamethasone.^53^ The median [IQR] duration of corticosteroid treatment was 3 [1-5] days. In 27 trials^25,27,33,34,35,36,39,40,41,42,44,45,46,48,49,50,53,54,55,58,60,61,62,63,65,67,68^ (60%), corticosteroid administration was intravenous or intramuscular, and in 18 trials^24,26,28,30,31,32,37,38,39,43,47,51,52,56,57,59,64,66^ (40%) corticosteroids were administered orally. While 32 trials^25,27,28,31,33,34,35,36,38,40,41,42,43,44,45,46,48,49,50,52,53,54,55,58,60,61,62,63,64,65,67,68^ (71%) were conducted in an inpatient setting, 11 studies^24,26,30,32,37,39,51,56,57,59,66^ (24%) were outpatient only, and 2 studies^29,47^(4%) included a mixed population. Table 1 presents the characteristics of all included studies.

**Table 1.** Study Characteristics

Source	Trial registration	Funding	Country	Age, mean, y	Participants randomized, No.	Male, No. (%)	Inpatient, No. (%)	Clinical condition	Intervention
Griffin et al,^33^ 1994	NR	Institutional	US	7.70	56	33 (59)	56 (100)	Sickle cell disease	Methylprednisolone: 2 doses of 15 mg/kg IV for 2 d
Hoffman et al,^35^ 1988	NR	None	Indonesia	10.20	38	23 (61)	38 (100)	Cerebral malaria	Dexamethasone: initial dose of 3 mg/kg, followed by 6 doses of 1.4 mg/kg 4 times daily IV for 2 d
Hoke et al,^36^ 1992	NR	Government	Thailand	8.73	55	39 (71)	55 (100)	Japanese encephalitis	Dexamethasone: 0.6 mg/kg as a loading dose followed by 0.2 mg/kg 4 times daily IV for 5 d
Johnson et al,^39^ 1998	NR	Industry	Canada	2.00	96	67 (69)	0	Acute laryngotracheobronchitis (croup)	Dexamethasone: 0.6 mg/kg IM once
Kanra et al,^40^ 1995	NR	NR	Türkiye	7.10	53	32 (60)	53 (100)	Pneumococcal meningitis	Dexamethasone: 0.6 mg/kg/d divided into 4 daily doses IV for 4 d
King et al,^41^ 1994	NR	Not-for-profit foundation	Canada	1.00	101	45 (45)	101 (100)	Suspected bacterial meningitis	Dexamethasone: 0.6 mg/kg/d every 6 h IV for 4 d
Babl et al,^24^ 2022	ANZCTR: ACTRN12615 000563561	Industry, Government	Australia and New Zealand	10.37	187	90 (48)	0	Bell palsy	Prednisolone: 1 mg/kg/d oral for 10 d
Lebel et al,^44^ 1988	NR	Institutional	US	1.29	200	105 (53)	200 (100)	Bacterial meningitis	Dexamethasone: 0.15 mg/kg every 6 h IV for 4 d
Lv et al,^46^ 2022	NR	NR	China	5.64	120	73 (61)	120 (100)	Refractory mycoplasma pneumonia	Methylprednisolone: 2 mg/kg/d IV for 7 d
Medeiros et al,^47^ 2007	NR	Government	US	10.00	45	5 (11)	13 (29)	Acute hemarthrosis	Prednisolone: 2mg/kg/d divided into 3 daily doses oral for 2 d
Newburger et al,^49^ 2007	ClinicalTrials.gov: NCT00132080	Government	Canada and US	2.90	198	122 (62)	198 (100)	Kawasaki disease	Methylprednisolone: 30 mg/kg IV once
Ogata et al,^50^ 2012	UMIND: UMIN000005021	Government	Japan	3.45	48	24 (50)	48 (100)	Kawasaki disease	Methylprednisolone: 30 mg/kg IV once
Panickar et al,^52^ 2009	UK Clinical Study Registry: ISRCTN58363576	Not-for-profit foundation	UK	2.17	687	443 (64)	687 (100)	Acute virus-induced wheezing	Prednisone: 10 mg once a day for children ≤24 mos and 20 mg once a day for children >24 mos oral for 5 d
Prakash et al,^54^ 2006	NR	NR	India	15.81	52	36 (69)	52 (100)	Solitary cysticercus granuloma with new-onset seizures	Methylprednisolone: 1.0 g/1.72 m^2^/d IV for 5 d
Qazi et al,^55^ 1996	NR	Institutional	Pakistan	3.16	89	54 (61)	89 (100)	Suspected bacterial meningitis	Dexamethasone: 0.6 mg/kg/d in 4 divided doses IV for 4 d
Ranakusuma et al,^56^ 2020	ANZCTR: ACTRN12618 000049279	Government	Indonesia	5.58	62	33 (53)	0	Acute otitis media	Prednisolone: 10 mg/d for children aged 6 mos to 2 y, 20 mg/d for children aged 2-6 y; and 30 mg/d for children aged 6-12 y oral for 5 d
Ruohola et al,^57^ 1999	NR	Institutional	Finland	1.91	50	28 (56)	0	Acute otitis media	Prednisolone: 2 mg/kg/d divided in 3 equal doses oral for 3 d
Schaad et al,^58^ 1993	NR	Industry	Switzerland	3.00	115	69 (60)	115 (100)	Bacterial meningitis	Dexamethasone: 0.4 mg/kg every 12 h IV for 2 d
Shaikh et al,^59^ 2020	ClinicalTrials.gov: NCT01391793	Government	US	1.58	546	44 (8)	0	Urinary tract infection	Dexamethasone: 0.15 mg/kg twice daily oral for 3 d
Shan et al,^60^ 2017	NR	Government	China	7.34	112	57 (51)	112 (100)	Refractory mycoplasma pneumonia	Methylprednisolone: 2 mg/kg/d IV for 3 d
Tagarro et al,^63^ 2017	ClinicalTrials.gov: NCT01261546	Government	Spain	4.70	60	25 (42)	60 (100)	Parapneumonic pleural effusion	Dexamethasone: 0.25 mg/kg every 6 h IV for 2 d
Olympia et al,^51^ 2005	NR	NR	US	11.94	125	52 (42)	0	Pharyngitis	Dexamethasone: 0.6 mg/kg oral once
Kuusela et al,^42^ 1988	NR	NR	Finland	2.85	72	56 (78)	72 (100)	Acute laryngotracheobronchitis (croup)	Dexamethasone: 0.6 mg/kg IM once
Bernini et al,^25^ 1998	NR	Not-for-profit foundation	US	6.21	43	29 (67)	43 (100)	Sickle cell disease	Dexamethasone: 0.3 mg/kg every 12 h IV for 2 d
Bjornson et al,^26^ 2004	NR	Institutional, not-for-profit	Canada	2.92	720	439 (61)	0	Acute laryngotracheobronchitis (croup)	Dexamethasone: 0.6 mg/kg oral once
Carcao et al,^27^ 2016	ClinicalTrials.gov: NCT00376077	Not-for-profit foundation	Canada	9.40	32	22 (69)	32 (100)	Immune thrombocytopenia	Methylprednisolone: 30 mg/kg IV once
Connett et al,^28^ 1994	NR	NR	US	4.85	70	49 (70)	70 (100)	Acute asthma	Prednisolone: 2 mg/kg oral once
Dudley et al,^30^ 2013	UK Clinical Study Registry: ISRCTN71445600	Government	UK	6.00	352	194 (55)	0	Henoch-Schönlein purpura	Prednisolone: 2 mg/kg/d for 7 d followed by 1 mg/kg/d for 7 d oral
Foster et al,^31^ 2018	ANZCTR: ACTRN12612 000394842	Government	Australia	3.44	624	420 (67)	624 (100)	Acute virus-induced wheezing	Prednisolone: 1 mg/kg/d oral for 3 d
Francis et al,^32^ 2018	UK Clinical Study Registry: ISRCTN49798431	Government	UK	5.19	389	216 (56)	0	Otitis media	Prednisolone: 20 mg/d for children aged 2-5 y or 30 mg/d for children aged 6-8 y oral for 7 d
Tam et al,^64^ 2012	UK Clinical Study Registry: ISRCTN39575233	Not-for-profit foundation	Vietnam	13.00	225	161 (72)	225 (100)	Dengue	Prednisolone: 2 mg/kg or 0.5 mg/kg once daily oral for 3 d
Tassniyom et al,^65^ 1993	NR	Institutional, not-for-profit	Thailand	6.61	63	32 (51)	63 (100)	Dengue	Methylprednisolone: 30 mg/kg IV once
Usta et al,^67^ 2014	ClinicalTrials.gov: NCT02002078	None	Türkiye	4.10	83	45 (54)	83 (100)	Caustic esophageal burns	Methylprednisolone: 1 g/1.73 m^2^/d IV for 3 d
Wang et al,^68^ 2022	NR	NR	China	6.65	60	32 (53)	60 (100)	Mycoplasma pneumoniae pneumonia	Methylprednisolone: 2 mg/kg/d IV for 5 d
Pierson et al,^53^ 1974	NR	Not-for-profit foundation	US	12.06	45	28 (62)	45 (100)	Acute asthma	(1) hydrocortisone: 7 mg/kg as an immediate or priming dose, followed by 7 mg/kg/d administered continuously by IV infusion; (2) dexamethasone: 0.3 mg/kg, given immediately followed by 0.3 mg/kg/24 h by continuous IV infusion; (3) betamethasone: 0.3 mg/kg, as a primary dose followed by 0.3 mg/kg/d by continuous IV infusion in 24 h
Huber et al,^37^ 2004	UK Clinical Study Registry: ISRCTN85109383	NR	Canada	5.52	40	20 (50)	0	Henoch-Schönlein purpura	Prednisone: 2 mg/kg for 7 days, followed by a weaning dose of prednisone for the next 7 days oral
Csonka et al,^29^ 2003	NR	Not-for-profit foundation	Finland	1.41	230	150 (65)	123 (53)	Viral respiratory infection–induced lower airway disease	Prednisolone: 2 mg/kg/d in 2 divided daily doses oral for 4 d
Sundel et al,^61^ 2003	NR	Not-for-profit foundation	US	4.39	39	27 (69)	39 (100)	Kawasaki disease	Methylprednisolone: 30 mg/kg IV once
Harel et al,^34^ 2011	NR	NR	Israel	2.75	49	NR	49 (100)	Septic arthritis	Dexamethasone: 0.15 mg/kg every 6 h IV for 4 d
Kvien et al,^43^ 1982	NR	Not-for-profit foundation	Norway	10.54	20	5 (25)	20 (100)	Juvenile rheumatoid arthritis	Prednisolone: 0.4 mg/kg/d oral for 7 d
Jartti et al,^38^ 2007	NR	Industry, government, not-for-profit	Finland	2.60	59	39 (66)	59 (100)	Recurrent wheezing	Prednisolone: 2 mg/kg/d oral for 3 d
Leipzig et al,^45^ 1979	NR	NR	US	1.76	30	NR	30 (100)	Acute laryngotracheobronchitis (croup)	Dexamethasone: 0.3 mg/kg at the time of admission and repeated in 2 h IM
Nagy et al,^48^ 2013	EudraCT: 2007-006602-24	Not-for-profit foundation	Hungary	4.91	59	35 (59)	59 (100)	Community-acquired pneumonia	Methylprednisolone: 0.5-2.0 mg/kg IV for 5 d
Unüvar et al,^66^ 1999	NR	NR	Türkiye	4.74	42	22 (52)	0	Bell palsy	Methylprednisolone: 1 mg/kg/d oral for 14 d
Super et al,^62^ 1989	NR	NR	US	1.30	29	19 (66)	29 (100)	Acute laryngotracheobronchitis (croup)	Dexamethasone: 0.6 mg/kg IV or IM once

Abbreviations: ANZCTR, Australian New Zealand Clinical Trials Registry; EudraCT, European Union Drug Regulating Authorities Clinical Trials Database; IM, intramuscular; IV, intravenous; NR, not reported; UMIN, University Hospital Medical Information Network.

### Risk of Bias Assessment

Of the 45 eligible trials, 15 trials^25,38,39,46,48,50,51,54,56,59,60,61,66,67,68^ (33%) had at least 1 domain at probably high or high risk of bias. Failure to blind was the main reason for rating down risk of bias; there were 11 trials^39,46,48,50,54,56,60,61,66,67,68^(24%) that did not blind participants, 8 trials^46,48,50,54,60,61,66,68^ (18%) that did not blind health care practitioners, and 9 trials^46,48,50,54,60,61,66,67,68^ (20%) that did not blind data collectors. Table 2 presents the detailed risk of bias assessment.

**Table 2.** Risk of Bias Assessment

Source	Risk of bias by domain					
	1: Was the allocation sequence adequately generated?	2: Was the allocation adequately concealed?	3: Were patients blinded?	4: Were health care practitioners blinded?	5: Were data collectors blinded?	6: Was loss to follow-up (missing outcome data) infrequent?
Griffin et al,^33^ 1994	Definitely yes	Probably yes	Probably yes	Definitely yes	Probably yes	Definitely yes
Hoffman et al,^35^ 1988	Definitely yes	Definitely yes	Probably yes	Probably yes	Probably yes	Definitely yes
Hoke et al,^36^ 1992	Definitely yes	Probably yes	Probably yes	Probably yes	Probably yes	Definitely yes
Johnson et al,^39^ 1998	Definitely yes	Definitely yes	Definitely no	Probably yes	Probably yes	Definitely no
Kanra et al,^40^ 1995	Definitely yes	Probably yes	Probably yes	Probably yes	Probably yes	Definitely yes
King et al,^41^ 1994	Definitely yes	Probably yes	Probably yes	Probably yes	Probably yes	Definitely yes
Babl et al,^24^ 2022	Definitely yes	Definitely yes	Definitely yes	Definitely yes	Definitely yes	Probably yes
Lebel et al,^44^ 1988	Definitely yes	Probably yes	Probably yes	Probably yes	Probably yes	Definitely yes
Lv et al,^46^ 2022	Probably yes	Probably no	Probably no	Probably no	Probably no	Definitely yes
Medeiros et al,^47^ 2007	Probably yes	Probably yes	Probably yes	Probably yes	Probably yes	Definitely yes
Newburger et al,^49^ 2007	Probably yes	Probably yes	Probably yes	Probably yes	Probably yes	Definitely yes
Ogata et al,^50^ 2012	Definitely yes	Probably no	Definitely no	Definitely no	Definitely no	Definitely yes
Panickar et al,^52^ 2009	Definitely yes	Definitely yes	Definitely yes	Definitely yes	Definitely yes	Definitely yes
Prakash et al,^54^ 2006	Definitely yes	Probably no	Definitely no	Definitely no	Definitely no	Definitely yes
Qazi et al,^55^ 1996	Definitely yes	Definitely yes	Definitely yes	Definitely yes	Probably yes	Definitely yes
Ranakusuma et al,^56^ 2020	Definitely yes	Definitely yes	Definitely no	Definitely yes	Definitely yes	Definitely yes
Ruohola et al,^57^ 1999	Definitely yes	Probably yes	Probably yes	Probably yes	Probably yes	Definitely yes
Schaad et al,^58^ 1993	Definitely yes	Probably yes	Definitely yes	Definitely yes	Definitely yes	Definitely yes
Shaikh et al,^59^ 2020	Definitely yes	Definitely yes	Definitely yes	Definitely yes	Definitely yes	Definitely no
Shan et al,^60^ 2017	Definitely yes	Probably yes	Definitely no	Definitely no	Definitely no	Probably yes
Tagarro et al,^63^ 2017	Definitely yes	Definitely yes	Definitely yes	Definitely yes	Definitely yes	Definitely yes
Olympia et al,^51^ 2005	Definitely yes	Definitely yes	Probably yes	Probably yes	Probably yes	Definitely no
Kuusela et al,^42^ 1988	Probably yes	Probably yes	Probably yes	Probably yes	Probably yes	Definitely yes
Bernini et al,^25^ 1998	Definitely yes	Definitely yes	Definitely yes	Definitely yes	Definitely yes	Definitely no
Bjornson et al,^26^ 2004	Definitely yes	Definitely yes	Definitely yes	Probably yes	Probably yes	Definitely yes
Carcao et al,^27^ 2016	Definitely yes	Definitely yes	Definitely yes	Definitely yes	Definitely yes	Definitely yes
Connett et al,^28^ 1994	Definitely yes	Definitely yes	Probably yes	Probably yes	Probably yes	Definitely yes
Dudley et al,^30^ 2013	Definitely yes	Definitely yes	Definitely yes	Definitely yes	Definitely yes	Definitely yes
Foster et al,^31^ 2018	Definitely yes	Definitely yes	Definitely yes	Definitely yes	Definitely yes	Definitely yes
Francis et al,^32^ 2018	Definitely yes	Definitely yes	Definitely yes	Definitely yes	Definitely yes	Definitely yes
Tam et al,^64^ 2012	Definitely yes	Definitely yes	Definitely yes	Definitely yes	Definitely yes	Definitely yes
Tassniyom et al,^65^ 1993	Definitely yes	Probably yes	Definitely yes	Definitely yes	Definitely yes	Definitely yes
Usta et al,^67^ 2014	Probably yes	Probably no	Probably no	Definitely yes	Probably no	Definitely yes
Wang et al,^68^ 2022	Probably yes	Probably no	Probably no	Probably no	Probably no	Definitely yes
Pierson et al,^53^ 1974	Probably yes	Definitely yes	Probably yes	Probably yes	Probably yes	Definitely yes
Huber et al,^37^ 2004	Definitely yes	Definitely yes	Definitely yes	Definitely yes	Definitely yes	Definitely yes
Csonka et al,^29^ 2003	Definitely yes	Definitely yes	Definitely yes	Definitely yes	Definitely yes	Definitely yes
Sundel et al,^61^ 2003	Probably yes	Probably no	Definitely no	Definitely no	Definitely no	Definitely yes
Harel et al,^34^ 2011	Definitely yes	Definitely yes	Definitely yes	Definitely yes	Definitely yes	Definitely yes
Kvien et al,^43^ 1982	Probably yes	Probably yes	Definitely yes	Definitely yes	Definitely yes	Definitely yes
Jartti et al,^38^ 2007	Probably no	Probably yes	Definitely yes	Definitely yes	Definitely yes	Definitely yes
Leipzig et al,^45^ 1979	Probably yes	Probably yes	Definitely yes	Definitely yes	Definitely yes	Definitely yes
Nagy et al,^48^ 2013	Definitely yes	Probably yes	Definitely no	Definitely no	Definitely no	Definitely yes
Unüvar et al,^66^ 1999	Definitely yes	Probably yes	Probably no	Probably no	Probably no	Definitely yes
Super et al,^62^ 1989	Definitely yes	Definitely yes	Definitely yes	Definitely yes	Definitely yes	Definitely yes

### AEs

**Figure 2.**
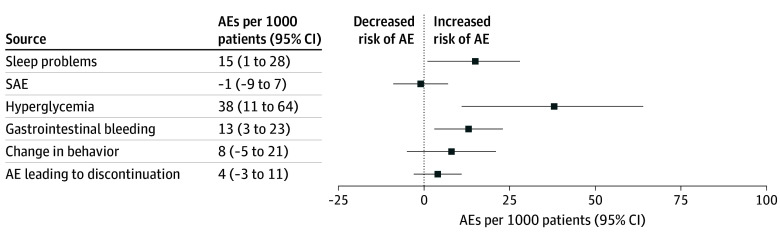
Summary of Risk Differences Across Outcomes AE indicates adverse event; SAE, serious adverse event.

Of the 36 AEs that we were able to meta-analyze, 31 did not show any compelling difference between corticosteroids and the control group. Table 3 and presents a summary of findings with all the outcomes meta-analyzed. eTable 1 in Supplement 1 describes AEs reported in a single trial. Below, we present outcomes that we deemed the most important short-term harms, based on (1) their importance to patients and/or (2) identification of high or moderate certainty evidence of the association of corticosteroid use with the risk of these AEs (Figure 2).

**Table 3.** Summary of Findings

Outcome	Participants, studies, and events, No.	Risk difference, adverse events per 1000 patients (95% CI)	Certainty of the evidence (quality)
Serious adverse events	3418 participants in 19 studies^24,26,27,31,32,34,38,43,45,48,49,50,56,59,60,62,63,64,66^; 52 events	1 (−9 to 7)	Moderate^a^
Adverse events leading to discontinuation	2892 participants in 16 studies^29,31,33,34,37,38,43,45,48,52,58,59,60,62,64,66^; 28 events	4 (−3 to 11)	Moderate^a^
Abdominal pain	2410 participants in 15 studies^24,26,30,37,34,38,43,45,48,51,54,59,60,62,66^; 25 events	5 (−3 to 13)	Moderate^a^
Diarrhea	3280 participants in 19 studies^24,26,29,30,32,34,38,43,45,46,48,56,57,59,60,62,64,66,68^; 80 events	−1 (−12 to 9)	Moderate^a^
Gastritis	488 participants in 10 studies^34,38,43,45,47,48,56,60,62,66^; 7 events	−4 (−25 to 16)	Low^b^
Gastrointestinal bleeding	2555 participants in 22 studies^25,26,30,33,34,35,36,38,39,40,41,43,44,45,48,55,58,60,62,63,64,66^; 49 events	13 (3 to 23)	Low^c^^,^^d^
Hemoccult-positive stool	774 participants in 11 studies^34,38,41,43,44,45,48,58,60,62,66^;69 events	22 (−10 to 54)	Very low^b^^,^^d^
Intussusception	771 participants in 10 studies^30,34,37,38,43,45,48,60,62,66^; 3 events	−8 (−17 to 1)	Low^b^
Nausea	1486 participants in 14 studies^24,27,30,32,34,38,43,45,48,51,56,60,62,66^; 31 events	−8 (−22 to 5)	Moderate^a^
Vomiting	2857 participants in 19 studies^24,28,29,30,34,38,43,45,46,48,51,52,56,59,60,61,62,66,68^; 56 events	−4 (−14 to 6)	Low^a^^,^^e^
Hyperglycemia^f^	792 participants in 12 studies^25,34,38,43,45,48,60,62,63,64,66,67^; 38 events	38 (11-64)	Moderate^f^
Decreased appetite	977 participants in 11 studies^24,32,34,38,43,45,48,60,62,66,68^; 9 events	−10 (−22 to 1)	Low^b^
Increased appetite	1024 participants in 12 studies^24,32,34,38,43,45,47,48,56,60,62,66^; 52 events; 52 events	15 (−6 to 37)	Moderate^a^
Glycosuria^g^	778 participants in 10 studies ^30,34,36,38,43,45,48,60,62,66^; 19 events	36 (16 to 55)	Low^a^^,^^f^
Polyuria	980 participants in 11 studies^24,30,34,38,43,45,48,56,60,62,66^; 27 events	−2 (−19 to 14)	Low^b^
Sleep problems	1525 participants in 12 studies^24,32,34,38,43,45,48,56,59,60,62,66^; 37 events	15 (1 to 28)	Moderate^e^
Headache	1754 participants in 16 studies^24,27,28,30,32,34,38,43,45,48,49,51,56,60,62,66^; 59 events	−6 (−20 to 9)	Moderate^a^
Change in behavior	1448 participants in 13 studies^24,30,32,33,34,38,43,45,48,51,60,62,66^; 24 events	8 (−5 to 21)	Moderate^a^
Convulsion or seizure	482 participants in 10 studies^34,35,38,43,45,48,60,62,65,66^; 6 events	17 (−3 to 36)	Low^b^
Febrile convulsion	1326 participants in 10 studies^26,34,38,43,45,48,60,62,64,66^; 2 events	3 (−1 to 6)	Low^b^
Irritability	1451 participants in 10 studies^26,30,34,38,43,45,48,60,62,66^; 4 events	0 (−5 to 5)	Low^b^
Dizziness	566 participants in 10 studies^34,38,43,45,48,51,60,62,66,68^; 1 event	−4 (−10 to 3)	Very low^b^^,^^e^
Tremor and/or hyperactivity	1412 participants in 11 studies^28,31,32,34,38,43,45,48,60,62,66^; 22 events	4 (−8 to 16)	Moderate^a^
Fatigue	917 participants in 10 studies^24,32,34,38,43,45,48,60,62,66^; 7 events	−2 (−13 to 9)	Low^b^
Local or injection site pain	561 participants in 10 studies^34,38,43,45,46,48,60,62,63,66^; 4 events	0 (−14 to 14)	Very low^b^^,^^e^
Candidiasis	503 participants in 10 studies^34,38,43,45,48,56,60,62,63,66^; 4 events	8 (−7 to 23)	Very low^b^^,^^e^
Pneumonia	1481 participants in 13 studies^26,34,35,36,38,43,45,48,60,62,64,65,66^; 54 events	4 (−12 to 19)	Low^b^
Secondary (opportunistic) infection	929 participants in 12 studies^25,30,34,38,42,43,45,48,60,62,66,67^; 14 events	8 (−7 to 24)	Low^b^
Otitis media	1164 participants in 10 studies^26,34,38,43,45,48,60,62,65,66^; 8 events	−7 (−16 to 3)	Low^b^
Urinary tract infection	1186 participants in 11 studies^26,34,35,36,38,43,45,48,60,62,66^; 26 events	2 (−11 to 15)	Low^b^
Musculoskeletal pain	Based on data from 1080 participants in 10 studies^30,32,34,38,43,45,48,60,62,66^; 2 events	−4 (−9 to 1)	Very low^b^^,^^e^
Myalgia	693 participants in 10 studies^24,34,38,43,45,48,51,60,62,66^; 1 event	3 (−3 to 9)	Very low^b^^,^^e^
Anemia	701 participants in 11 studies^34,38,43,45,48,49,56,60,62,63,66^; 28 events	−12 (−35 to 12)	Low^b^
Hypertension	1270 participants in 16 studies^25,30,33,34,38,43,45,48,50,53,60,61,62,64,66,67^; 5 events	1 (−6 to 9)	Low^b^
Congestive heart failure	618 participants in 10 studies^34,38,43,45,48,49,60,61,62,66^; 2 events	−5 (−15 to 3)	Very low^b^^,^^e^
Rash and urticaria	3098 participants in 20 studies^24,26,29,31,32,34,37,38,43,45,46,48,54,56,60,62,63,64,66,68^; 24 events	−3 (−9 to 3)	Moderate^a^

^a^ Imprecision: serious; confidence intervals crossing null effect (risk difference = 0).

^b^ Imprecision: very serious; confidence intervals crossing null effect (risk difference = 0) and a small number of total events (<30).

^c^ Inconsistency: serious; *P* value for inconsistency = .01; *I*^2^ = 46.1%.

^d^ Indirectness: serious.

^e^ Risk of bias: serious.

^f^ Inconsistency for hyperglycemia: serious; (*I*^2^ = 36.3%; P = 10).

^g^ Inconsistency for glycosuria; serious (*I*^2^ = 87.3%; *P* < .001).

#### SAEs

Nineteen studies,^24,26,27,31,32,34,38,43,45,48,49,50,56,59,60,62,63,64,66^ including 3418 participants, reported data evaluating SAEs. Corticosteroids were not associated with increased risk of SAEs compared with no corticosteroids (RD, 1 fewer AE per 1000 patients [95% CI, 9 fewer to 7 more AEs per 1000 patients]; moderate certainty) (Table 3).

#### AE Leading to Discontinuation

Sixteen studies,^29,31,33,34,37,38,43,45,48,52,58,59,60,62,64,66^ including 2892 participants, reported on AEs leading to drug discontinuation. Corticosteroids were not associated with AEs leading to drug discontinuation (RD, 4 more AEs per 1000 patients [95% CI, 3 fewer to 11 more AEs per 1000 patients]; moderate certainty) (Table 3).

#### Hyperglycemia

Twelve studies,^25,34,38,43,45,48,60,62,63,64,66,67^ including 792 participants, reported on hyperglycemia. Corticosteroids were associated with an increased risk of hyperglycemia (RD, 38 more AEs per 1000 patients [95% CI, 11 to 64 more AEs per 1000 patients]; moderate certainty) (Table 3).

#### Sleep Problems

Twelve studies,^24,32,34,38,43,45,48,56,59,60,62,66^ including 1525 children, reported on sleep problems. Corticosteroids were associated with an increased risk of sleep problems (RD, 15 more AEs per 1000 patients [95% CI, 1 to 28 more AEs per 1000 patients]; moderate certainty) (Table 3).

#### Change in Behavior

Thirteen studies,^24,30,32,33,34,38,43,45,48,51,60,62,66^ including 1448 children, reported on change in behavior. Corticosteroids were not associated with an increased risk of change in behavior compared with no corticosteroids (RD, 8 more AEs per 1000 patients [95% CI, 5 fewer to 21 more AEs per 1000 patients]; moderate certainty) (Table 3). Of the 13 trials, only 4 trials reported how changes in behavior were captured: 3 trials^24,32,51^ relied on self-report or reports from legal guardians, and 1 trial^30^ was based on clinician assessment.

#### Gastrointestinal Bleeding

Twenty-two studies,^25,26,30,33,34,35,36,38,39,40,41,43,44,45,48,55,58,60,62,63,64,66^ including 2555 participants, reported on gastrointestinal bleeding. The findings suggest that corticosteroids are associated with an increased risk of gastrointestinal bleeding (RD, 13 more AEs per 1000 patients; [95% CI, 3 to 23 more AEs per 1000 patients]), but the certainty of evidence was low (Table 3).

#### Subgroup and Sensitivity Analyses

For gastrointestinal bleeding, we found a statistically significant subgroup association based on the route of administration (intravenous or intramuscular: RD, 35 more per AEs per 1000 patients [95% CI, 9 to 61 more AEs per 1000 patients]; oral: RD, 1 fewer AE per 1000 patients [95% CI, 9 fewer to 8 more AEs per 1000 patients]; *P* = .01). However, this association had low credibility using the ICEMAN criteria (eTable 2 and eFigure 3 in Supplement 1). Reasons for low credibility were between-trial analysis comparison and use of a fixed-effects model. We did not observe any other significant subgroup associations based on dose, duration, or clinical condition (eFigure 3 in Supplement 1). Sensitivity analyses using the Peto OR showed similar results to those of the primary analyses. (eFigure 4 in Supplement 1).

## Discussion

This systematic review and meta-analysis found moderate certainty evidence that using systemic corticosteroids for a short duration (≤14 days) in a pediatric population was associated with a small increase in the risk of nonserious hyperglycemia and sleep problems. Corticosteroids were also associated with an increased risk of gastrointestinal bleeding, but the certainty of evidence was low. Our results also demonstrated that corticosteroids were not associated with the risk of SAEs.

A previous systematic review^7^ investigated the AEs of short-course oral corticosteroids in children and showed similar results in terms of incidence rates. Vomiting, behavioral changes, and sleep disturbance were among the most frequent AEs. This review^7^ only included oral interventions, excluded studies at high risk of bias, failed to assess the certainty of evidence, included observational studies, and did not perform meta-analyses. Our review confirms that those AEs were among the most frequent, but—even more important to patients and clinicians—it found that corticosteroids were associated with an increased risk of sleep problems. This prior review^7^ unfoundedly highlighted an increase in infection rates, whereas we found that evidence regarding infections was only low to very low certainty evidence and failed to support an increase in the risk of any infection (Table 3). Other systematic reviews on corticosteroid-associated AEs,^6,69^ also reported that corticosteroids are not associated with the risk of secondary infections.

Several findings from our study align with results from other systematic reviews and meta-analyses in adult and pediatric populations. Previous reviews, one investigating the AEs of corticosteroids in children with acute respiratory conditions^6^ and the other investigating the metabolic AEs of corticosteroids in adults,^70^ documented an increased risk of change in behavior and hyperglycemia.

Our results from gastrointestinal bleeding, on the other hand, differ from prior reviews. Fernandes and colleagues^6^ conducted a systematic review on the safety of systemic corticosteroids across acute respiratory conditions and did not report an increase in the risk of gastrointestinal bleeding. This finding might be explained by the inclusion of observational studies leading to biased estimates and restriction to respiratory clinical syndromes in their eligibility criteria. On the other hand, an observational study including a cohort of 23 million individuals in Taiwan^71^ also found an increase in the incidence of gastrointestinal bleeding (incidence rate ratio, 2.02 [95% 1.55-2.64]) with the use of short-term corticosteroids.

Even though we found no credible subgroup association based on clinical condition, a significant proportion of gastrointestinal bleeding events happened in trials involving patients with central nervous system infections (mostly meningitis). The role of adjunctive corticosteroids in pediatric bacterial meningitis, however, remains controversial. The latest Cochrane review^72^ suggests no benefit of corticosteroids on the risk of hearing loss and mortality in pediatric non-Haemophilus influenzae meningitis, which raises concerns about the risk-benefit balance of routine corticosteroid use in this population. Current guidelines^73,74^ advocate for systemic corticosteroids, yet they do not sufficiently address potential adverse effects, particularly the increased risk of gastrointestinal complications. Given the lack of benefit and the underrecognized harms, a more cautious, individualized approach may be warranted. Research to better evaluate the association of corticosteroids with gastrointestinal bleeding, not only on central nervous system infections, but across different conditions, is necessary. eTable 3 in Supplement 1 presents a summary of previous systematic reviews.

The importance of our work is that by looking across clinical conditions we were able to achieve sample sizes that were sufficient to result in sufficiently narrow confidence intervals to provide moderate certainty evidence of steroid associations with AE. Our research shows that corticosteroids—even when prescribed for a short period of time—were not harmless but rather were associated with an increased risk of AEs, including gastrointestinal bleeding, sleep disturbance, and hyperglycemia. The magnitude of the association was, however, small—less than 4% for hyperglycemia and less than 1.5% for the other outcomes. These results can inform both clinical practice guidelines and clinical practice. The impact on decision-making will depend on the importance patients and families place on the small increase in relatively minor AEs.

The methodology used in this systematic review and meta-analysis—pooling data on AEs reported in RCTs regardless of clinical condition—should be widely used for medications, such as corticosteroids, with several clinical indications. Future research should not only focus on similar reviews for other classes of medications but also on developing methodological guidance for those interested in conducting their own reviews.

### Limitations

The limitations of our study relate to limitations in AE reporting in RCTs.^75^ AE reporting was not a primary focus of the included studies, and authors seldom report either the definition of AEs or the methods used to capture them (eTable 4 in Supplement 1). Gastrointestinal bleeding was not reported separately for upper and lower events; therefore, subgroup analysis based on the anatomical site of bleeding could not be conducted. In addition, underreporting of AEs was likely, further contributing to the potential underestimation of harm.

## Conclusions

This systematic review and meta-analysis of short-course use of corticosteroids in pediatric populations found that corticosteroids were associated with an increased risk of hyperglycemia and sleep problems, but these AEs were very seldom, if ever serious. Future research should not only focus on similar reviews for other classes of medications but also on developing methodological guidance.

## Notes

Supplement 1. **eMethods.** Search Strategy **eFigure 1.** Forest Plots **eFigure 2.** Funnel Plots **eTable 1.** Non-Poolable Adverse Events **eTable 2.** Instrument to Assess the Credibility of Effect Modification Analyses (ICEMAN) Version 1.1; Outcome: Gastrointestinal Bleeding **eFigure 3.** Subgroup Analyses **eFigure 4.** Sensitivity Analyses: Peto Odds Ratio **eTable 3.** Previous Systematic Reviews **eTable 4.** Method Used to Capture Adverse Events in Included Studies

Supplement 2.
Data Sharing Statement
