# Supplementary material for: Adverse Events Following Short-Course Systemic Corticosteroids Among Children and Adolescents: A Systematic Review and Meta-Analysis
Source: JAMA Netw Open. 2025 Sep 30;8(9):e2534953. doi: 10.1001/jamanetworkopen.2025.34953 (PMC12485646; doi:10.1001/jamanetworkopen.2025.34953)
Supplement: Supplement 1. — eMethods. Search Strategy eFigure 1. Forest Plots eFigure 2. Funnel Plots eTable 1. Non-Poolable Adverse Events eTable 2. Instrument to Assess the Credibility of Effect Modification Analyses (ICEMAN) Version 1.1; Outcome: Gastrointestinal Bleeding eFigure 3. Subgroup Analyses eFigure 4. Sensitivity Analyses: Peto Odds Ratio eTable 3. Previous Systematic Reviews eTable 4. Method Used to Capture Adverse Events in Included Studies [file jamanetwopen-e2534953-s001.pdf]

Supplemental Online Content

Lima JP, Chowdhury SR, Tangamornsuksan W, et al. Adverse events following short-course systemic corticosteroids among children and adolescents: a systematic review and meta-analysis. *JAMA Netw. Open*. 2025;8(10):e2534953. doi:10.1001/jamanetworkopen.2025.34953

eMethods. Search Strategy

eFigure 1. Forest Plots

eFigure 2. Funnel Plots

eTable 1. Non-Poolable Adverse Events

eTable 2. Instrument to Assess the Credibility of Effect Modification Analyses (ICEMAN) Version 1.1; Outcome: Gastrointestinal Bleeding

eFigure 3. Subgroup Analyses

eFigure 4. Sensitivity Analyses: Peto Odds Ratio

eTable 3. Previous Systematic Reviews

eTable 4. Method Used to Capture Adverse Events in Included Studies

This supplemental material has been provided by the authors to give readers additional information about their work.

© 2025 Lima JP et al. *JAMA Network Open*.

eMethods. Search Strategy

Ovid MEDLINE(R) and Epub Ahead of Print, In-Process, In-Data-Review & Other Non-Indexed Citations, Daily and Versions 1946 to February, 2025

#	**Query**
1	exp Glucocorticoids/
2	(steroid* or corticosteroid* or glucocorticoid* or prednisolone* or dexamethasone* or hydrocortisone* or methylprednisolone*).mp.
3	1 or 2
4	exp Child/
5	(child* or stepchild* or step-child* or kid or kids or girl or girls or boy or boys or teen* or youth* or youngster* or adolescent* or adolescence or preschool* or pre-school* or kindergarten* or school* or juvenile* or minors or p?ediatric*).mp.
6	4 or 5
7	exp "Drug-Related Side Effects and Adverse Reactions"/
8	(ae or to or po or co).fs.
9	(safe or safety).ti,ab.
10	side effect$.ti,ab.
11	((adverse or undesirable or harms$ or serious or toxic) adj3 (effect$ or reaction$ or event$ or outcome$)).ti,ab.
12	exp Product Surveillance, Postmarketing/
13	adverse drug reaction reporting systems/
14	exp Poisoning/
15	exp Substance-Related Disorders/
16	Abnormalities, Drug-Induced/

© 2025 Lima JP et al. *JAMA Network Open*.

17	Drug Monitoring/
18	(toxicity or complication$ or noxious or tolerability).ti,ab.
19	or/7-18
20	(ae or de or co or ci).fs.
21	safe*.ti,ab.
22	(safe* or adverse or side effect* or complication* or tolerated or tolerance or harm or toxicity).ti,ab.
23	risk.ti.
24	tolerability.ti,ab.
25	to.fs.
26	Toxicology/
27	drug induced.ti,ab.
28	negative effects.ti,ab.
29	or/20-28
30	19 or 29
31	randomized controlled trial.pt.
32	controlled clinical trial.pt.
33	randomi?ed.ab.
34	placebo.ab.
35	drug therapy.fs.
36	randomly.ab.

© 2025 Lima JP et al. *JAMA Network Open*.

37	trial.ab.
38	groups.ab.
39	or/31-38
40	exp animals/ not humans.sh.
41	39 not 40
42	3 and 6 and 30 and 41
43	3 and 6 and 41
44	(steroid* or corticosteroid* or glucocorticoid* or prednisolone* or dexamethasone* or hydrocortisone* or methylprednisolone*).ti.
45	44 or 1
46	6 and 30 and 41 and 44

Embase 1974 to February, 2025

#	**Query**
1	exp Glucocorticoids/
2	(steroid* or corticosteroid* or glucocorticoid* or prednisolone* or dexamethasone* or hydrocortisone* or methylprednisolone*).mp. [mp=title, abstract, heading word, drug trade name, original title, device manufacturer, drug manufacturer, device trade name, keyword heading word, floating subheading word, candidate term word]
3	1 or 2
4	(steroid* or corticosteroid* or glucocorticoid* or prednisolone* or dexamethasone* or hydrocortisone* or methylprednisolone*).m_titl.
5	exp Child/

© 2025 Lima JP et al. *JAMA Network Open*.

6	(child* or stepchild* or step-child* or kid or kids or girl or girls or boy or boys or teen* or youth* or youngster* or adolescent* or adolescence or preschool* or pre-school* or kindergarten* or school* or juvenile* or minors or p?ediatric*).mp. [mp=title, abstract, heading word, drug trade name, original title, device manufacturer, drug manufacturer, device trade name, keyword heading word, floating subheading word, candidate term word]
7	5 or 6
8	exp "Drug-Related Side Effects and Adverse Reactions"/
9	(ae or to or po or co).fx.
10	(safe or safety).ti,ab.
11	side effect$.ti,ab.
12	((adverse or undesirable or harms$ or serious or toxic) adj3 (effect$ or reaction$ or event$ or outcome$)).ti,ab.
13	exp Product Surveillance, Postmarketing/
14	adverse drug reaction reporting systems/
15	exp Poisoning/
16	exp Substance-Related Disorders/
17	Abnormalities, Drug-Induced/
18	Drug Monitoring/
19	(toxicity or complication$ or noxious or tolerability).ti,ab.
20	safe*.ti,ab.
21	(safe* or adverse or side effect* or complication* or tolerated or tolerance or harm or toxicity).ti,ab.
22	risk.ti.
23	(ae or de or co or ci).fx.
24	tolerability.ti,ab.

© 2025 Lima JP et al. *JAMA Network Open*.

25	to.fx.
26	Toxicology/
27	drug induced.ti,ab.
28	negative effects.ti,ab.
29	or/8-28
30	randomized controlled trial.pt.
31	controlled clinical trial.pt.
32	randomi?ed.ab.
33	placebo.ab.
34	drug therapy.fx.
35	randomly.ab.
36	trial.ab.
37	groups.ab.
38	or/30-37
39	exp human/
40	exp animals/
41	40 not 39
42	38 not 41
43	4 and 7 and 29 and 42
44	limit 43 to "remove medline records"

Cochrane Library CDSR – inception February, 2025

© 2025 Lima JP et al. *JAMA Network Open*.

#1 (steroid* or corticosteroid* or glucocorticoid* or prednisolone* or dexamethasone* or hydrocortisone* or methylprednisolone*):ti

#2 MeSH descriptor: [Child] explode all trees

#3 MeSH descriptor: [Adolescent] explode all trees

#4 (child* or stepchild* or step-child* or kid or kids or girl or girls or boy or boys or teen* or youth* or youngster* or adolescent* or adolescence or preschool* or pre-school* or kindergarten* or school* or juvenile* or minors or p*ediatric*) (Word variations have been searched)

#5 #2 or #3 or #4

#6 ('Drug-Related Side Effects and Adverse Reactions') (Word variations have been searched)

#7 (safe or safety):ti,ab

#8 (side effect$):ti,ab

#9 (adverse or undesirable or harms$ or serious or toxic) and (effect$ or reaction$ or event$ or outcome$):ti,ab

#10 ('Product Surveillance' or Postmarketing):ti,ab

#11 MeSH descriptor: [Poisoning] explode all trees

#12 MeSH descriptor: [Drug-Related Side Effects and Adverse Reactions] explode all trees

#13 MeSH descriptor: [Abnormalities, Drug-Induced] explode all trees

#14 MeSH descriptor: [Adverse Drug Reaction Reporting Systems] explode all trees

#15 MeSH descriptor: [Drug Monitoring] explode all trees

#16 (toxicity or complication$ or noxious or tolerability):ti,ab

#17 safe*:ti,ab

© 2025 Lima JP et al. *JAMA Network Open*.

#18 (safe* or adverse or side effect* or complication* or tolerated or tolerance or harm or toxicity):ti,ab

#19 risk:ti,ab

#20 tolerability:ti,ab

#21 MeSH descriptor: [Toxicology] explode all trees

#22 (drug induced):ti,ab

#23 ('negative effect*'):ti,ab,kw (Word variations have been searched) 58806

#24 (negative effects):ti,ab

#25 70- #23

#26 "randomized controlled trial":ti,ab

#27 "controlled clinical trial":ti,ab

#28 randomi?ed:ti,ab

#29 placebo:ab

#30 "drug therapy":tp

#31 randomly:ab

#32 trial:ab

#33 groups:ab

#34 76- #33

#35 #1 AND #5 AND #25 AND #34

© 2025 Lima JP et al. *JAMA Network Open*.

eFigure1. Forest Plots

1.1 Forest plot – Serious Adverse Events

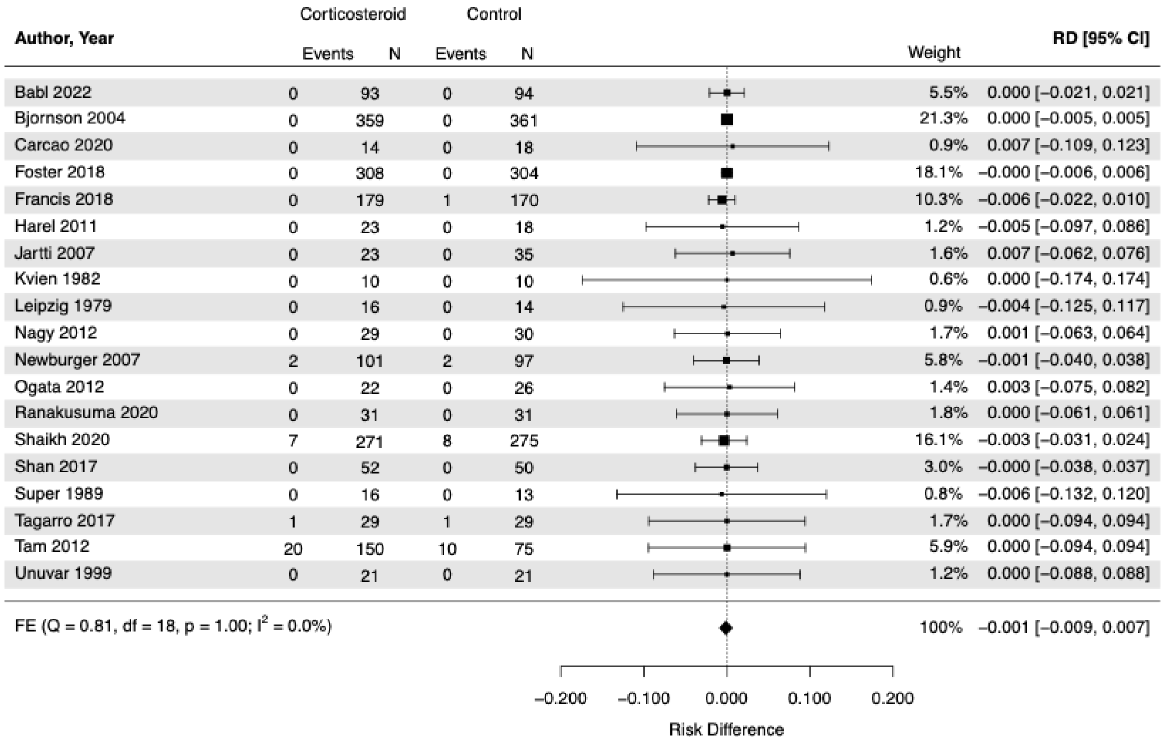

© 2025 Lima JP et al. *JAMA Network Open*.

1.2 Forest plot – Adverse Events Leading to Discontinuation

Author, Year	Corticosteroid Events	Corticosteroid N	Control Events	Control N	Weight	RD [95% CI]
Csonka 2003	11	113	4	117	8.0%	0.063 [−0.001, 0.127]
Foster 2018	1	308	2	304	21.4%	−0.003 [−0.014, 0.008]
Griffin 1994	0	26	0	30	1.9%	0.002 [−0.065, 0.070]
Harel 2011	0	23	0	18	1.4%	−0.005 [−0.097, 0.086]
Huber 2004	0	21	3	19	1.4%	−0.152 [−0.330, 0.026]
Jartti 2007	0	23	0	35	1.9%	0.007 [−0.062, 0.076]
Kvien 1982	0	10	0	10	0.7%	0.000 [−0.174, 0.174]
Leipzig 1979	0	16	0	14	1.0%	−0.004 [−0.125, 0.117]
Nagy 2012	0	29	0	30	2.1%	0.001 [−0.063, 0.064]
Panickar 2009	1	344	0	343	24.0%	0.003 [−0.005, 0.011]
Schaad 1993	0	60	0	55	4.0%	−0.001 [−0.034, 0.033]
Shaikh 2020	0	271	0	275	19.1%	0.000 [−0.007, 0.007]
Shan 2017	0	52	0	50	3.6%	−0.000 [−0.038, 0.037]
Super 1989	0	16	0	13	1.0%	−0.006 [−0.132, 0.120]
Tam 2012	5	150	1	75	7.0%	0.020 [−0.019, 0.059]
Unuvar 1999	0	21	0	21	1.5%	0.000 [−0.088, 0.088]
FE (Q = 10.40, df = 15, p = 0.79; I^2 = 0.0%)					100%	0.004 [−0.003, 0.011]

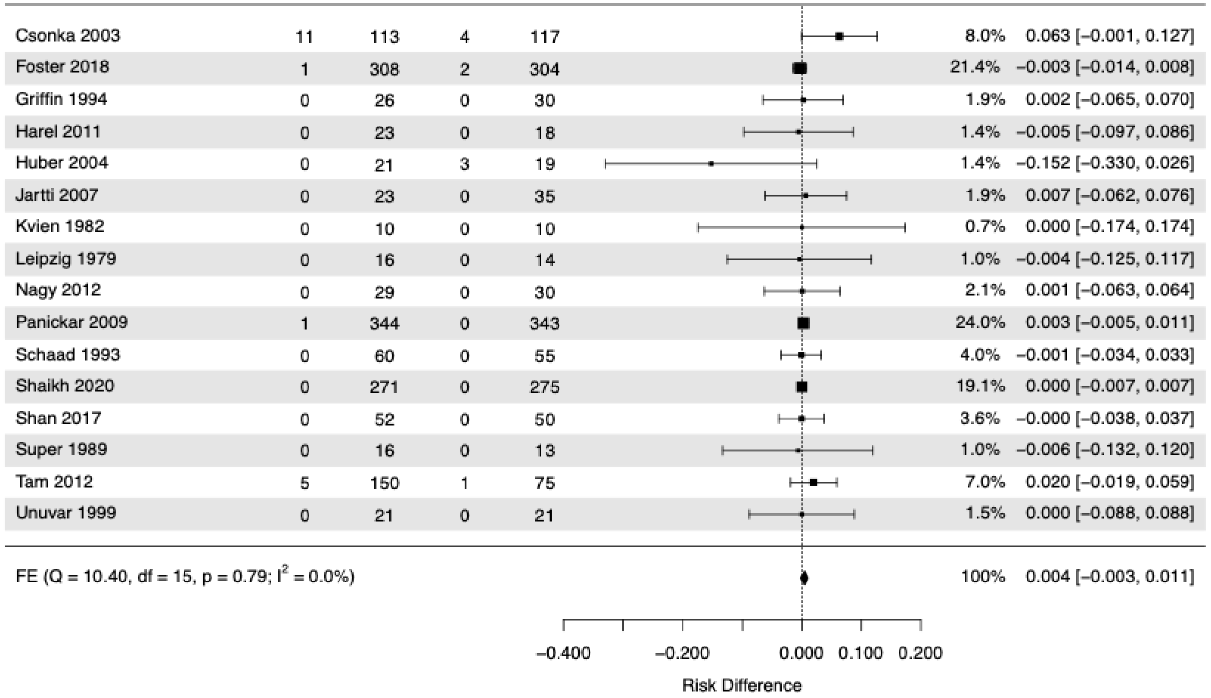

1.3 Forest plot – Abdominal Pain

© 2025 Lima JP et al. *JAMA Network Open*.

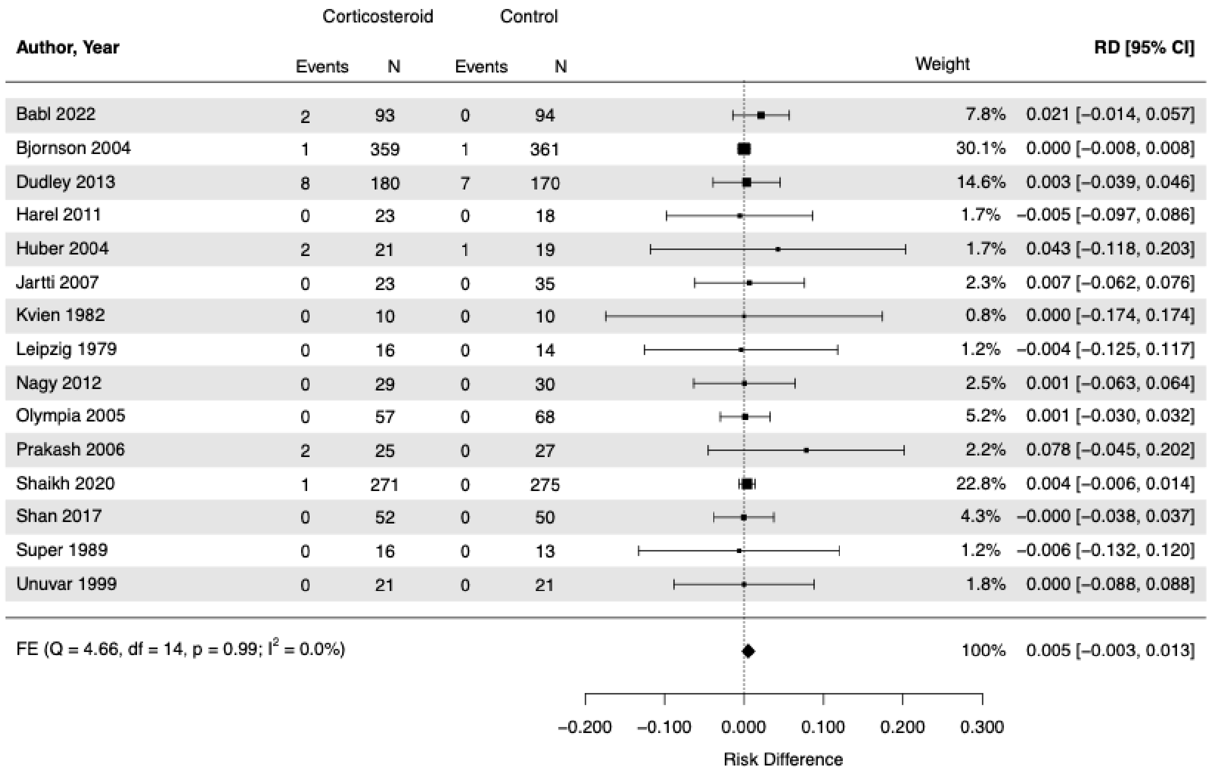

1.3 Forest plot – Diarrhea

© 2025 Lima JP et al. *JAMA Network Open*.

Author, Year	Corticosteroid Events	Corticosteroid N	Control Events	Control N	Weight	RD [95% CI]
Babl 2022	2	93	0	94	5.8%	0.021 [−0.014, 0.057]
Bjornson 2004	3	359	4	361	22.2%	−0.003 [−0.017, 0.012]
Csonka 2003	6	113	6	117	7.1%	0.002 [−0.056, 0.059]
Dudley 2013	1	180	0	170	10.8%	0.005 [−0.010, 0.021]
Francis 2018	2	179	2	170	10.7%	−0.001 [−0.023, 0.022]
Harel 2011	0	23	0	18	1.2%	−0.005 [−0.097, 0.086]
Jartti 2007	0	23	0	35	1.7%	0.007 [−0.062, 0.076]
Kvien 1982	0	10	0	10	0.6%	0.000 [−0.174, 0.174]
Leipzig 1979	0	16	0	14	0.9%	−0.004 [−0.125, 0.117]
Lv 2022	4	60	5	60	3.7%	−0.017 [−0.111, 0.078]
Nagy 2012	0	29	0	30	1.8%	0.001 [−0.063, 0.064]
Ranakusuma 2020	2	31	4	31	1.9%	−0.065 [−0.211, 0.082]
Ruohola 1999	0	27	2	23	1.5%	−0.086 [−0.218, 0.045]
Shaikh 2020	19	271	16	275	16.8%	0.012 [−0.029, 0.053]
Shan 2017	0	52	0	50	3.1%	−0.000 [−0.038, 0.037]
Super 1989	0	16	0	13	0.9%	−0.006 [−0.132, 0.120]
Tam 2012	0	150	1	75	6.2%	−0.016 [−0.049, 0.016]
Unuvar 1999	0	21	0	21	1.3%	0.000 [−0.088, 0.088]
Wang 2022	0	30	1	30	1.8%	−0.032 [−0.120, 0.055]
FE (Q = 6.52, df = 18, p = 0.99; I^2 = 0.0%)					100%	−0.001 [−0.012, 0.009]

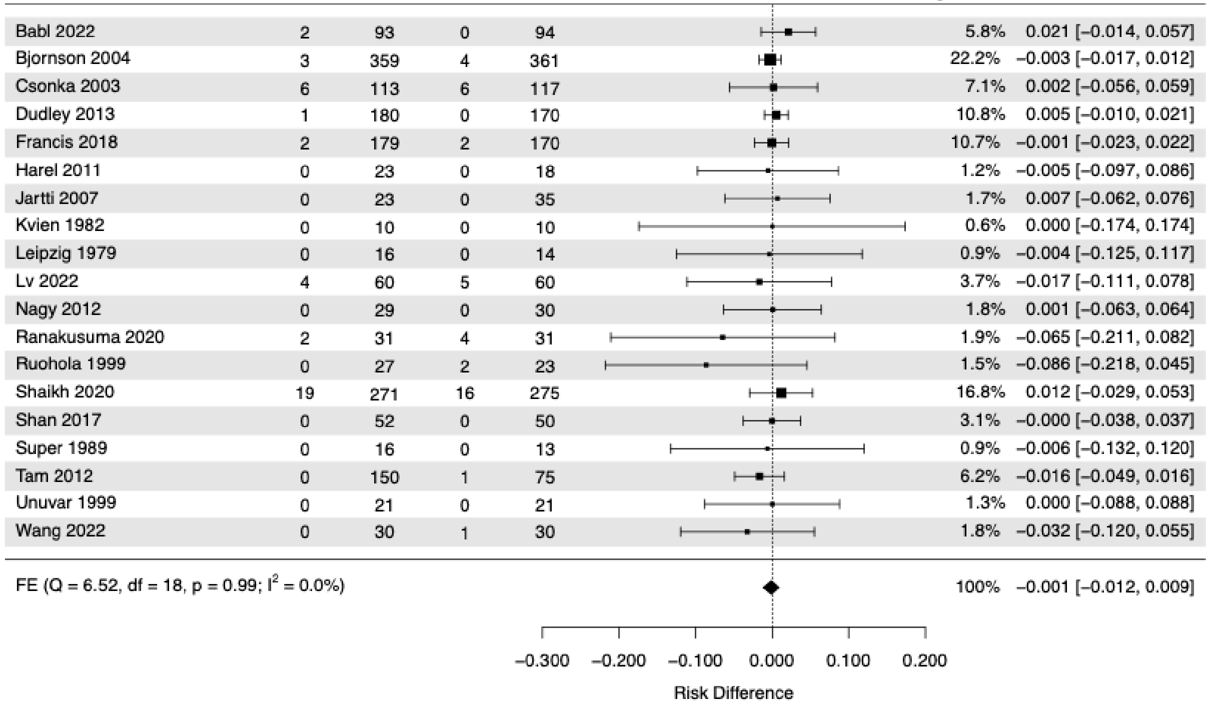

1.4 Forest plot – Gastritis

© 2025 Lima JP et al. *JAMA Network Open*.

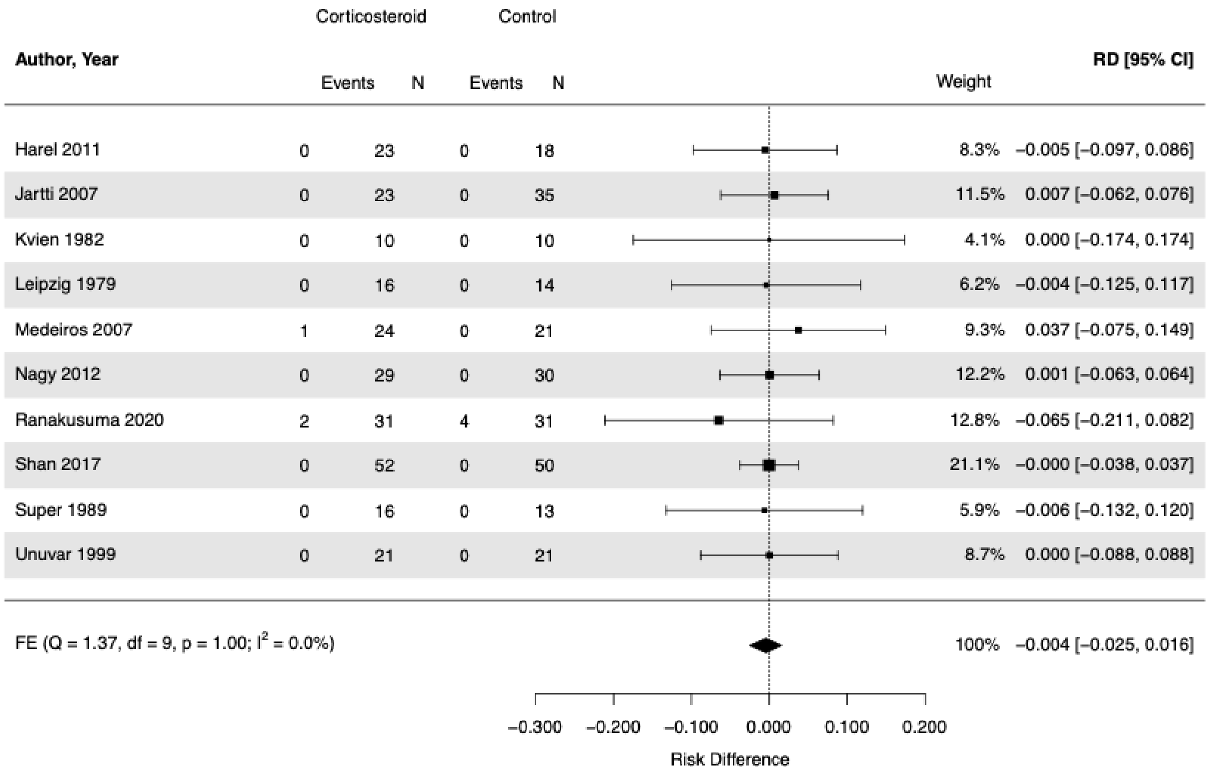

1.5 Forest plot – Gastrointestinal Bleeding

© 2025 Lima JP et al. *JAMA Network Open*.

Author, Year	Corticosteroid Events	Corticosteroid N	Control Events	Control N	Weight	RD [95% CI]
Bernini 1998	0	22	0	21	1.7%	−0.001 [−0.087, 0.085]
Bjornson 2004	0	359	0	361	28.5%	0.000 [−0.005, 0.005]
Dudley 2013	3	180	2	170	13.9%	0.005 [−0.020, 0.030]
Griffin 1994	0	26	0	30	2.2%	0.002 [−0.065, 0.070]
Harel 2011	0	23	0	18	1.6%	−0.005 [−0.097, 0.086]
Hoffman 1988	3	19	0	19	1.5%	0.150 [−0.030, 0.330]
Hoke 1992	12	24	4	27	2.0%	0.352 [0.111, 0.593]
Jartti 2007	0	23	0	35	2.2%	0.007 [−0.062, 0.076]
Johnnson 1998	0	47	0	49	3.8%	0.000 [−0.039, 0.040]
Kanra 1995	0	27	0	26	2.1%	−0.001 [−0.071, 0.070]
King 1994	1	50	1	51	4.0%	0.000 [−0.054, 0.055]
Kvien 1982	0	10	0	10	0.8%	0.000 [−0.174, 0.174]
Lebel 1988	0	98	2	102	7.9%	−0.019 [−0.052, 0.014]
Leipzig 1979	0	16	0	14	1.2%	−0.004 [−0.125, 0.117]
Nagy 2012	0	29	0	30	2.3%	0.001 [−0.063, 0.064]
Qazi 1996	3	48	2	41	3.5%	0.014 [−0.081, 0.109]
Schaad 1993	10	49	5	43	3.6%	0.088 [−0.060, 0.236]
Shan 2017	0	52	0	50	4.0%	−0.000 [−0.038, 0.037]
Super 1989	0	16	0	13	1.1%	−0.006 [−0.132, 0.120]
Tagarro 2017	0	30	0	30	2.4%	0.000 [−0.063, 0.063]
Tam 2012	1	150	0	75	7.9%	0.003 [−0.021, 0.027]
Unuvar 1999	0	21	0	21	1.7%	0.000 [−0.088, 0.088]
FE (Q = 38.93, df = 21, p = 0.01; I^2 = 46.1%)					100%	0.013 [0.003, 0.023]

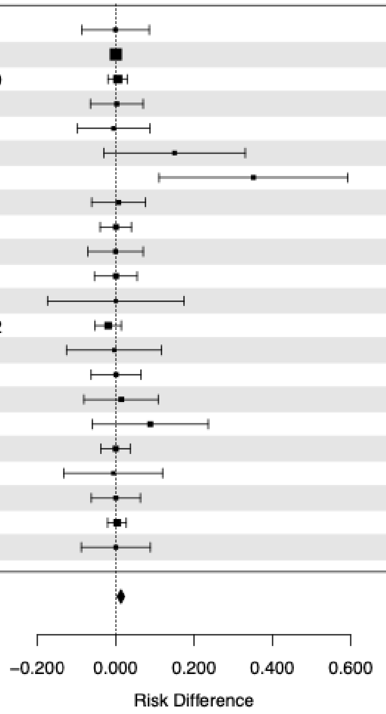

1.6 Forest plot – Hemoccult Positive Stool

© 2025 Lima JP et al. *JAMA Network Open*.

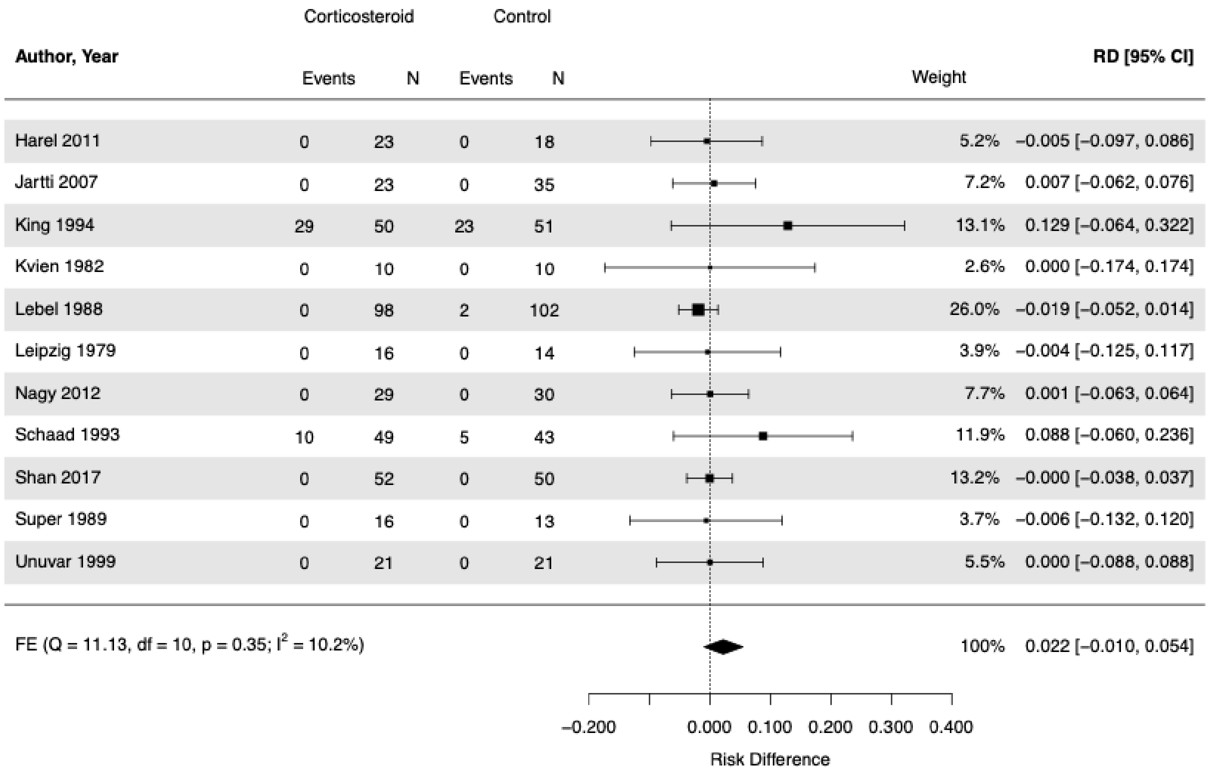

1.7 Forest plot – Intussusception

© 2025 Lima JP et al. *JAMA Network Open*.

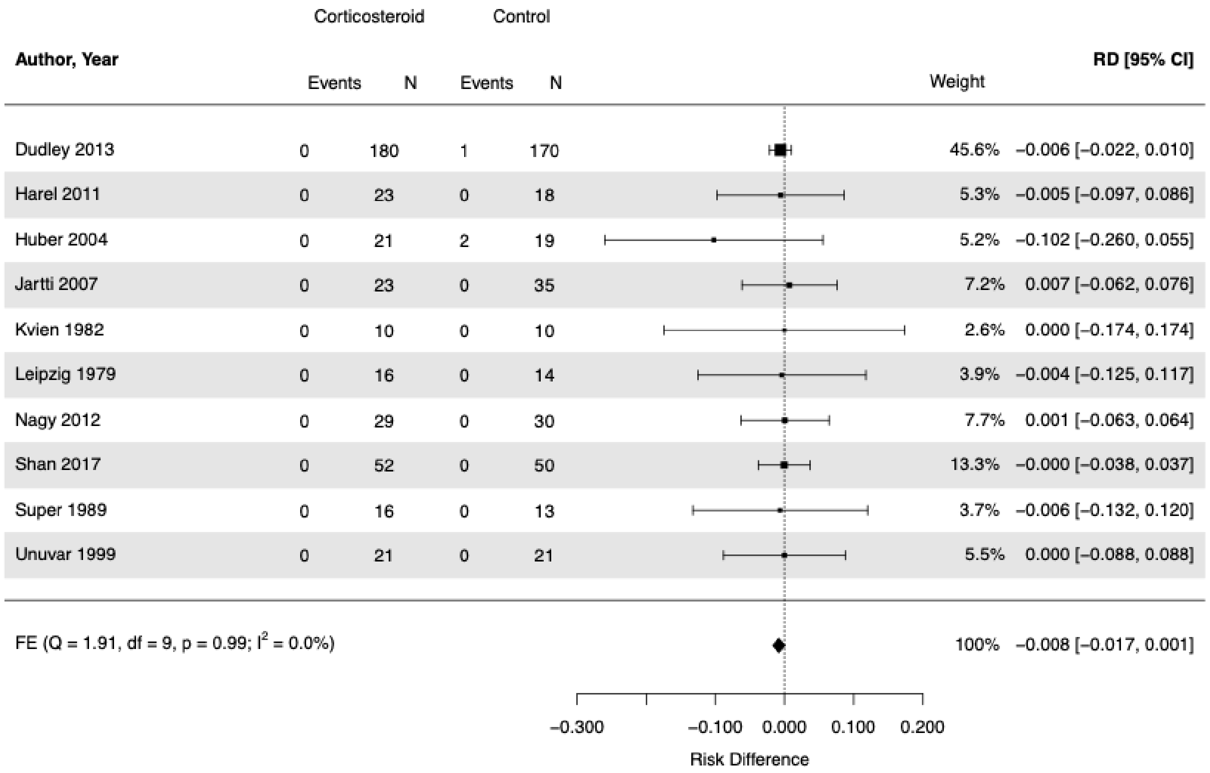

1.8 Forest plot – Nausea

© 2025 Lima JP et al. *JAMA Network Open*.

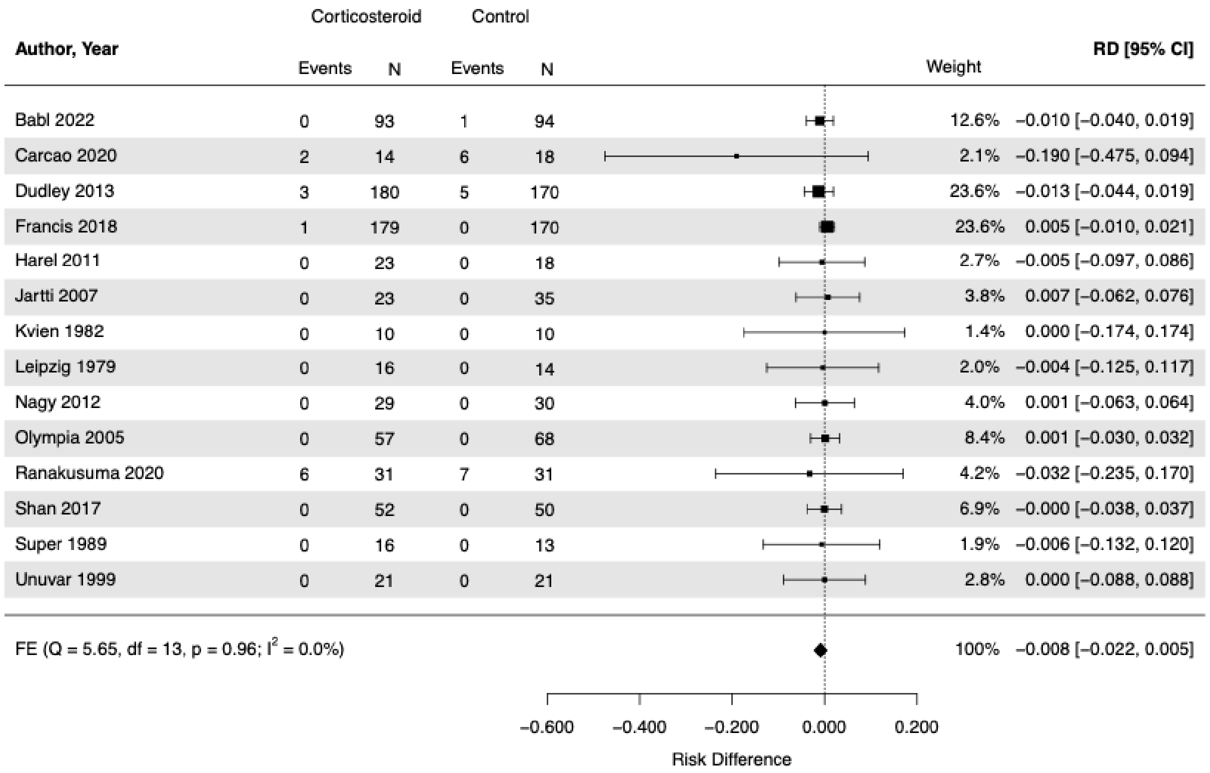

1.9 Forest plot – Vomiting

© 2025 Lima JP et al. *JAMA Network Open*.

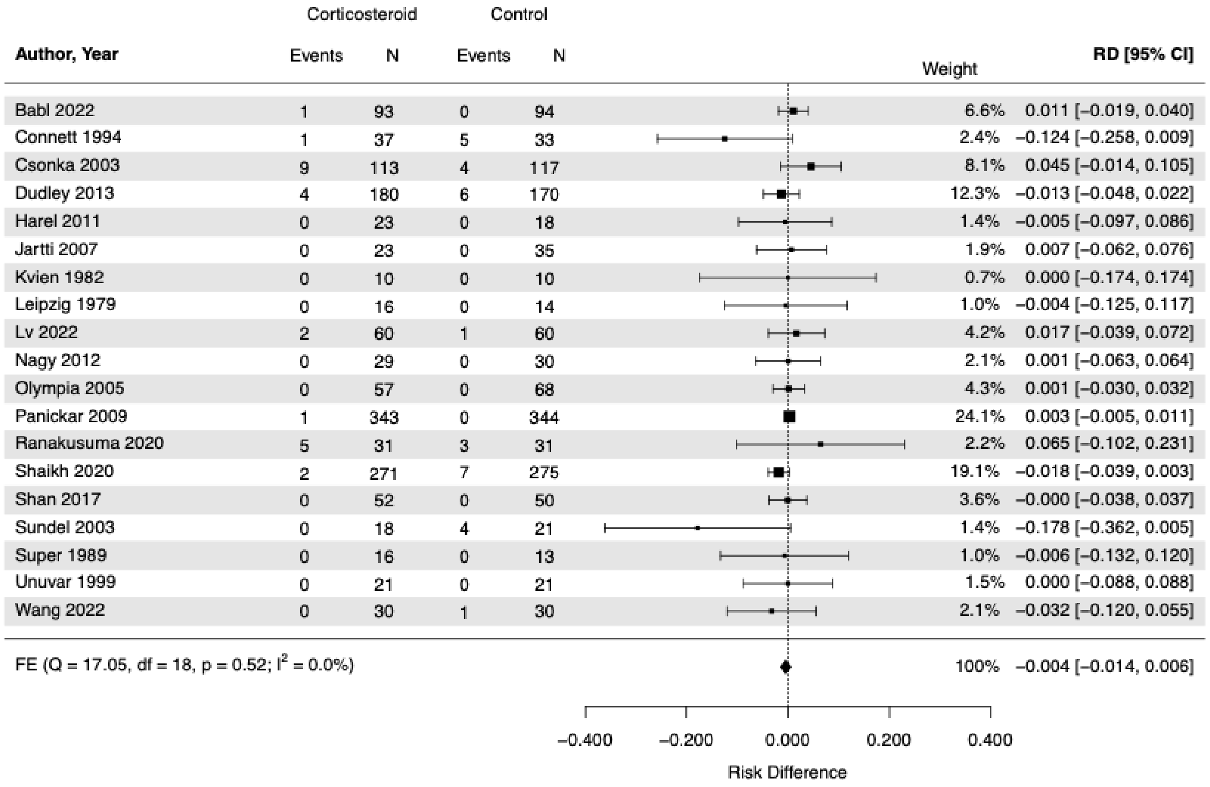

1.10 Forest plot – Anemia

© 2025 Lima JP et al. *JAMA Network Open*.

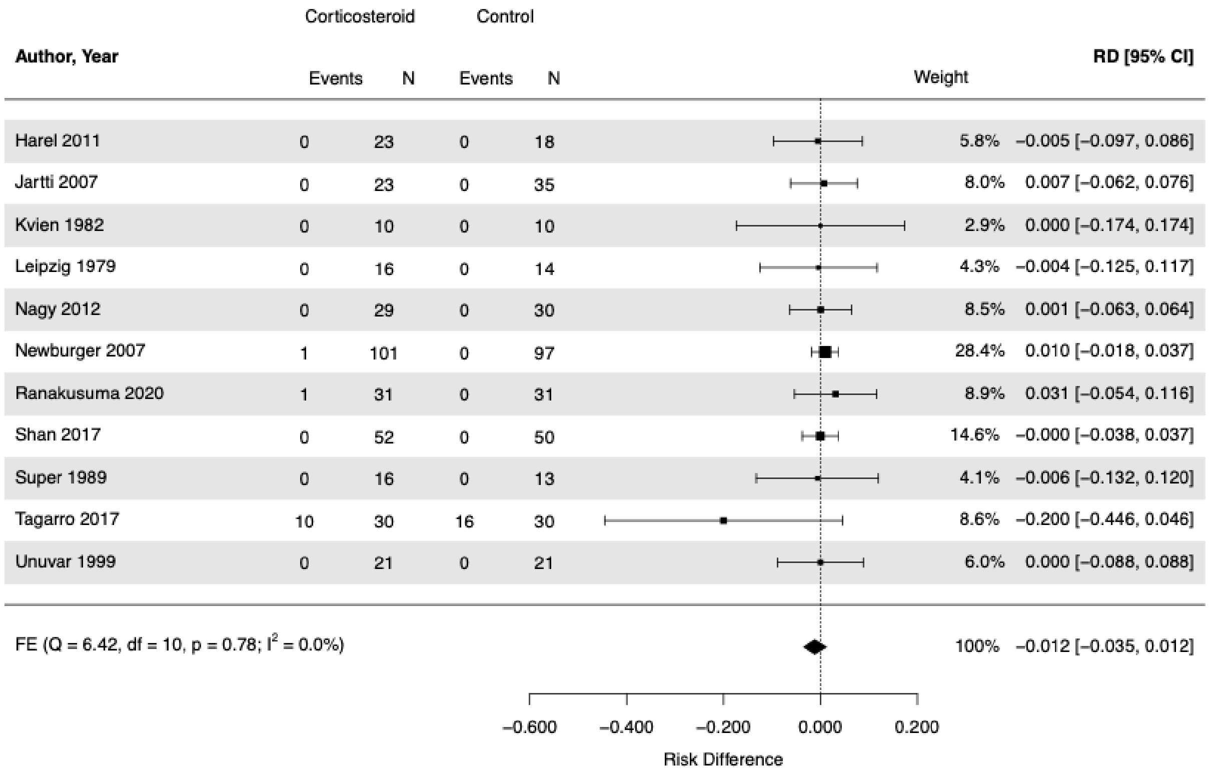

1.11 Forest plot – Candidiasis

© 2025 Lima JP et al. *JAMA Network Open*.

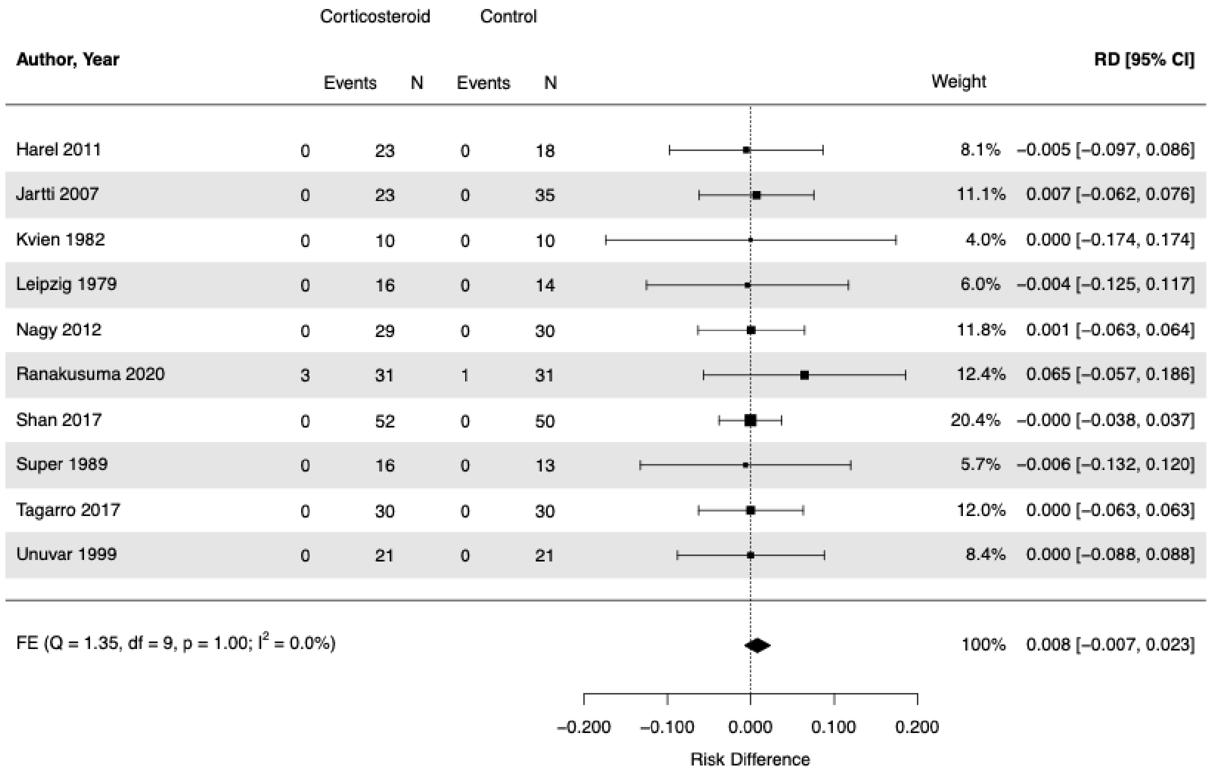

1.12 Forest plot – Change in behaviour

© 2025 Lima JP et al. *JAMA Network Open*.

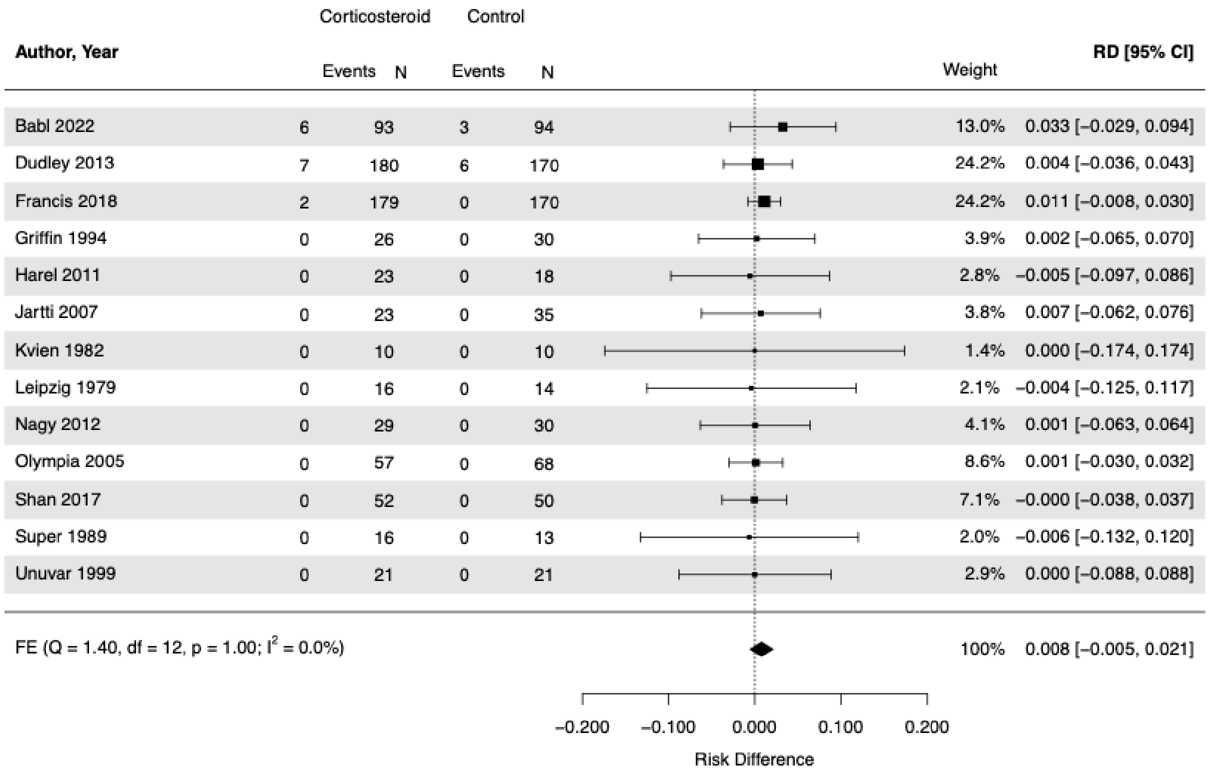

1.13 Forest plot – Congestive Heart Failure

© 2025 Lima JP et al. *JAMA Network Open*.

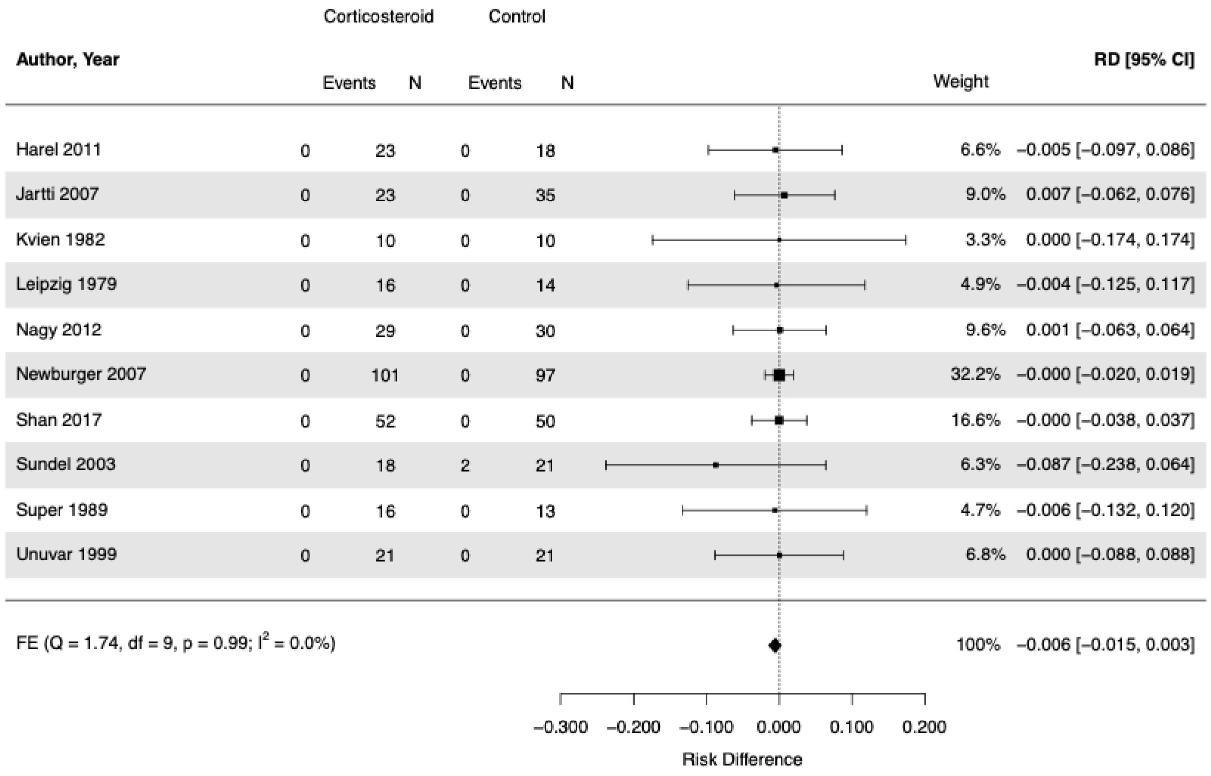

1.14 Forest plot – Convulsion/Seizure

© 2025 Lima JP et al. *JAMA Network Open*.

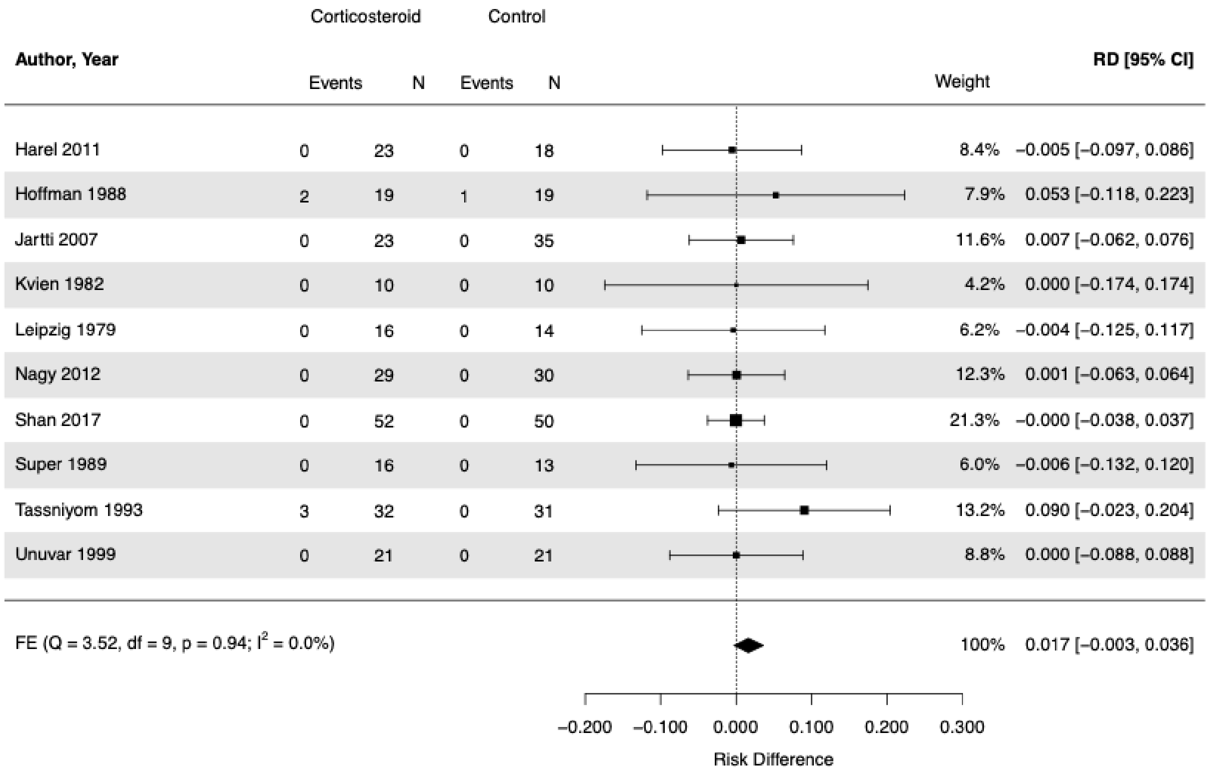

1.15 Forest plot – Decreased Appetite

© 2025 Lima JP et al. *JAMA Network Open*.

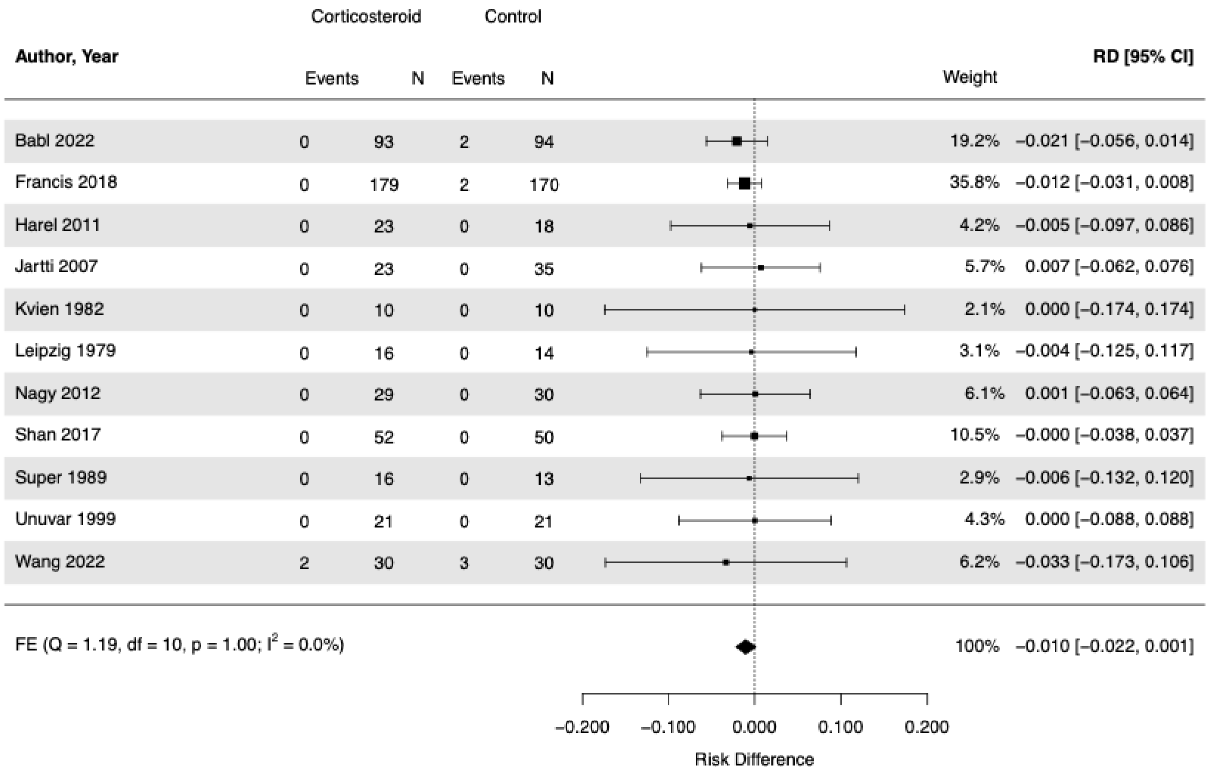

1.16 Forest plot – Dizziness

© 2025 Lima JP et al. *JAMA Network Open*.

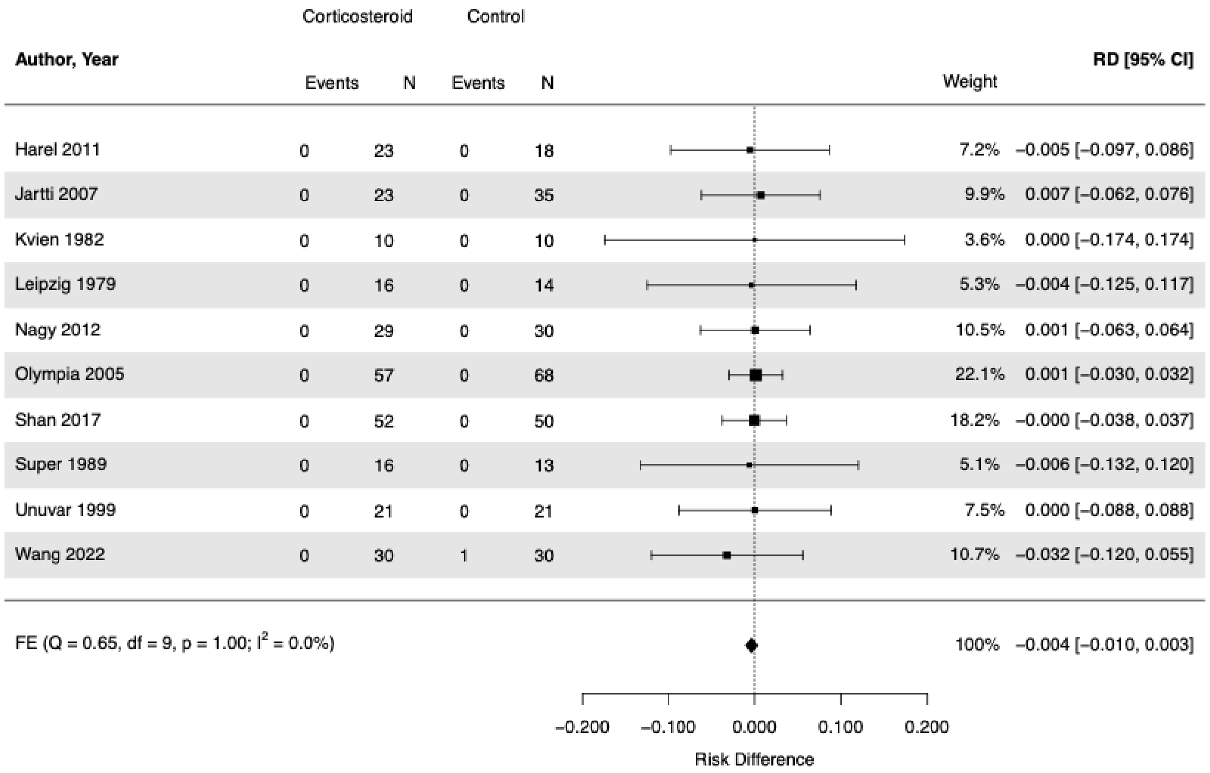

1.17 Forest plot – Fatigue

© 2025 Lima JP et al. *JAMA Network Open*.

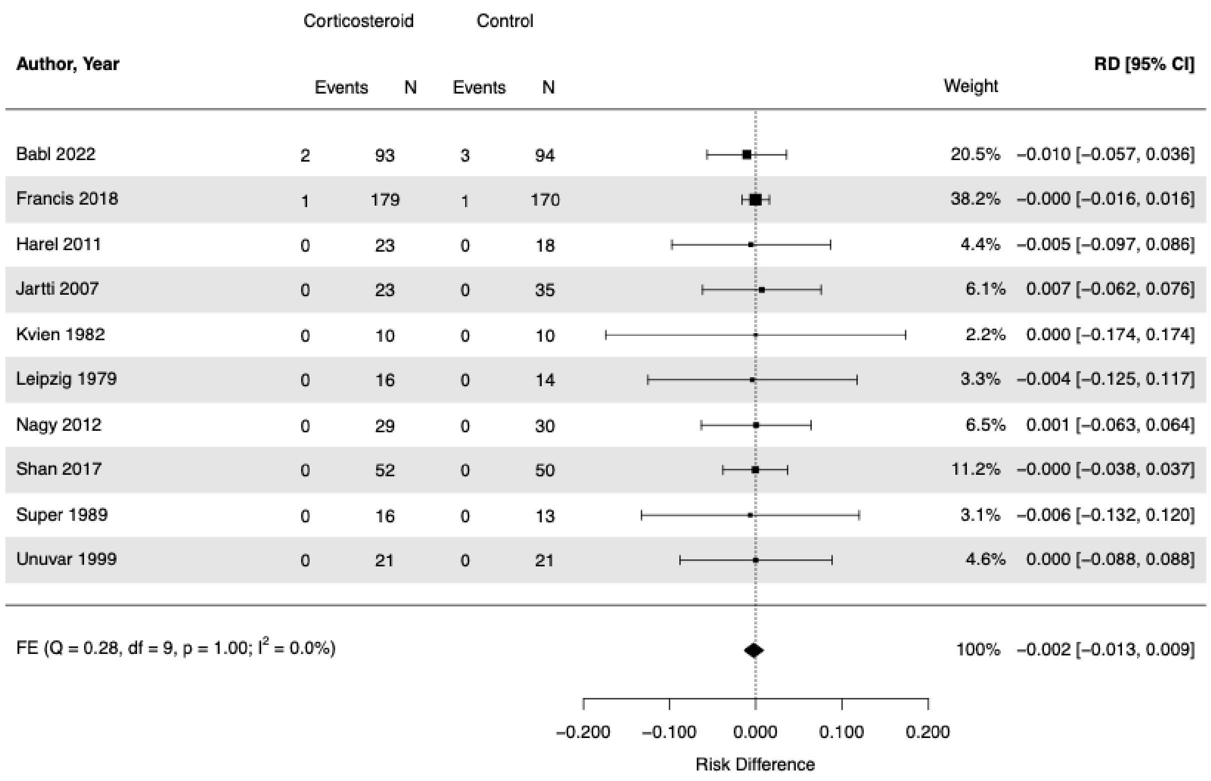

1.18 Forest plot – Febrile Convulsion

© 2025 Lima JP et al. *JAMA Network Open*.

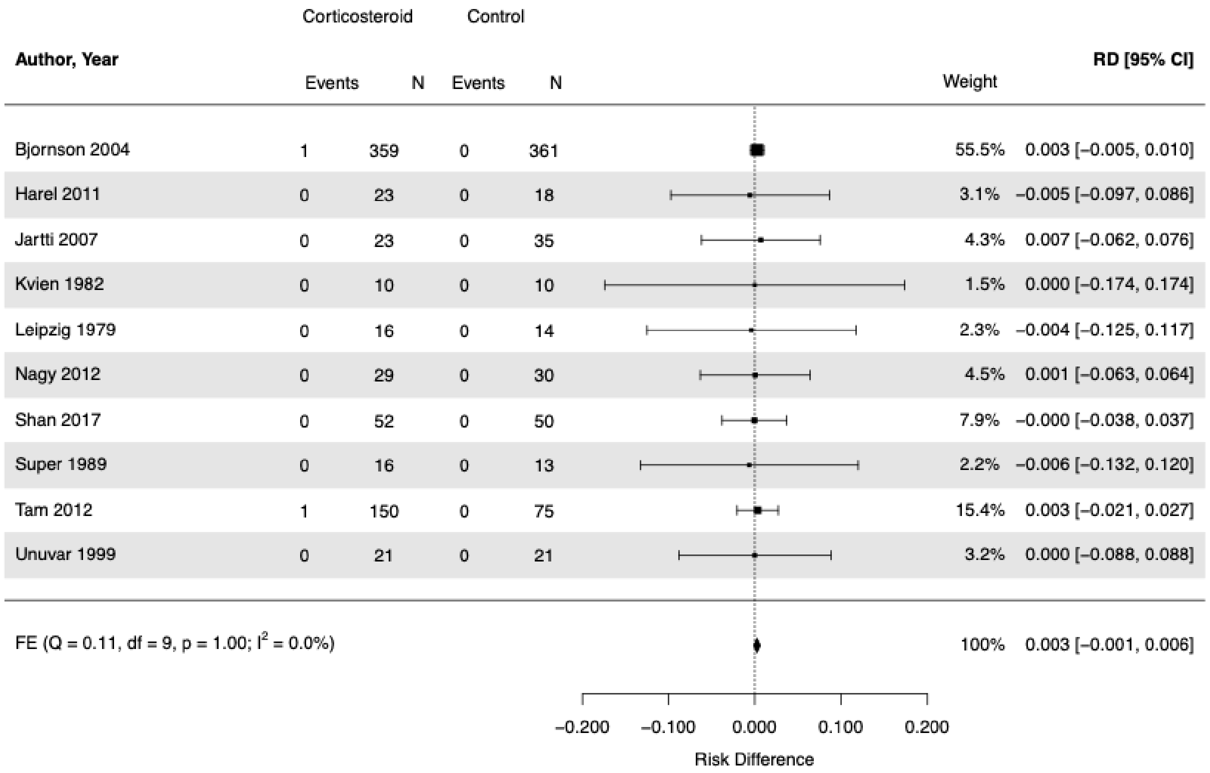

1.19 Forest plot – Glycosuria

© 2025 Lima JP et al. *JAMA Network Open*.

Author, Year	Corticosteroid Events	Corticosteroid N	Control Events	Control N	Weight	RD [95% CI]
Dudley 2013	0	180	0	170	45.2%	−0.000 [−0.011, 0.011]
Harel 2011	0	23	0	18	5.2%	−0.005 [−0.097, 0.086]
Hoke 1992	15	20	4	27	5.9%	0.602 [0.370, 0.834]
Jartti 2007	0	23	0	35	7.2%	0.007 [−0.062, 0.076]
Kvien 1982	0	10	0	10	2.6%	0.000 [−0.174, 0.174]
Leipzig 1979	0	16	0	14	3.9%	−0.004 [−0.125, 0.117]
Nagy 2012	0	29	0	30	7.6%	0.001 [−0.063, 0.064]
Shan 2017	0	52	0	50	13.2%	−0.000 [−0.038, 0.037]
Super 1989	0	16	0	13	3.7%	−0.006 [−0.132, 0.120]
Unuvar 1999	0	21	0	21	5.4%	0.000 [−0.088, 0.088]
FE (Q = 70.66, df = 9, p < .01; I^2 = 87.3%)					100%	0.036 [0.016, 0.055]

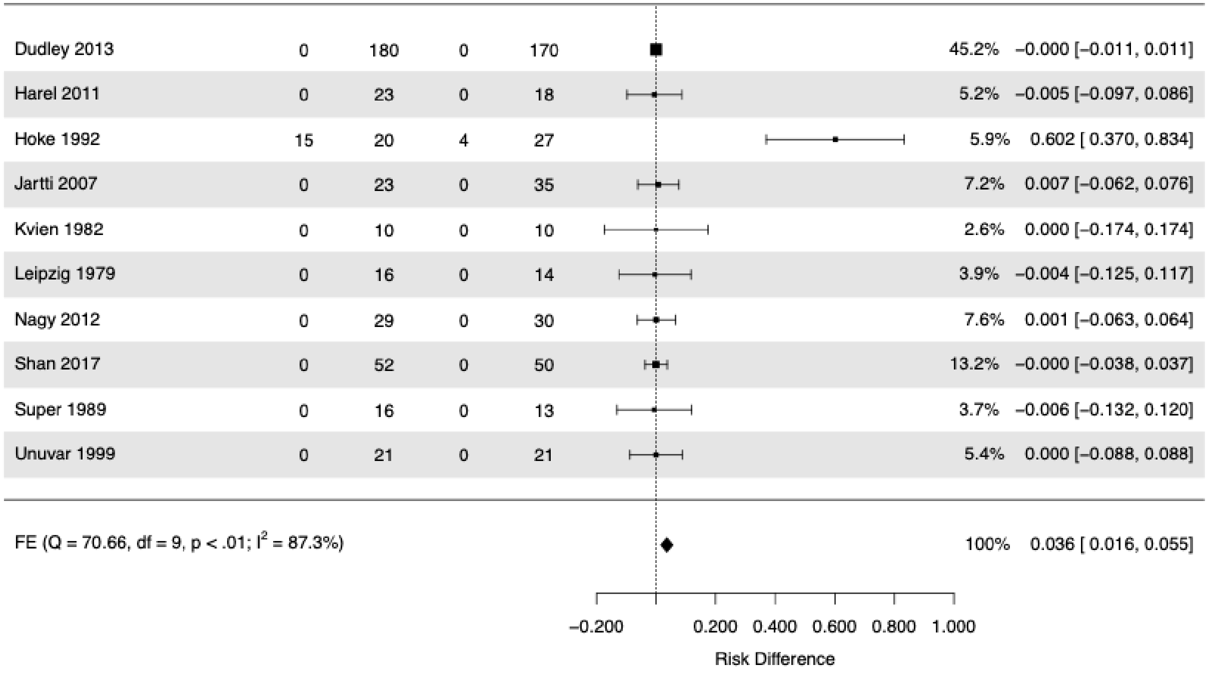

1.20 Forest plot – Headache

© 2025 Lima JP et al. *JAMA Network Open*.

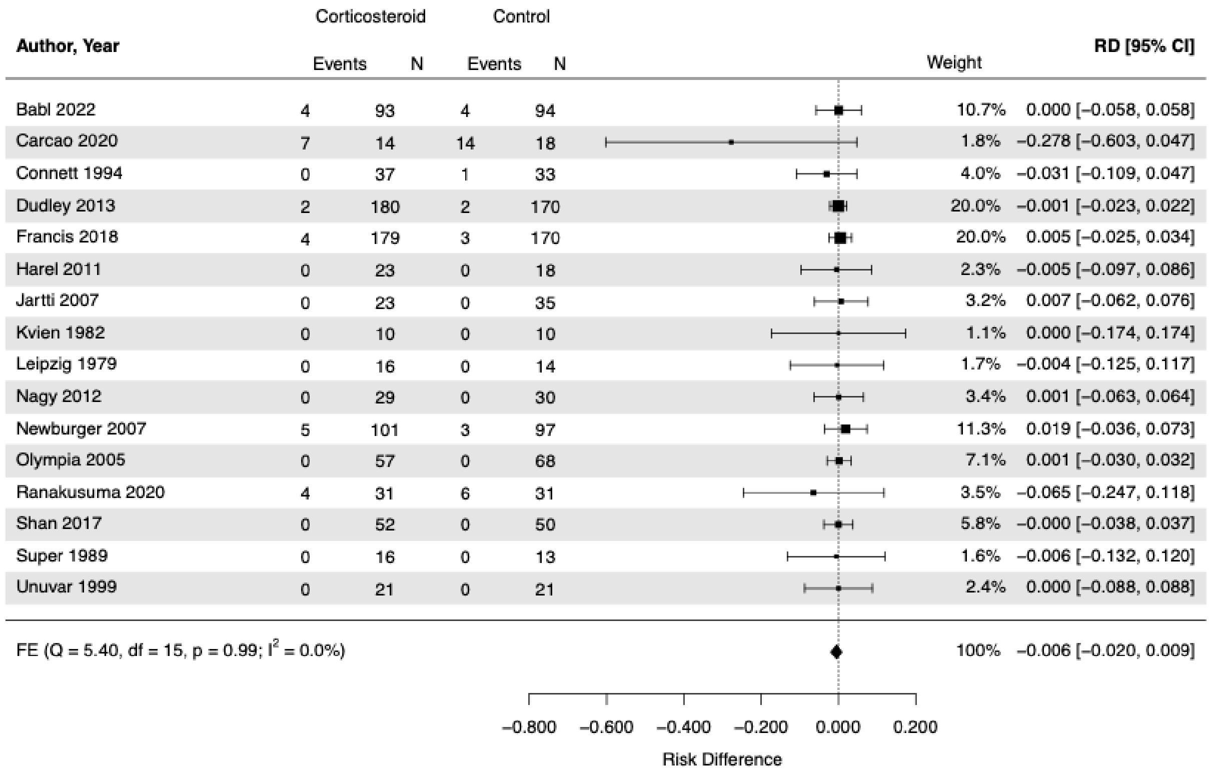

1.21 Forest plot – Hyperglycemia

© 2025 Lima JP et al. *JAMA Network Open*.

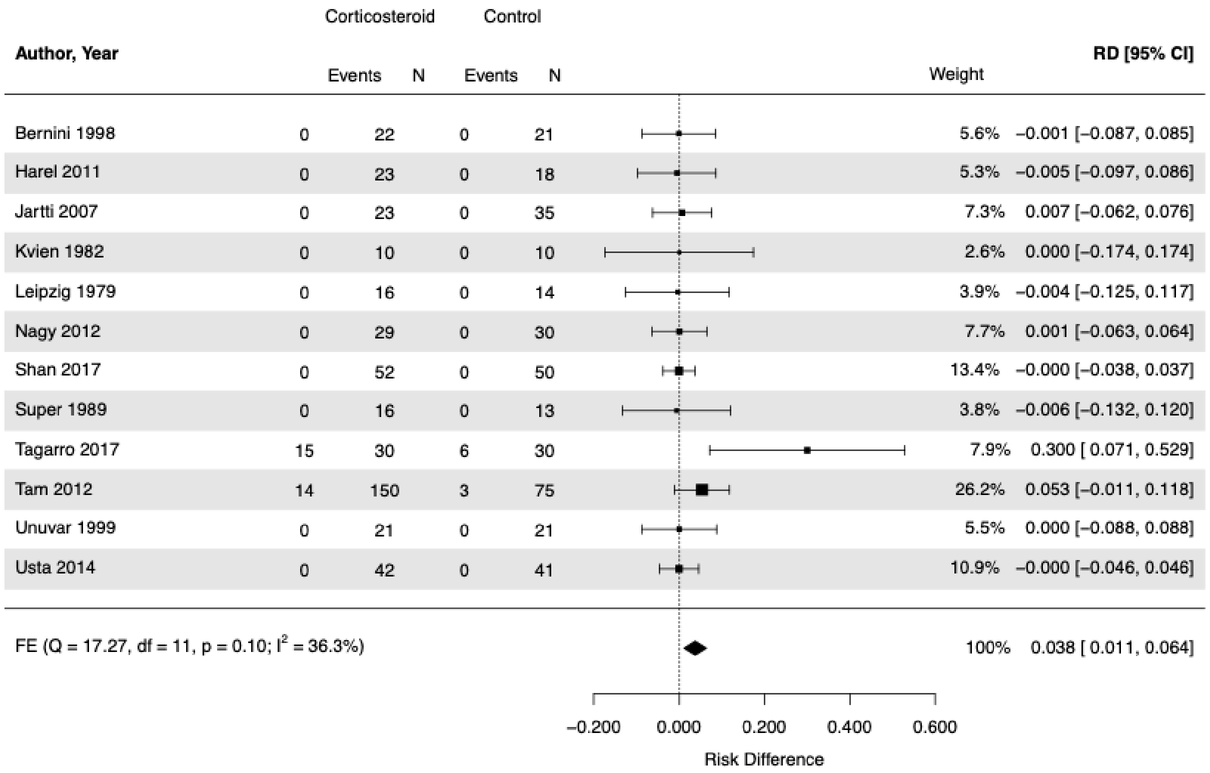

1.22 Forest plot – Hypertension

© 2025 Lima JP et al. *JAMA Network Open*.

Author, Year	Corticosteroid Events	Corticosteroid N	Control Events	Control N	Weight	RD [95% CI]
Bernini 1998	0	22	0	21	3.5%	−0.001 [−0.087, 0.085]
Dudley 2013	0	180	1	170	28.3%	−0.006 [−0.022, 0.010]
Griffin 1994	0	26	0	30	4.5%	0.002 [−0.065, 0.070]
Harel 2011	0	23	0	18	3.3%	−0.005 [−0.097, 0.086]
Jartti 2007	0	23	0	35	4.5%	0.007 [−0.062, 0.076]
Kvien 1982	0	10	0	10	1.6%	0.000 [−0.174, 0.174]
Leipzig 1979	0	16	0	14	2.4%	−0.004 [−0.125, 0.117]
Nagy 2012	0	29	0	30	4.8%	0.001 [−0.063, 0.064]
Ogata 2012	2	22	0	26	3.9%	0.090 [−0.047, 0.227]
Pierson 1974	0	30	0	15	3.2%	−0.015 [−0.111, 0.081]
Shan 2017	0	52	0	50	8.3%	−0.000 [−0.038, 0.037]
Sundel 2003	1	18	0	21	3.1%	0.056 [−0.080, 0.193]
Super 1989	0	16	0	13	2.3%	−0.006 [−0.132, 0.120]
Tam 2012	0	150	1	75	16.2%	−0.016 [−0.049, 0.016]
Unuvar 1999	0	21	0	21	3.4%	0.000 [−0.088, 0.088]
Usta 2014	0	42	0	41	6.7%	−0.000 [−0.046, 0.046]
FE (Q = 4.43, df = 15, p = 1.00; I^2 = 0.0%)					100%	0.001 [−0.006, 0.009]

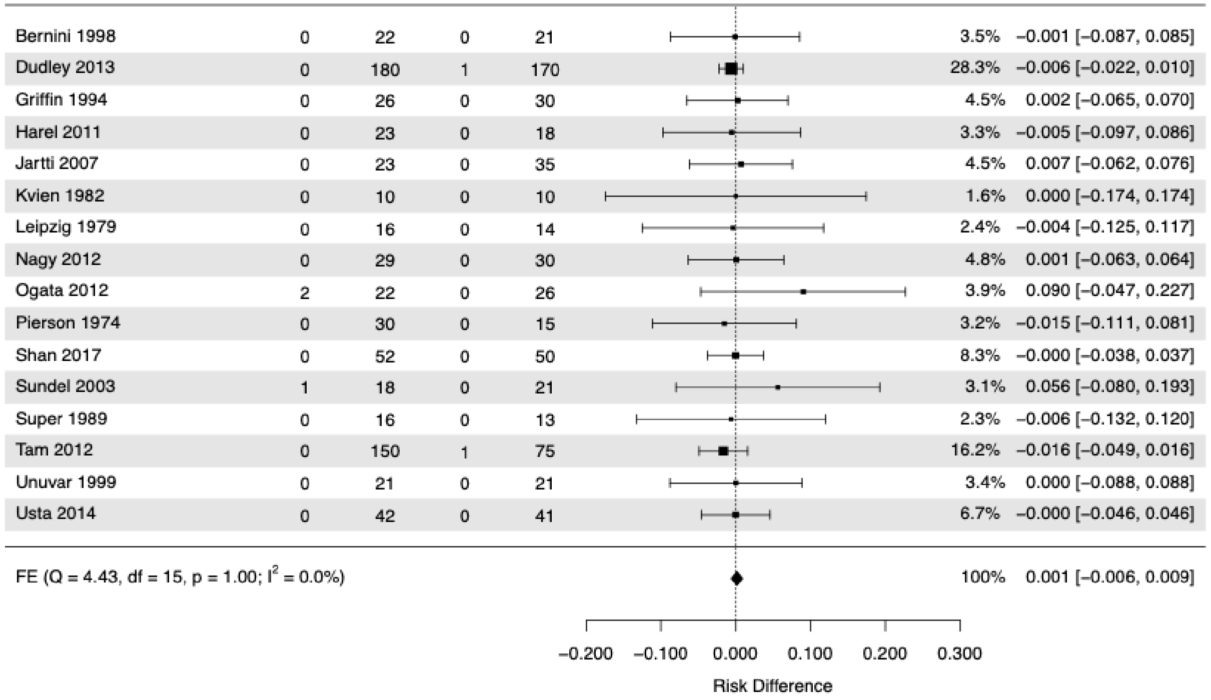

1.23 Forest plot – Increased Appetite

© 2025 Lima JP et al. *JAMA Network Open*.

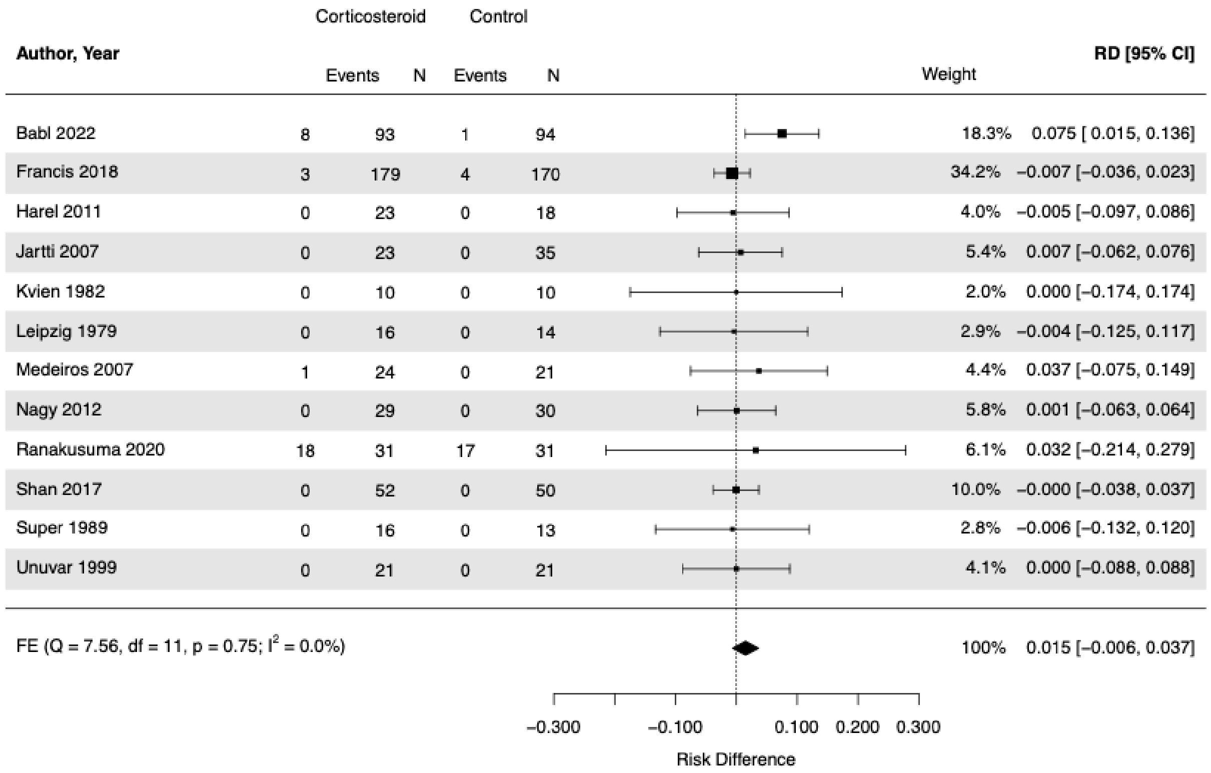

1.24 Forest plot – Irritability

© 2025 Lima JP et al. *JAMA Network Open*.

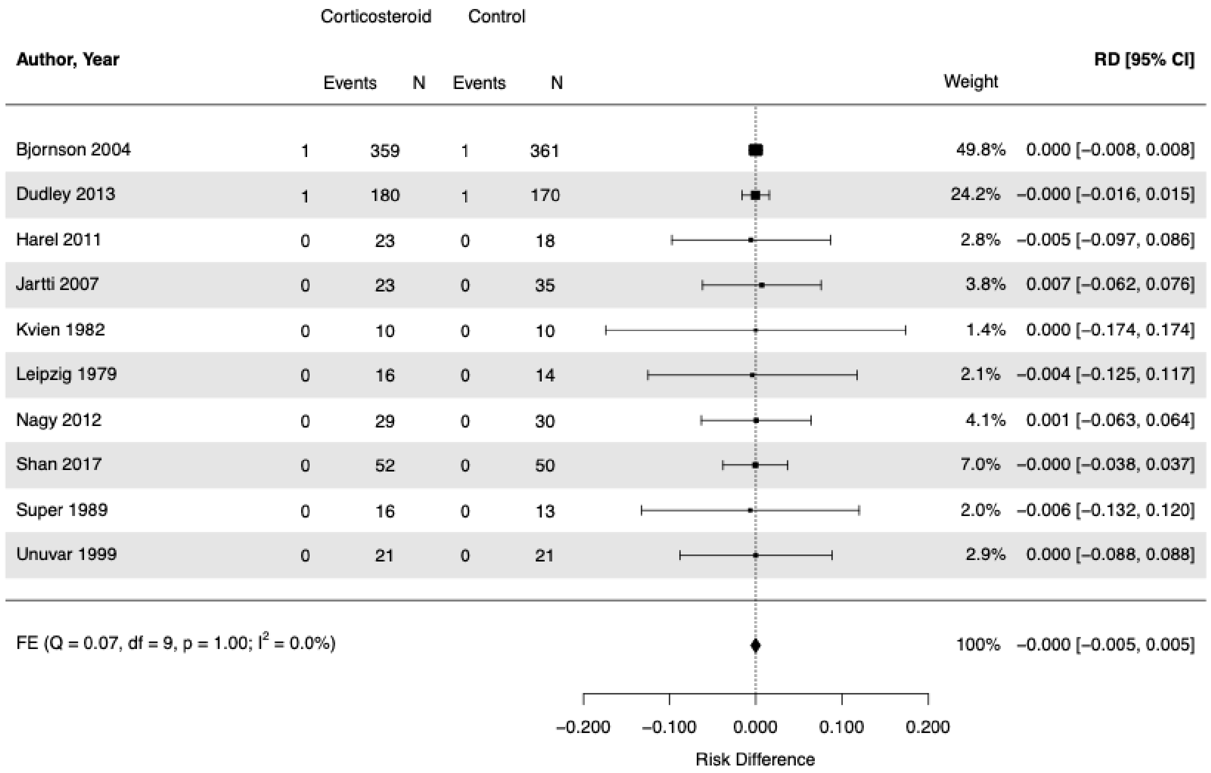

1.25 Forest plot – Local Site Pain

© 2025 Lima JP et al. *JAMA Network Open*.

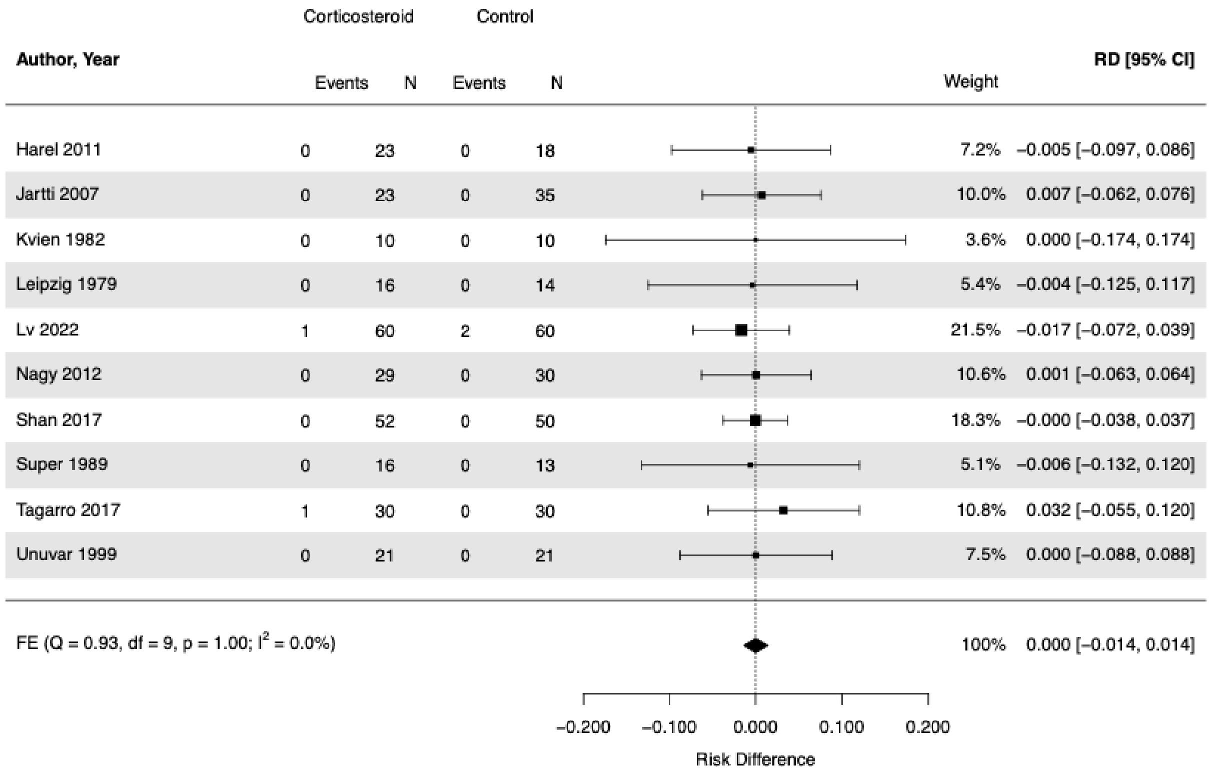

1.26 Forest plot – Musculoskeletal Pain

© 2025 Lima JP et al. *JAMA Network Open*.

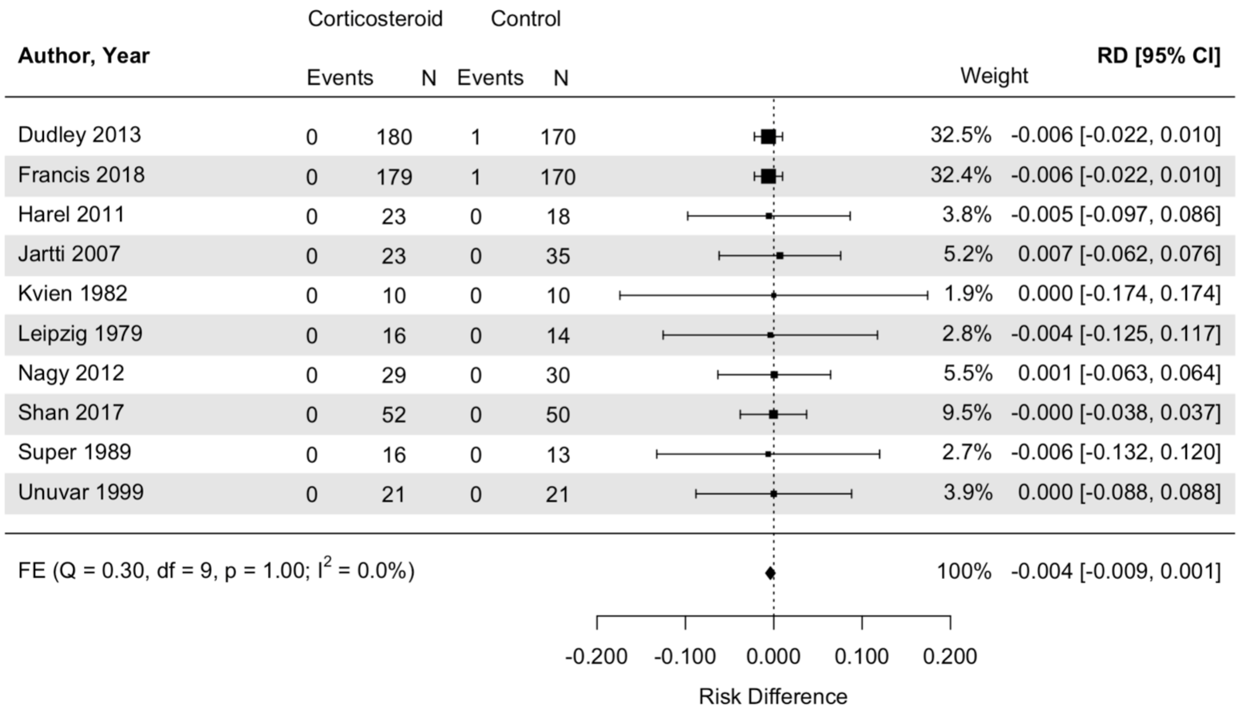

1.26 Forest plot – Myalgia

© 2025 Lima JP et al. *JAMA Network Open*.

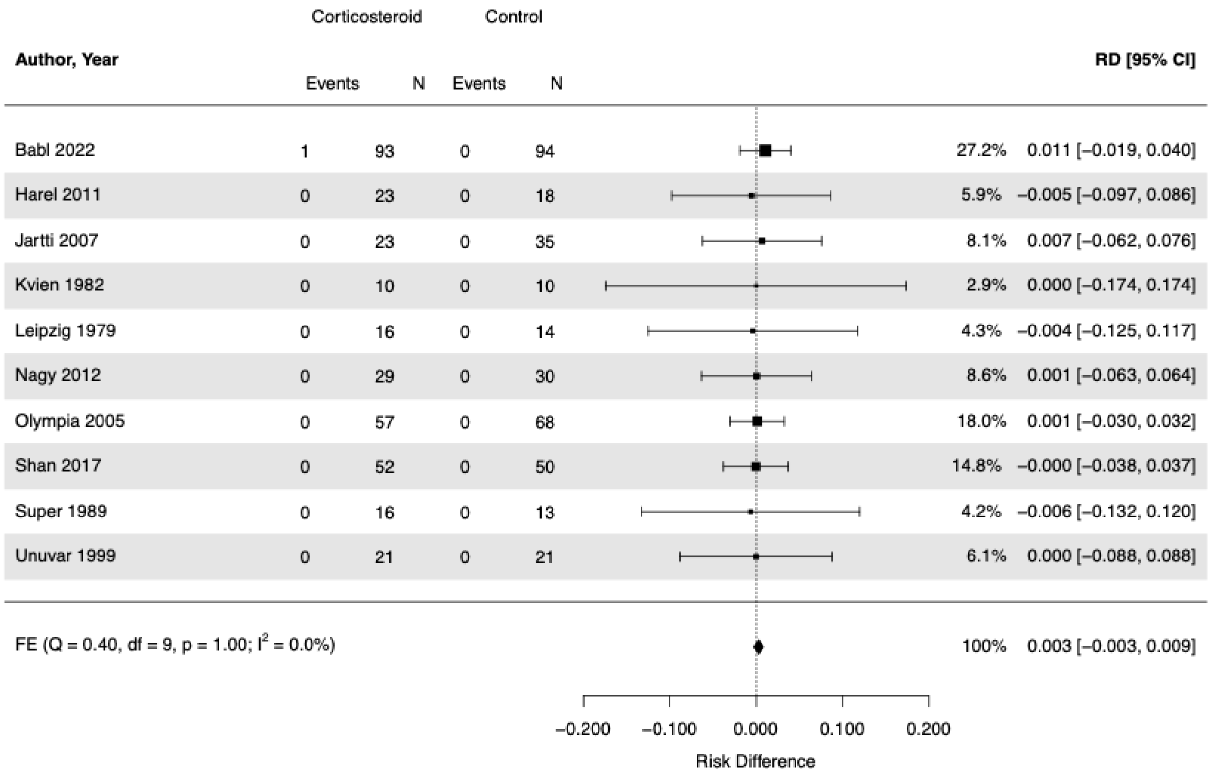

1.27 Forest plot – Otitis Media

© 2025 Lima JP et al. *JAMA Network Open*.

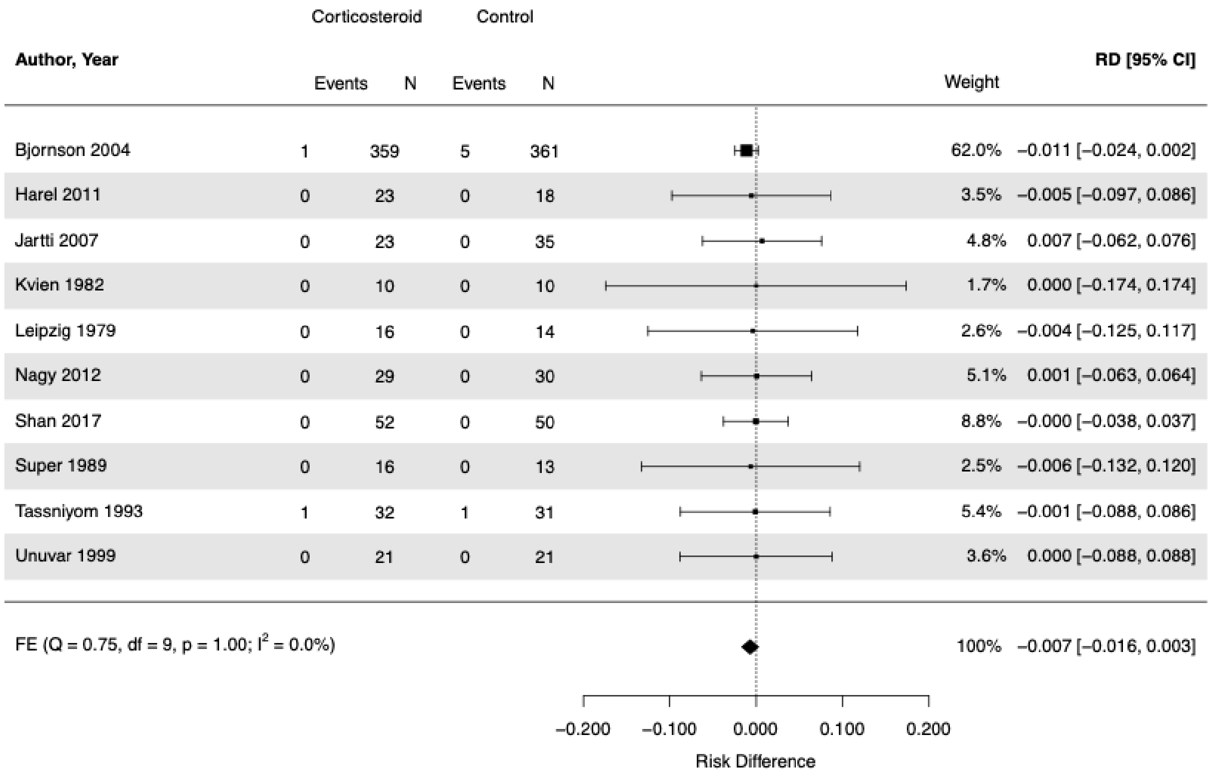

1.28 Forest plot – Pneumonia

© 2025 Lima JP et al. *JAMA Network Open*.

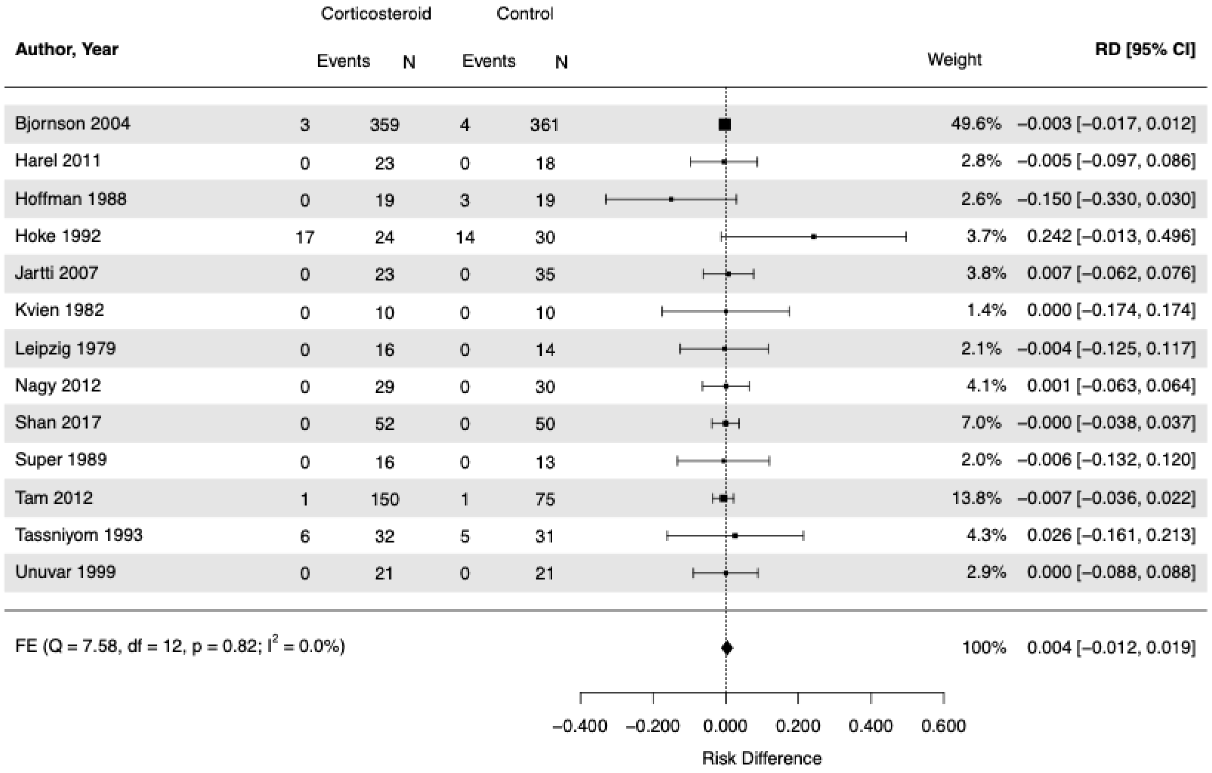

1.29 Forest plot – Polyuria

© 2025 Lima JP et al. *JAMA Network Open*.

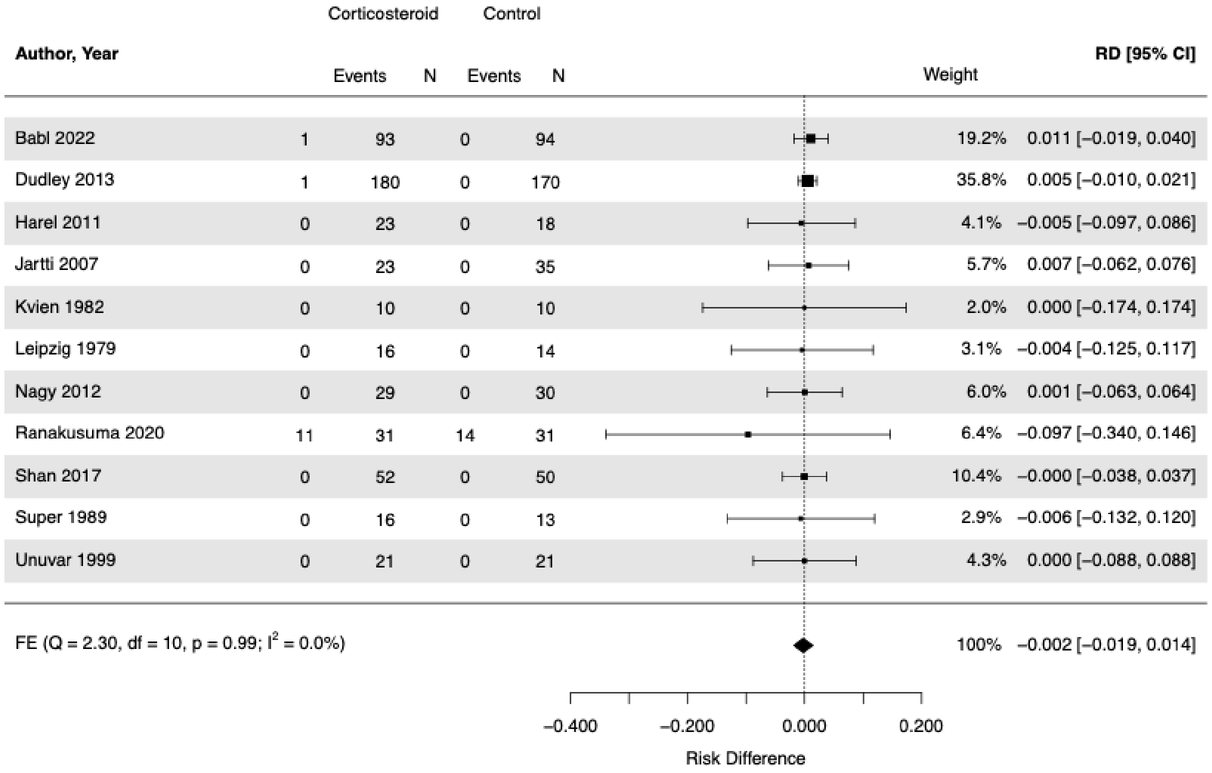

1.30 Forest plot – Rash or Urticaria

© 2025 Lima JP et al. *JAMA Network Open*.

Author, Year	Corticosteroid Events	Corticosteroid N	Control Events	Control N	Weight	RD [95% CI]
Babl 2022	1	93	4	94	6.1%	−0.032 [−0.078, 0.014]
Bjornson 2004	2	359	0	361	23.5%	0.006 [−0.004, 0.015]
Csonka 2003	2	113	0	117	7.5%	0.018 [−0.012, 0.047]
Foster 2018	0	308	1	304	19.9%	−0.003 [−0.012, 0.006]
Francis 2018	0	179	2	170	11.4%	−0.012 [−0.031, 0.008]
Harel 2011	0	23	0	18	1.3%	−0.005 [−0.097, 0.086]
Huber 2004	0	21	1	19	1.3%	−0.052 [−0.183, 0.079]
Jartti 2007	0	23	0	35	1.8%	0.007 [−0.062, 0.076]
Kvien 1982	0	10	0	10	0.7%	0.000 [−0.174, 0.174]
Leipzig 1979	0	16	0	14	1.0%	−0.004 [−0.125, 0.117]
Lv 2022	1	60	1	60	3.9%	0.000 [−0.046, 0.046]
Nagy 2012	0	29	0	30	1.9%	0.001 [−0.063, 0.064]
Prakash 2006	0	25	1	27	1.7%	−0.034 [−0.133, 0.064]
Ranakusuma 2020	3	31	0	31	2.0%	0.094 [−0.023, 0.210]
Shan 2017	0	52	0	50	3.3%	−0.000 [−0.038, 0.037]
Super 1989	0	16	0	13	0.9%	−0.006 [−0.132, 0.120]
Tagarro 2017	0	30	1	30	2.0%	−0.032 [−0.120, 0.055]
Tam 2012	0	150	1	75	6.5%	−0.016 [−0.049, 0.016]
Unuvar 1999	0	21	0	21	1.4%	0.000 [−0.088, 0.088]
Wang 2022	1	30	2	30	2.0%	−0.033 [−0.143, 0.077]
FE (Q = 12.42, df = 19, p = 0.87; I^2 = 0.0%)					100%	−0.003 [−0.009, 0.003]

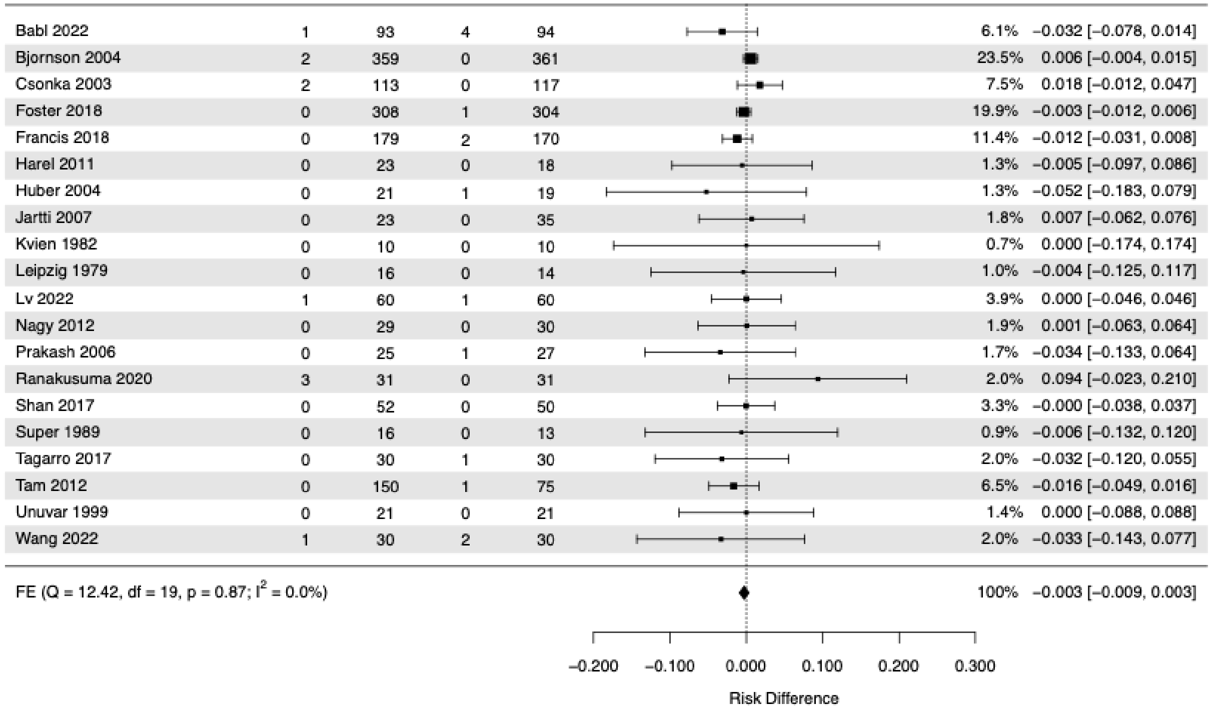

1.31 Forest plot – Secondary (Opportunistic) Infection

© 2025 Lima JP et al. *JAMA Network Open*.

Author, Year	Corticosteroid Events	Corticosteroid N	Control Events	Control N	Weight	RD [95% CI]
Bernini 1998	0	22	0	21	4.6%	−0.001 [−0.087, 0.085]
Dudley 2013	6	180	3	170	37.8%	0.016 [−0.017, 0.049]
Harel 2011	0	23	0	18	4.4%	−0.005 [−0.097, 0.086]
Jartti 2007	0	23	0	35	6.0%	0.007 [−0.062, 0.076]
Kuusela 1988	3	35	2	37	7.8%	0.032 [−0.086, 0.150]
Kvien 1982	0	10	0	10	2.2%	0.000 [−0.174, 0.174]
Leipzig 1979	0	16	0	14	3.2%	−0.004 [−0.125, 0.117]
Nagy 2012	0	29	0	30	6.4%	0.001 [−0.063, 0.064]
Shan 2017	0	52	0	50	11.0%	−0.000 [−0.038, 0.037]
Super 1989	0	16	0	13	3.1%	−0.006 [−0.132, 0.120]
Unuvar 1999	0	21	0	21	4.5%	0.000 [−0.088, 0.088]
Usta 2014	0	42	0	41	9.0%	−0.000 [−0.046, 0.046]
FE (Q = 1.01, df = 11, p = 1.00; I^2 = 0.0%)					100%	0.008 [−0.007, 0.024]

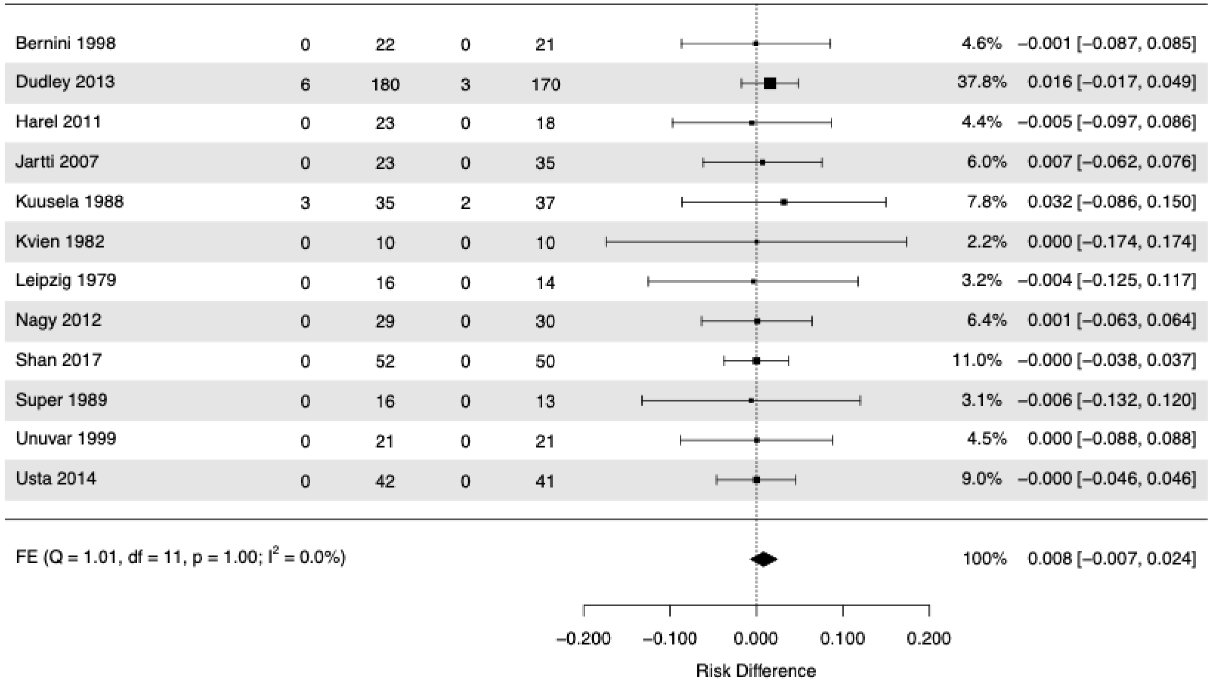

1.32 Forest plot – Secondary Fever

© 2025 Lima JP et al. *JAMA Network Open*.

Author, Year	Corticosteroid Events	Corticosteroid N	Control Events	Control N	Weight	RD [95% CI]
Bjornson 2004	5	359	4	361	51.0%	0.003 [−0.013, 0.019]
Harel 2011	0	23	0	18	2.9%	−0.005 [−0.097, 0.086]
Jartti 2007	0	23	0	35	3.9%	0.007 [−0.062, 0.076]
Kvien 1982	0	10	0	10	1.4%	0.000 [−0.174, 0.174]
Leipzig 1979	0	16	0	14	2.1%	−0.004 [−0.125, 0.117]
Nagy 2012	0	29	0	30	4.2%	0.001 [−0.063, 0.064]
Ogata 2012	5	22	0	26	3.4%	0.221 [0.039, 0.402]
Qazi 1996	20	48	14	41	6.3%	0.075 [−0.126, 0.277]
Schaad 1993	19	60	11	55	8.1%	0.117 [−0.042, 0.275]
Shan 2017	0	52	0	50	7.2%	−0.000 [−0.038, 0.037]
Super 1989	0	16	0	13	2.0%	−0.006 [−0.132, 0.120]
Tassniyom 1993	16	32	15	31	4.5%	0.016 [−0.231, 0.263]
Unuvar 1999	0	21	0	21	3.0%	0.000 [−0.088, 0.088]
FE (Q = 16.19, df = 12, p = 0.18; I^2 = 25.9%)					100%	0.024 [0.000, 0.048]

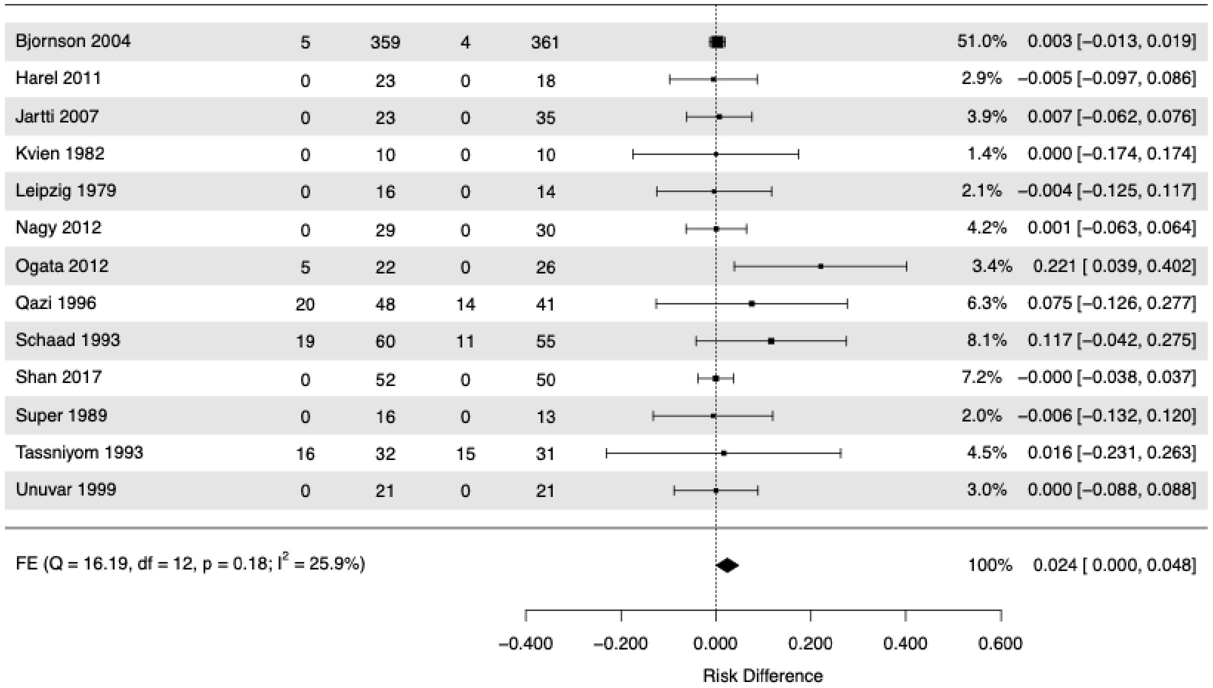

r

1.33 Forest plot – Sleep Problems

© 2025 Lima JP et al. *JAMA Network Open*.

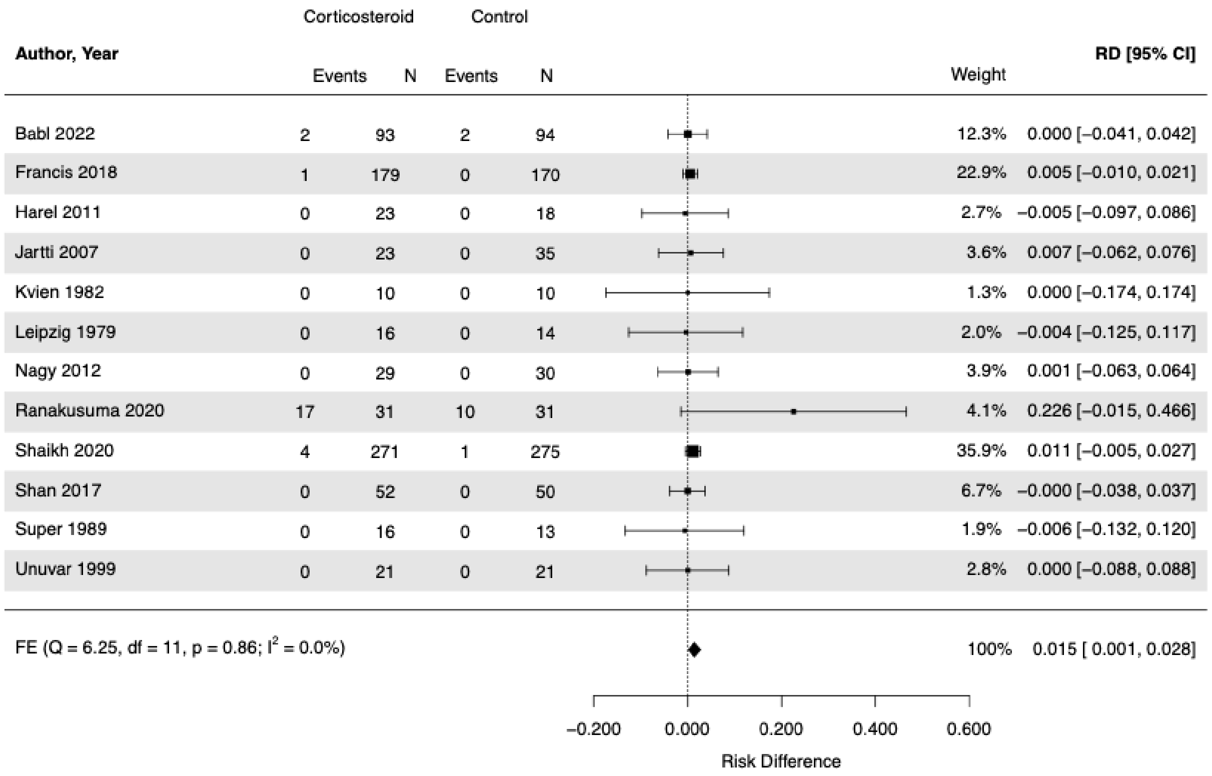

1.34 Forest plot – Tremor or Hyperactivity

© 2025 Lima JP et al. *JAMA Network Open*.

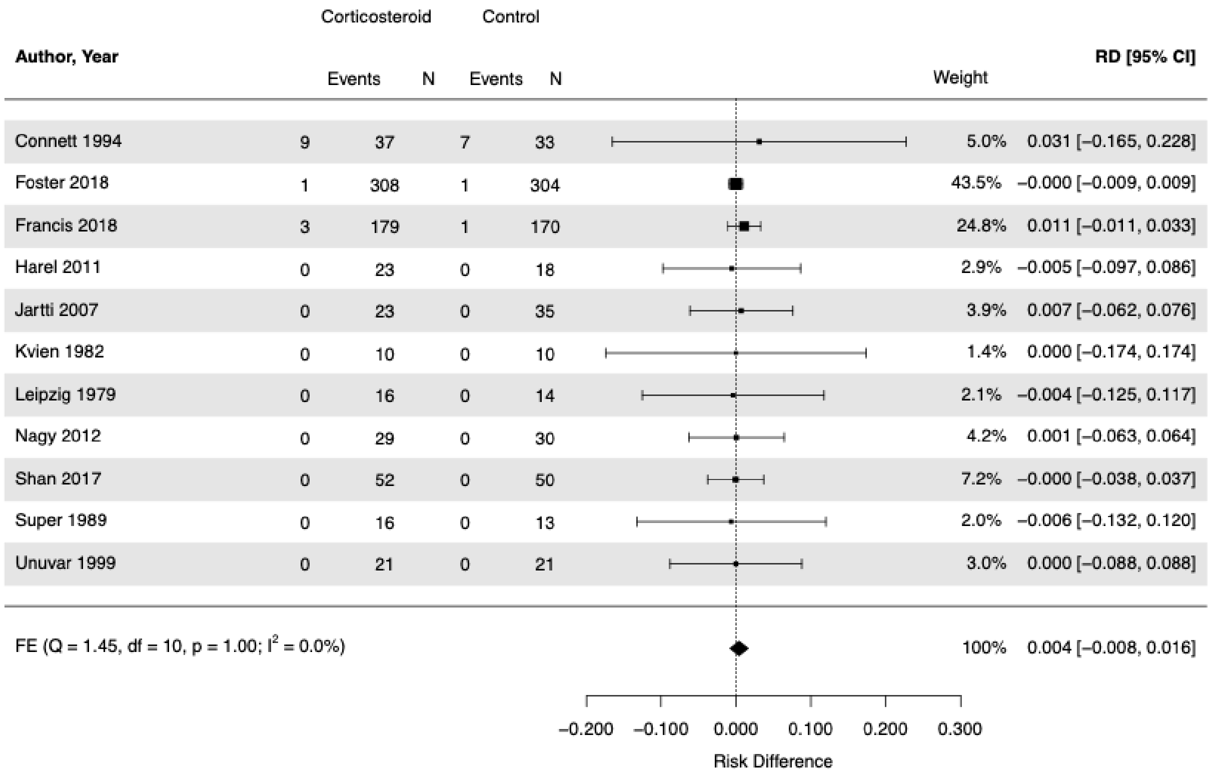

1.35 Forest plot – Urinary Tract Infection

© 2025 Lima JP et al. *JAMA Network Open*.

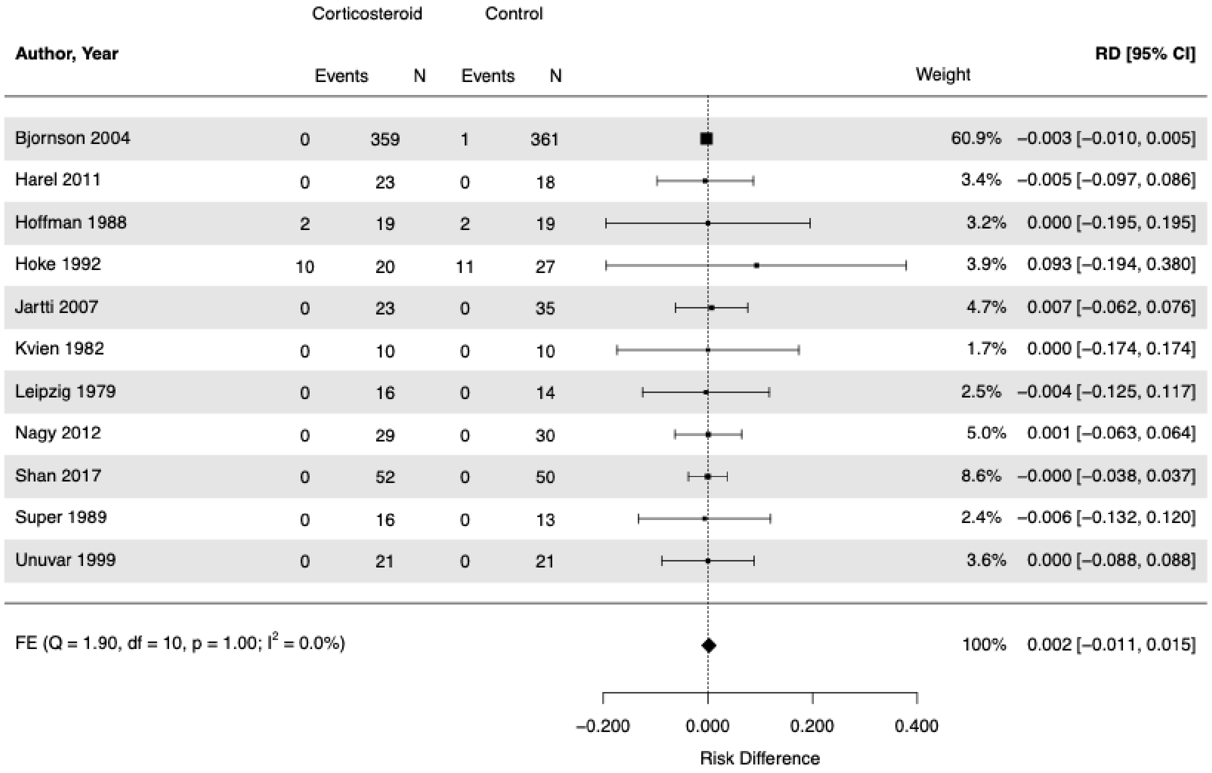

eFigure2: Funnel Plots

2.1 Funnel plot – Serious Adverse Events

© 2025 Lima JP et al. *JAMA Network Open*.

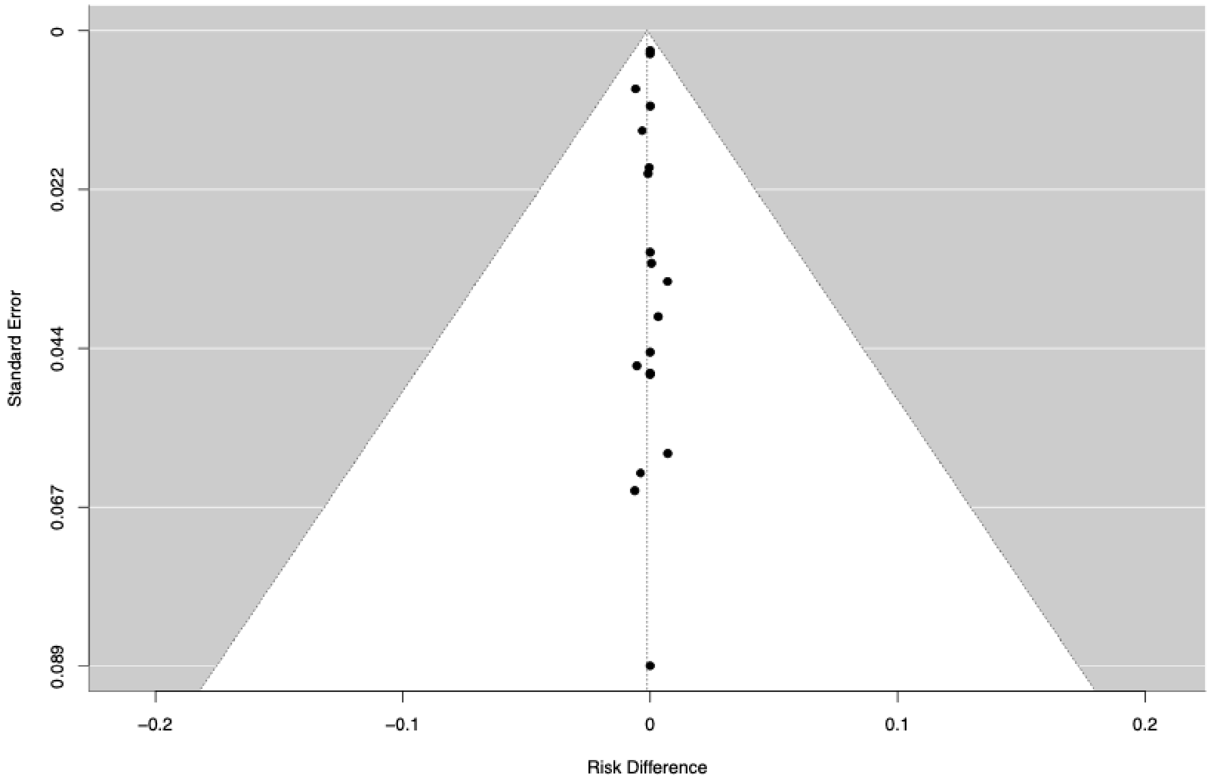

© 2025 Lima JP et al. *JAMA Network Open*.

2.2 Funnel plot – Adverse Events Leading to Discontinuation

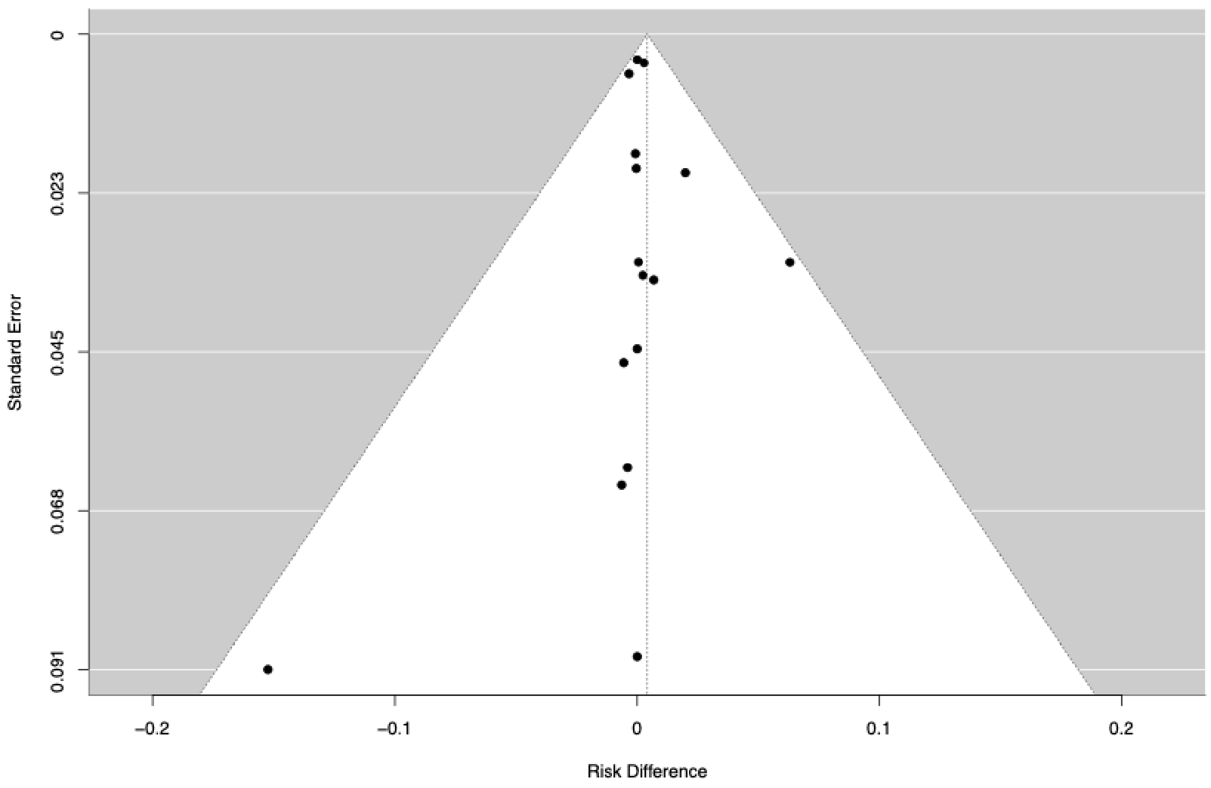

2.3 Funnel plot – Abdominal Pain

© 2025 Lima JP et al. *JAMA Network Open*.

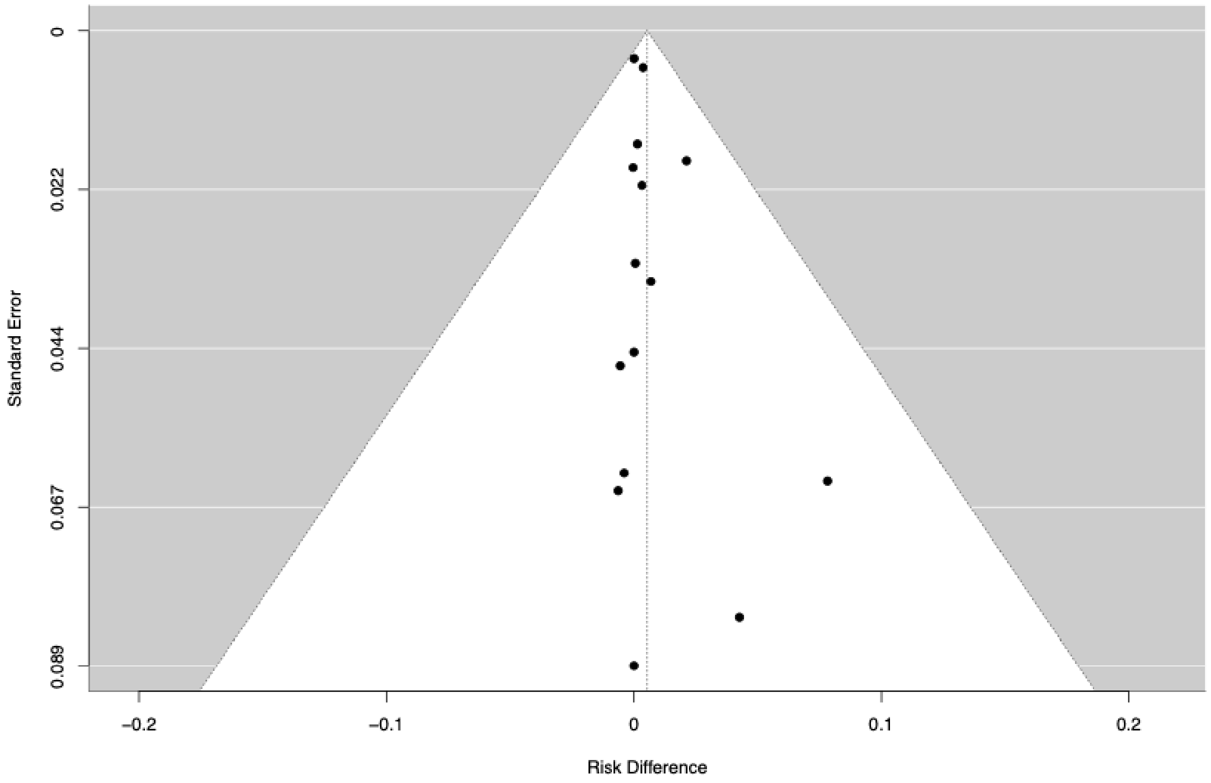

2.4 Funnel plot – Diarrhea

© 2025 Lima JP et al. *JAMA Network Open*.

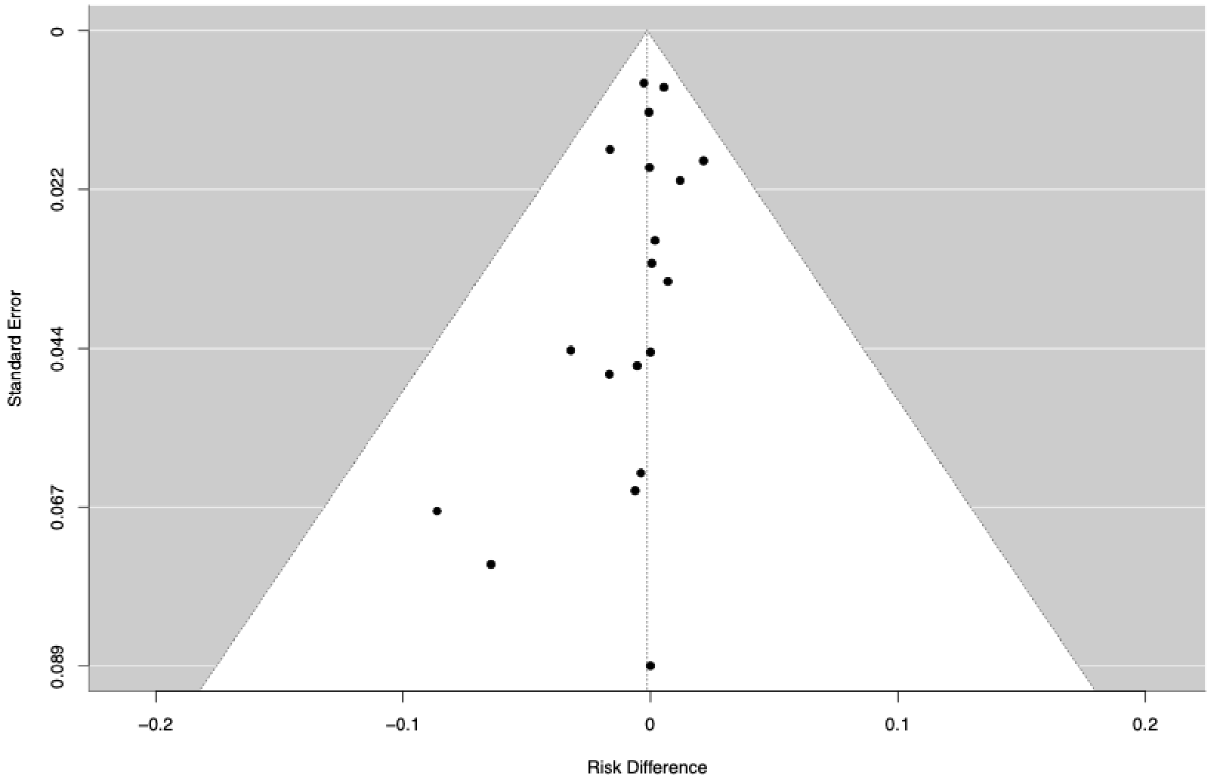

2.5 Funnel plot – Gastritis

© 2025 Lima JP et al. *JAMA Network Open*.

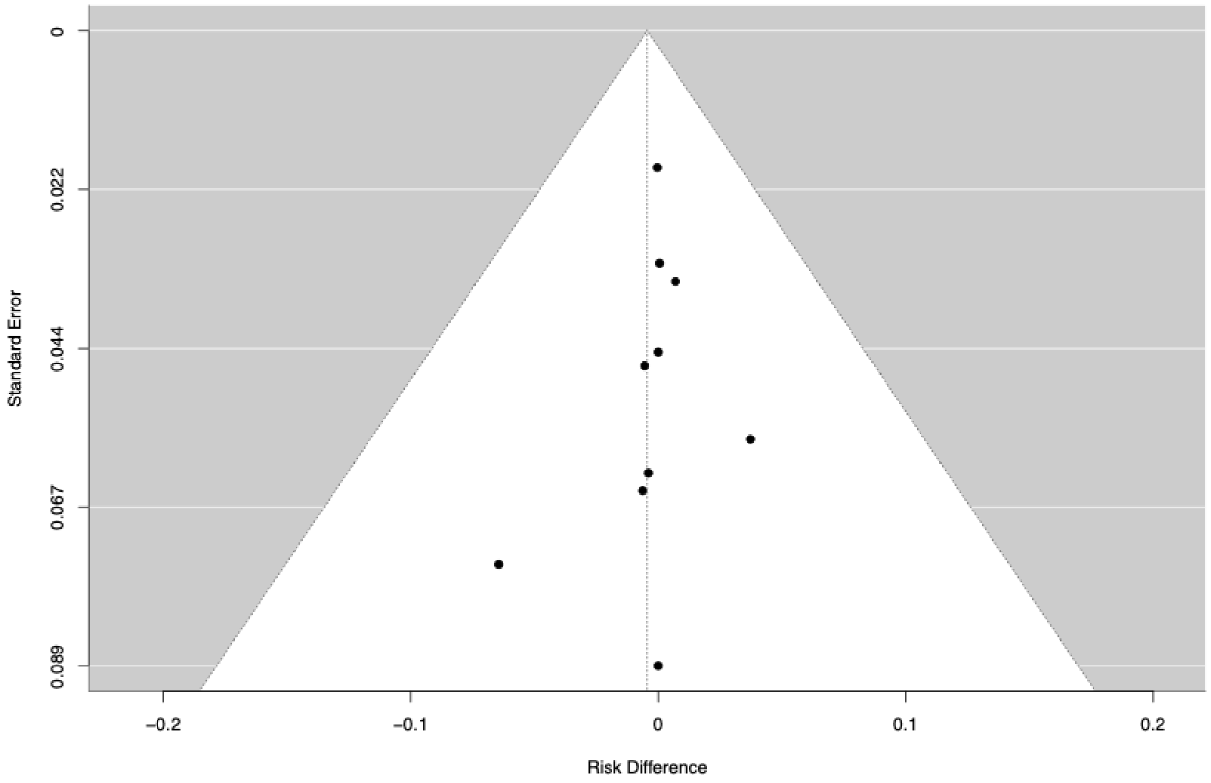

2.6 Funnel plot – Gastrointestinal Bleeding

© 2025 Lima JP et al. *JAMA Network Open*.

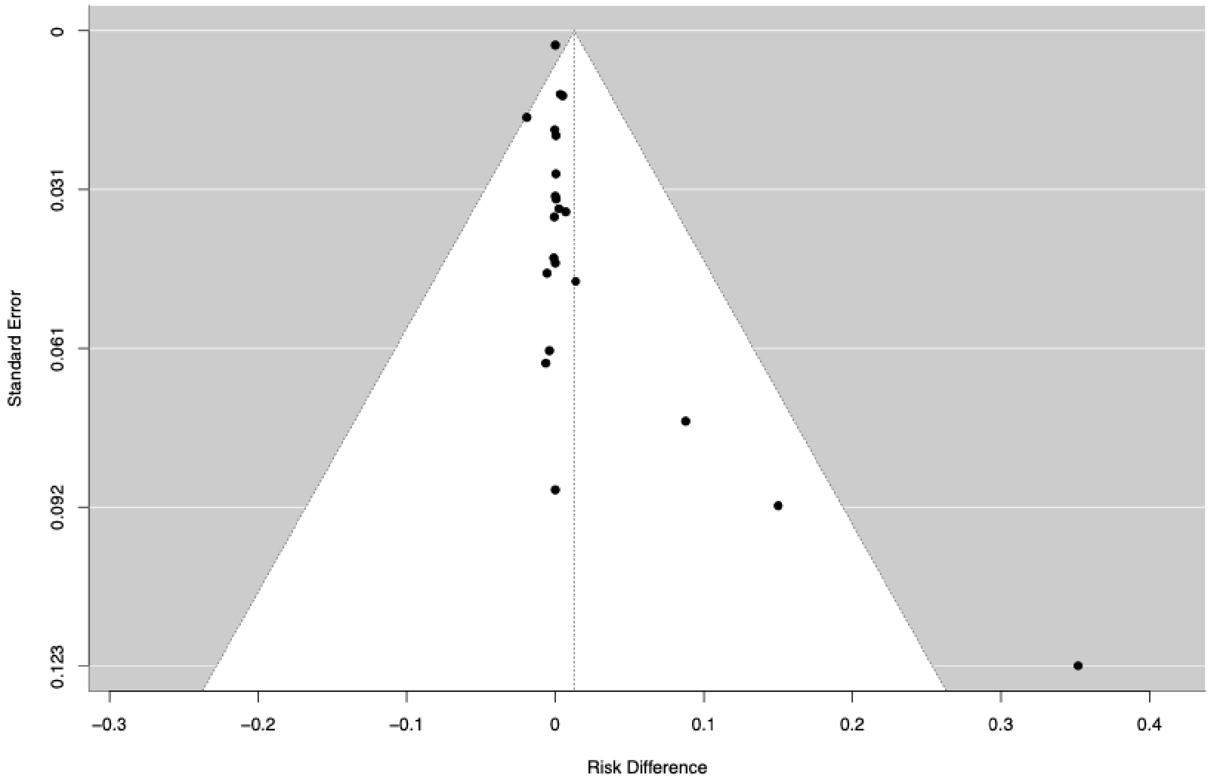

2.7 Funnel plot – Hemoccult Positive Stool

© 2025 Lima JP et al. *JAMA Network Open*.

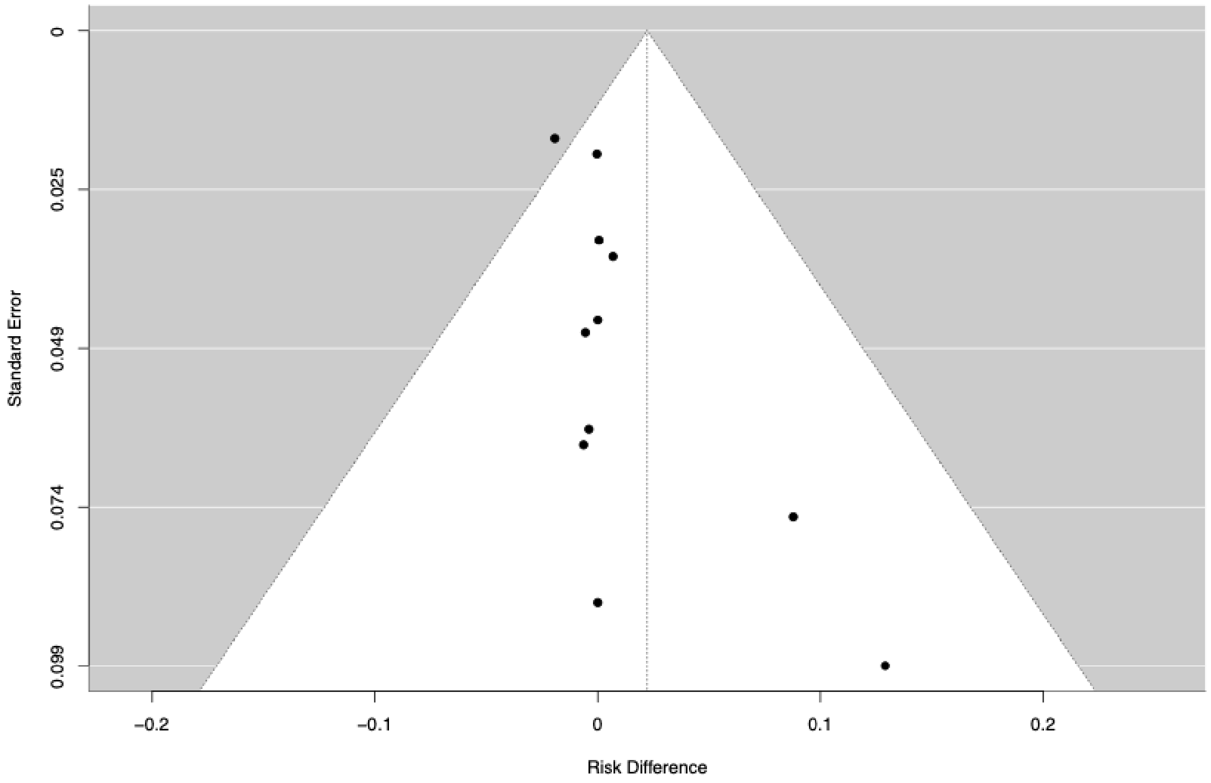

2.8 Funnel plot – Intussusception

© 2025 Lima JP et al. *JAMA Network Open*.

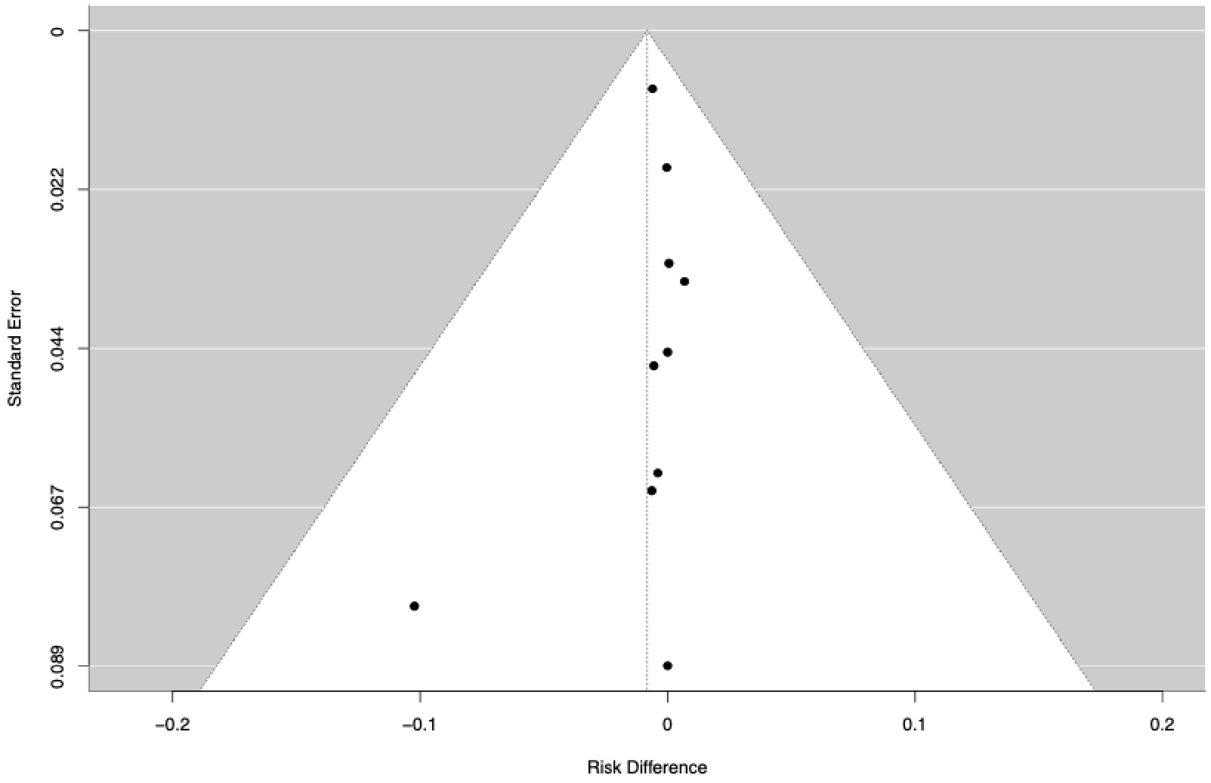

2.9 Funnel plot – Nausea

© 2025 Lima JP et al. *JAMA Network Open*.

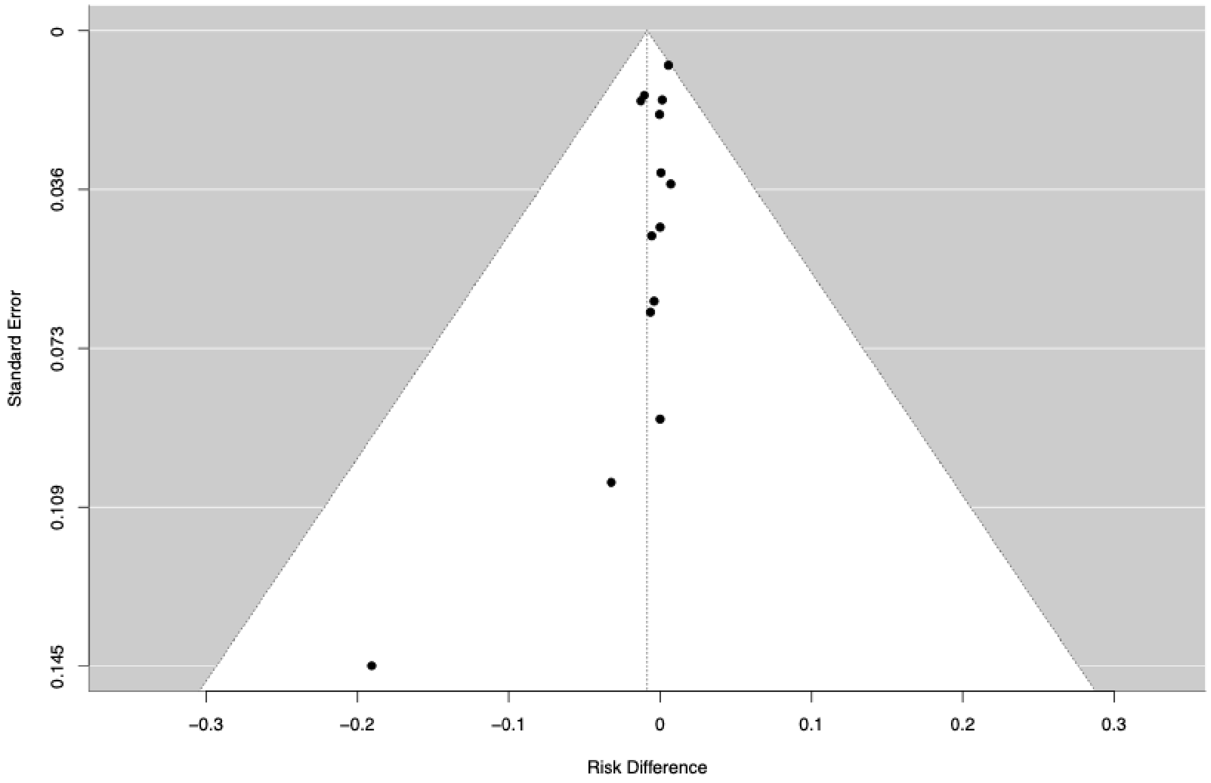

2.10 Funnel plot – Vomiting

© 2025 Lima JP et al. *JAMA Network Open*.

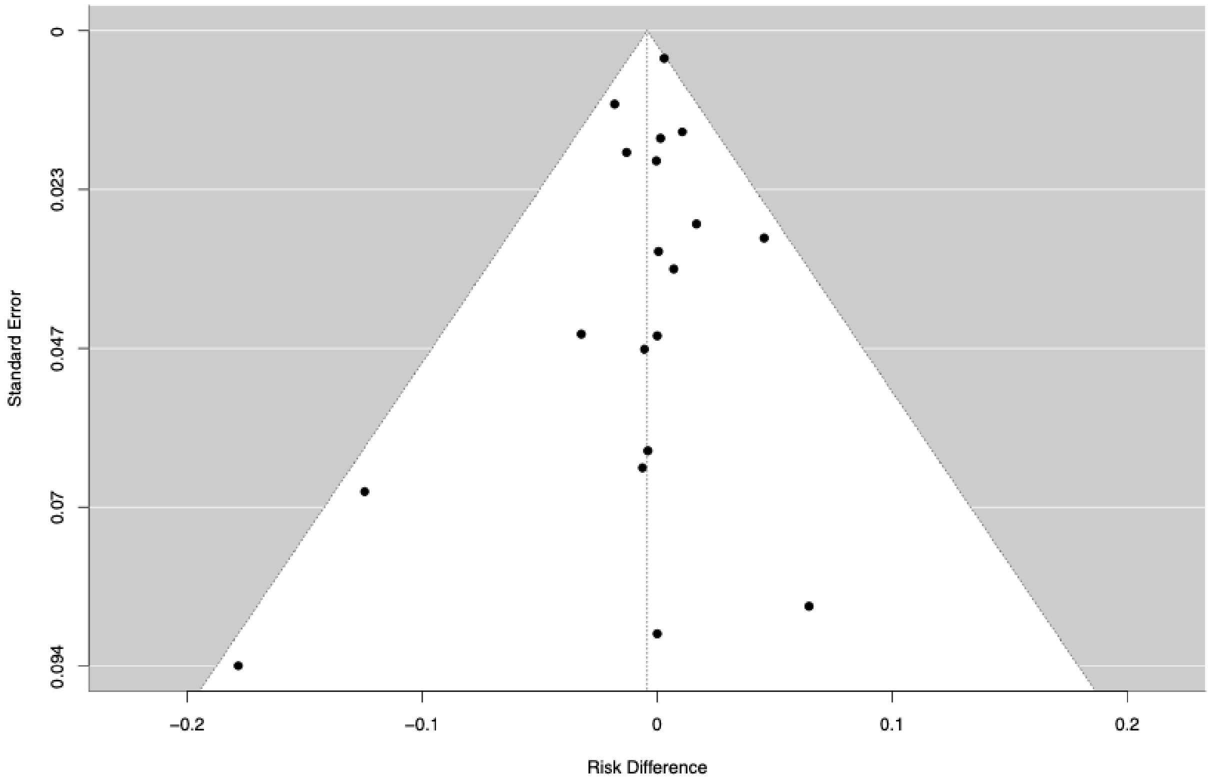

2.11 Funnel plot – Anemia

© 2025 Lima JP et al. *JAMA Network Open*.

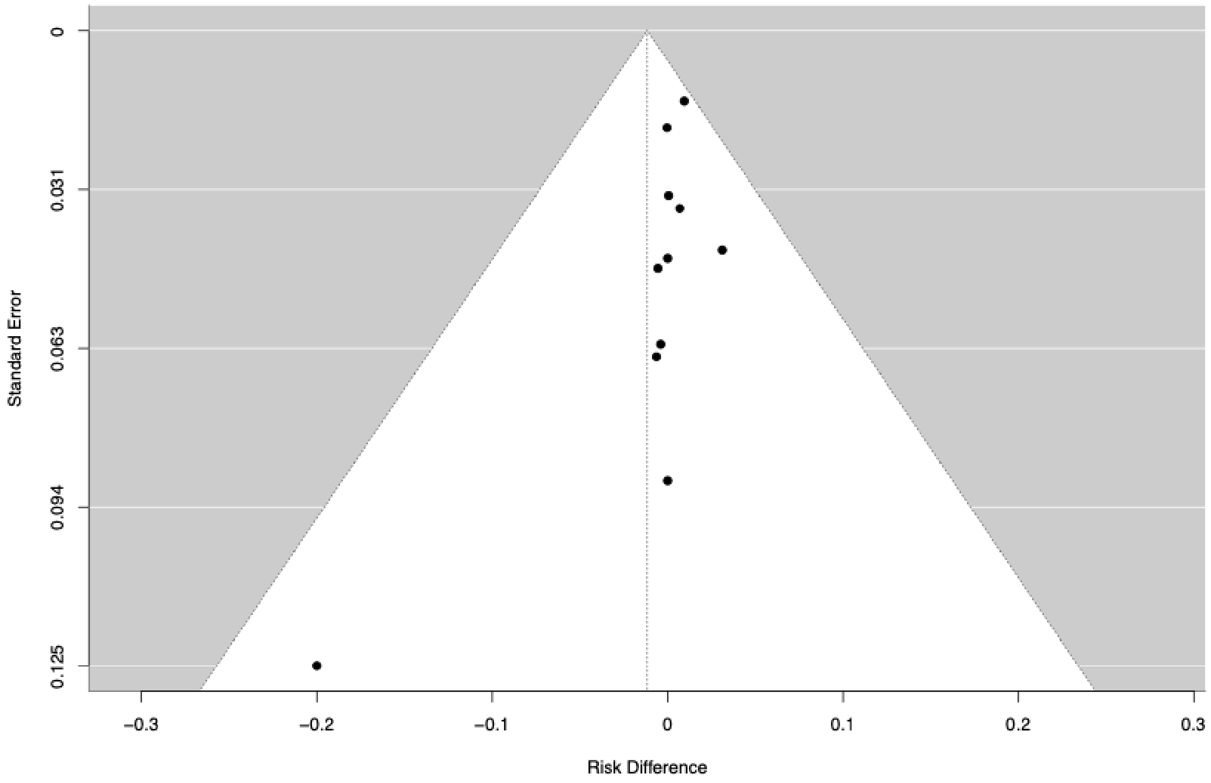

2.12 Funnel plot – Candidiasis

© 2025 Lima JP et al. *JAMA Network Open*.

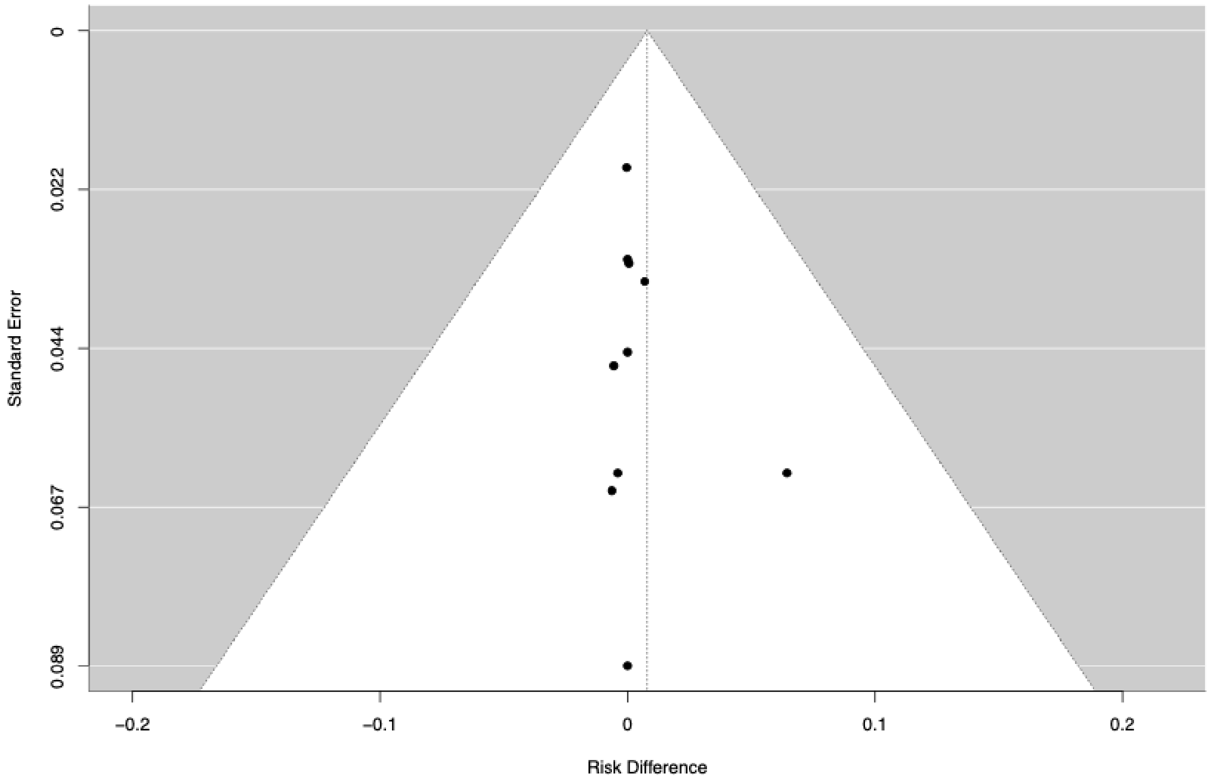

2.13 Funnel plot – Change in behaviour

© 2025 Lima JP et al. *JAMA Network Open*.

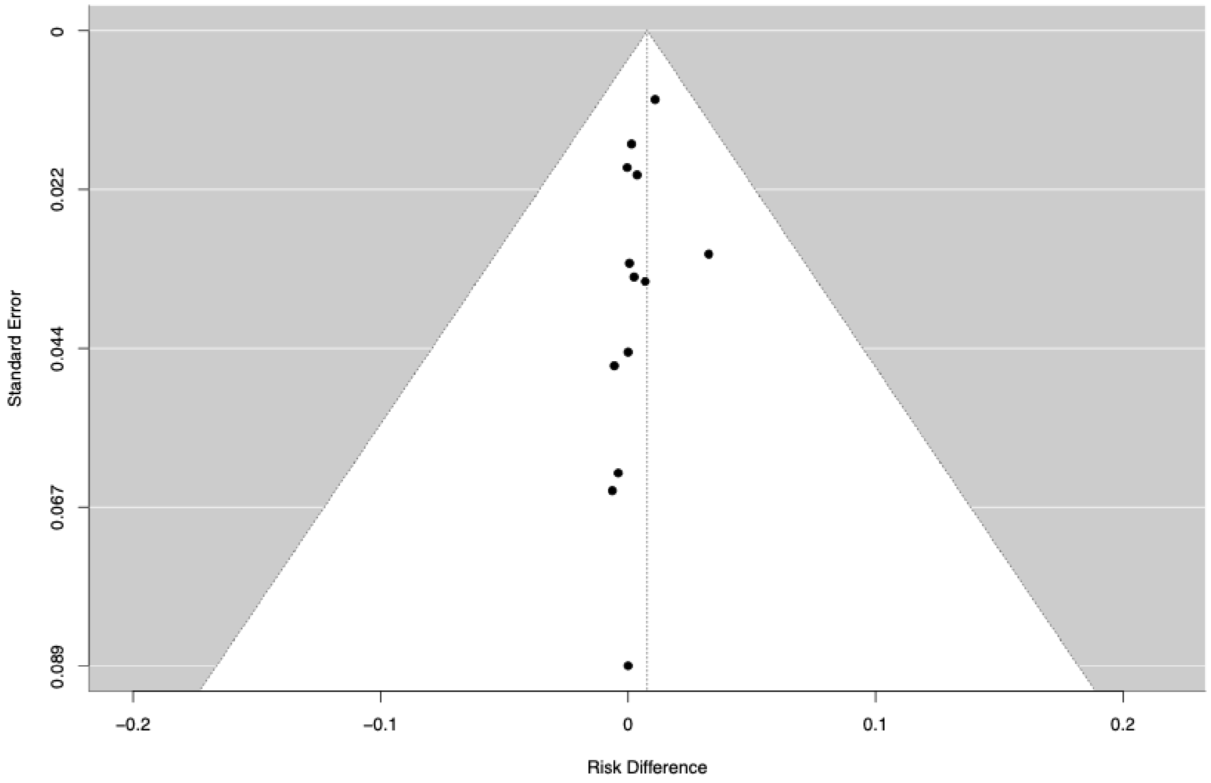

2.14 Funnel plot – Congestive Heart Failure

© 2025 Lima JP et al. *JAMA Network Open*.

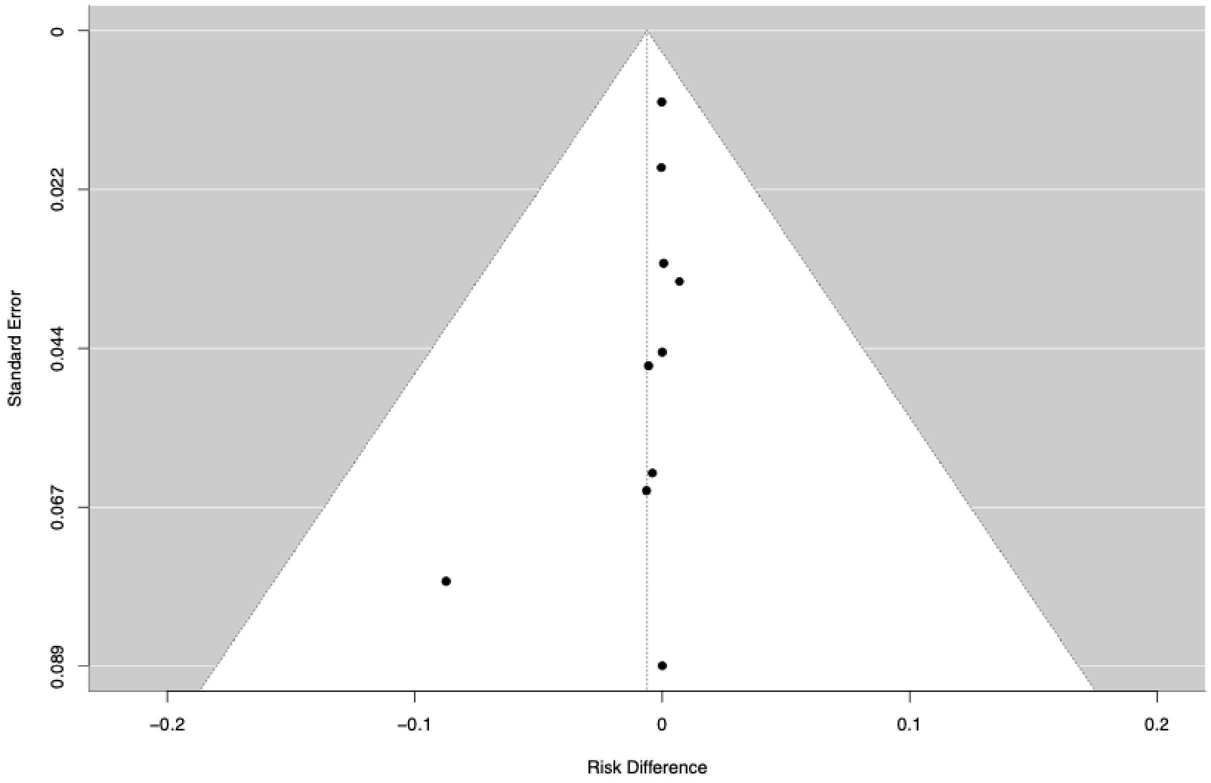

2.15 Funnel plot – Convulsion/Seizure

© 2025 Lima JP et al. *JAMA Network Open*.

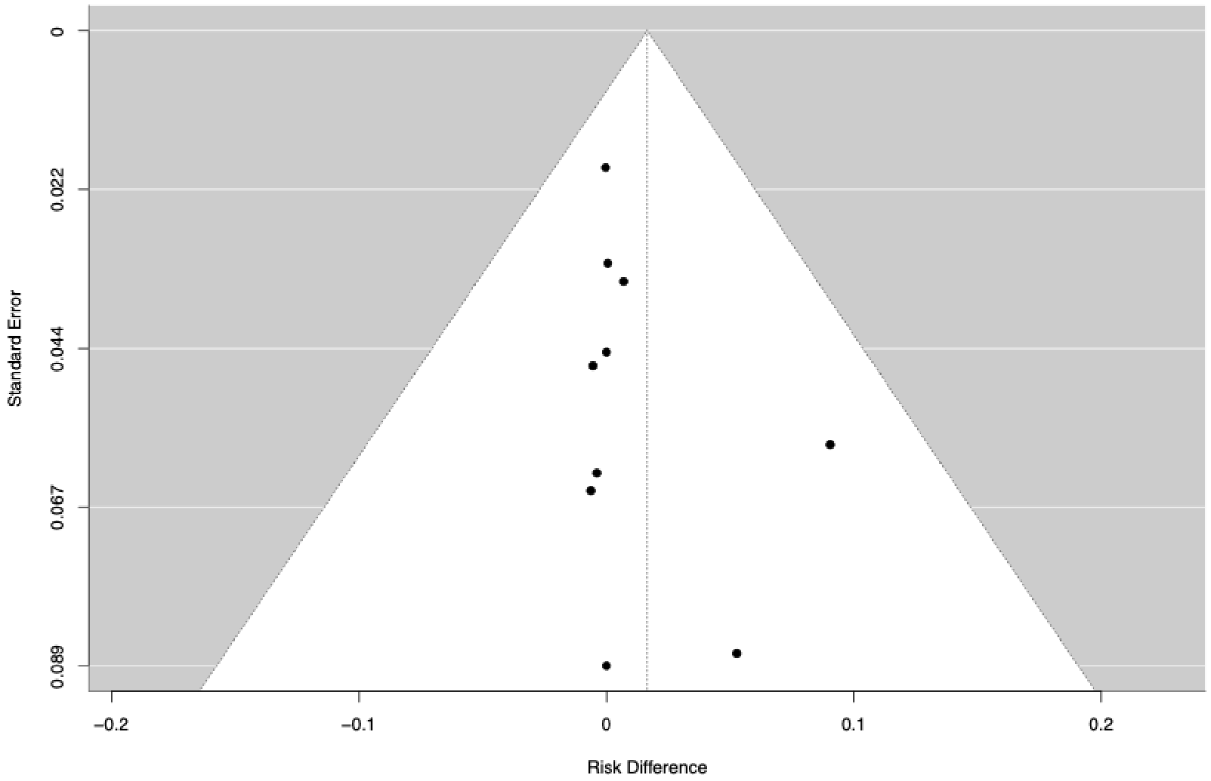

2.16 Funnel plot – Decreased Appetite

© 2025 Lima JP et al. *JAMA Network Open*.

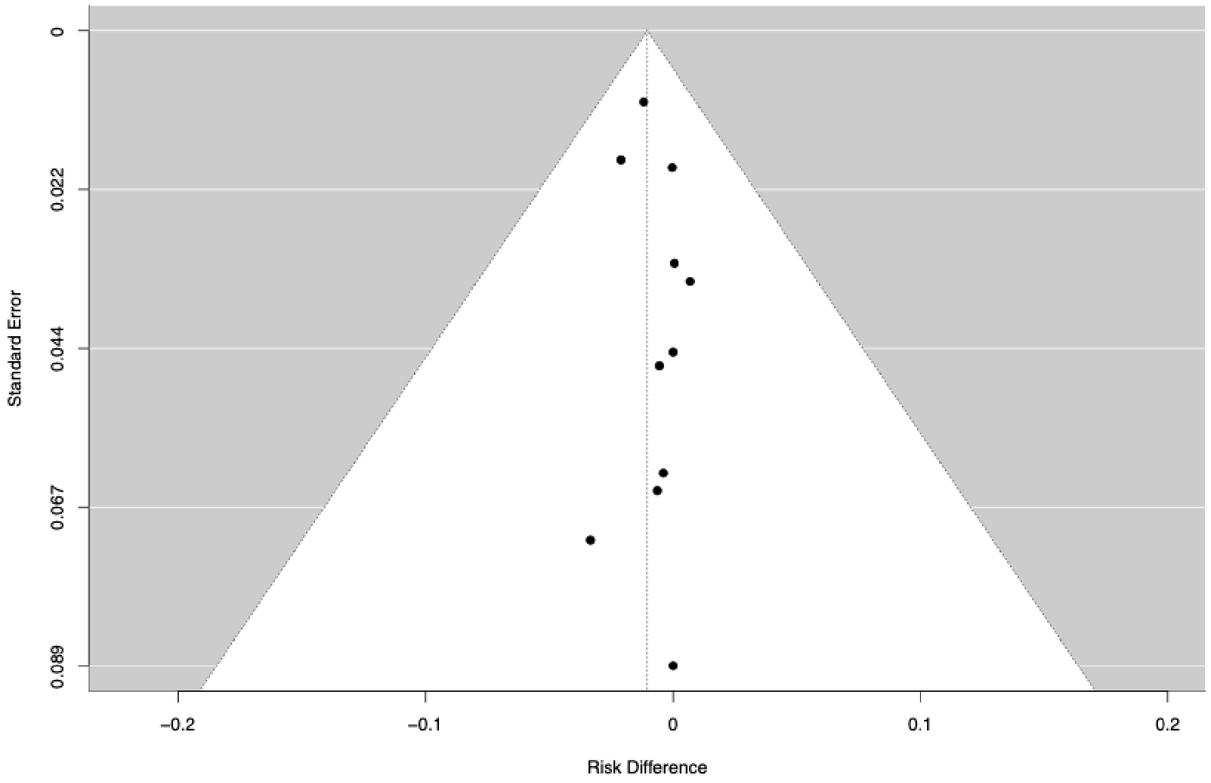

2.17 Funnel plot – Dizziness

© 2025 Lima JP et al. *JAMA Network Open*.

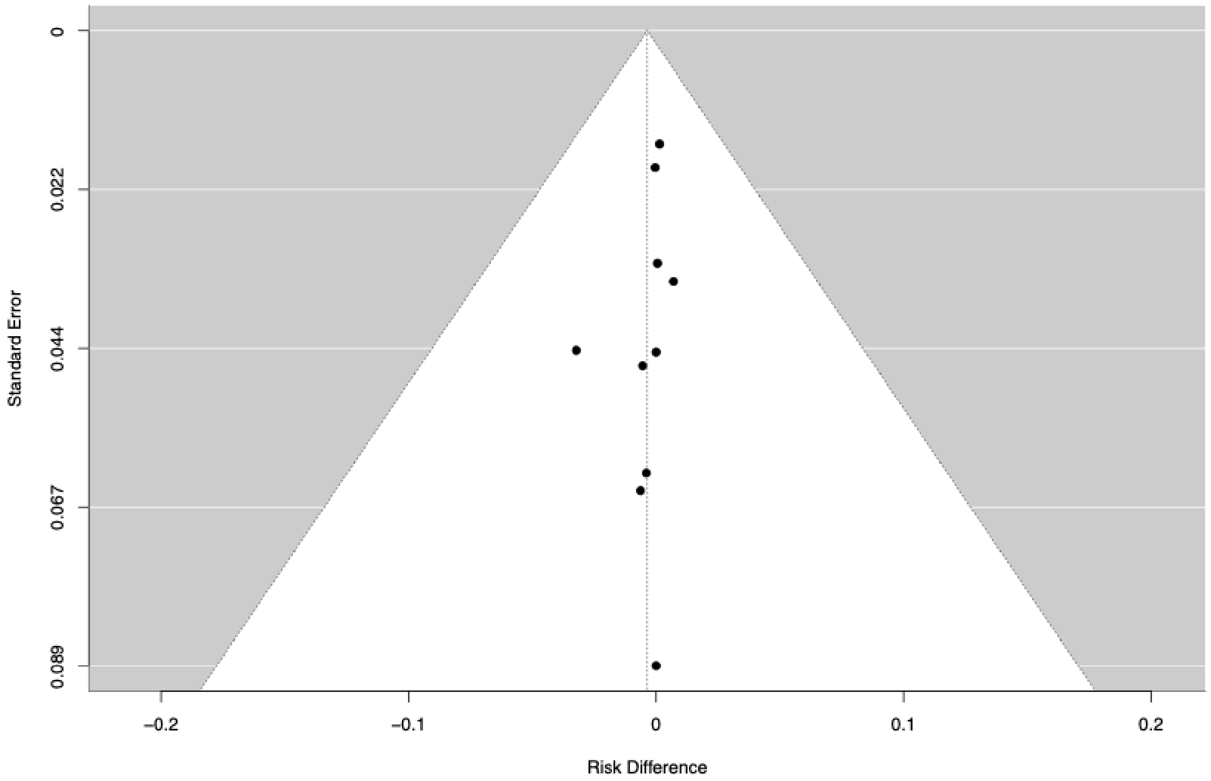

2.18 Funnel plot – Fatigue

© 2025 Lima JP et al. *JAMA Network Open*.

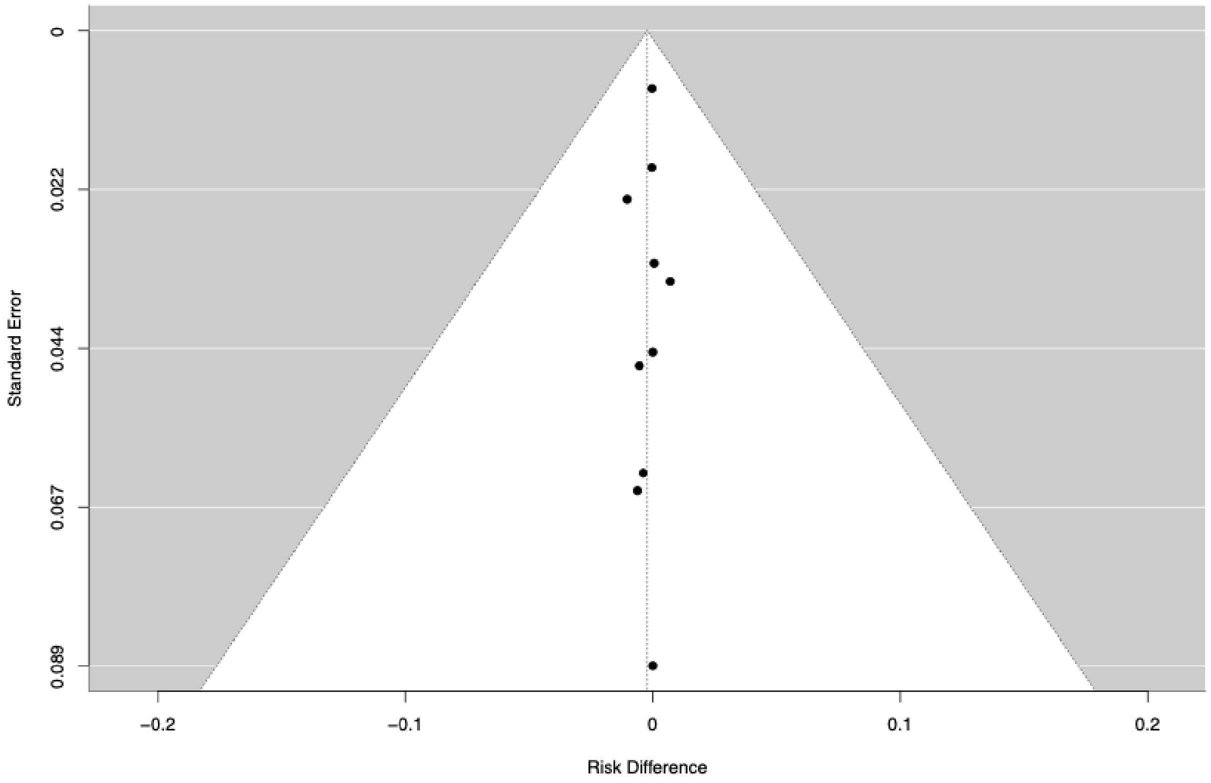

2.19 Funnel plot – Febrile Convulsion

© 2025 Lima JP et al. *JAMA Network Open*.

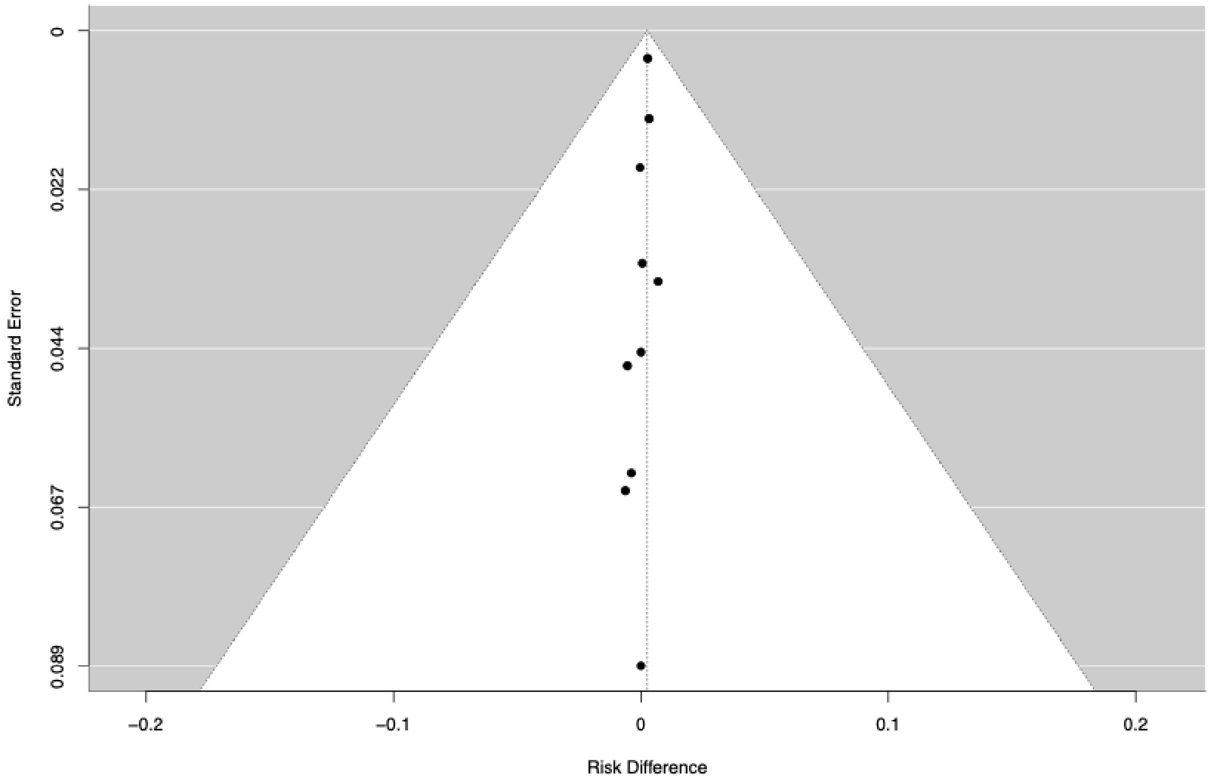

2.20 Funnel plot – Glycosuria

© 2025 Lima JP et al. *JAMA Network Open*.

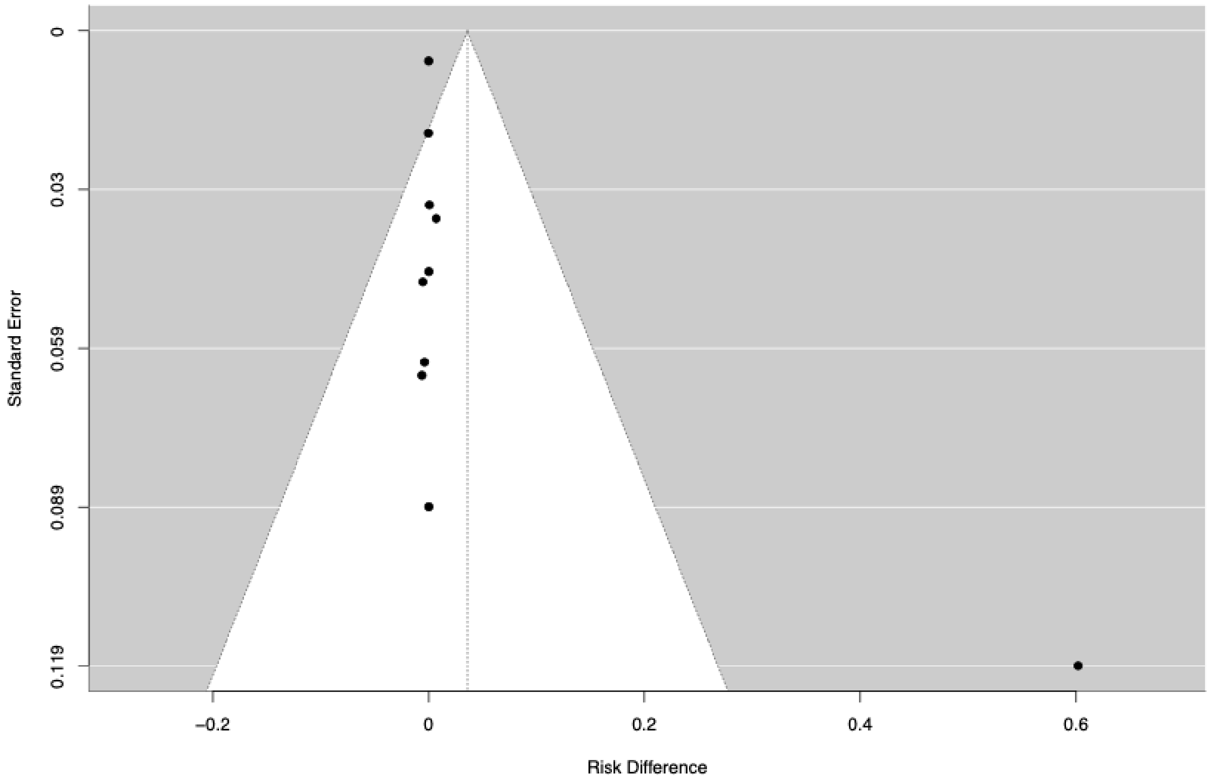

2.21 Funnel plot – Headache

© 2025 Lima JP et al. *JAMA Network Open*.

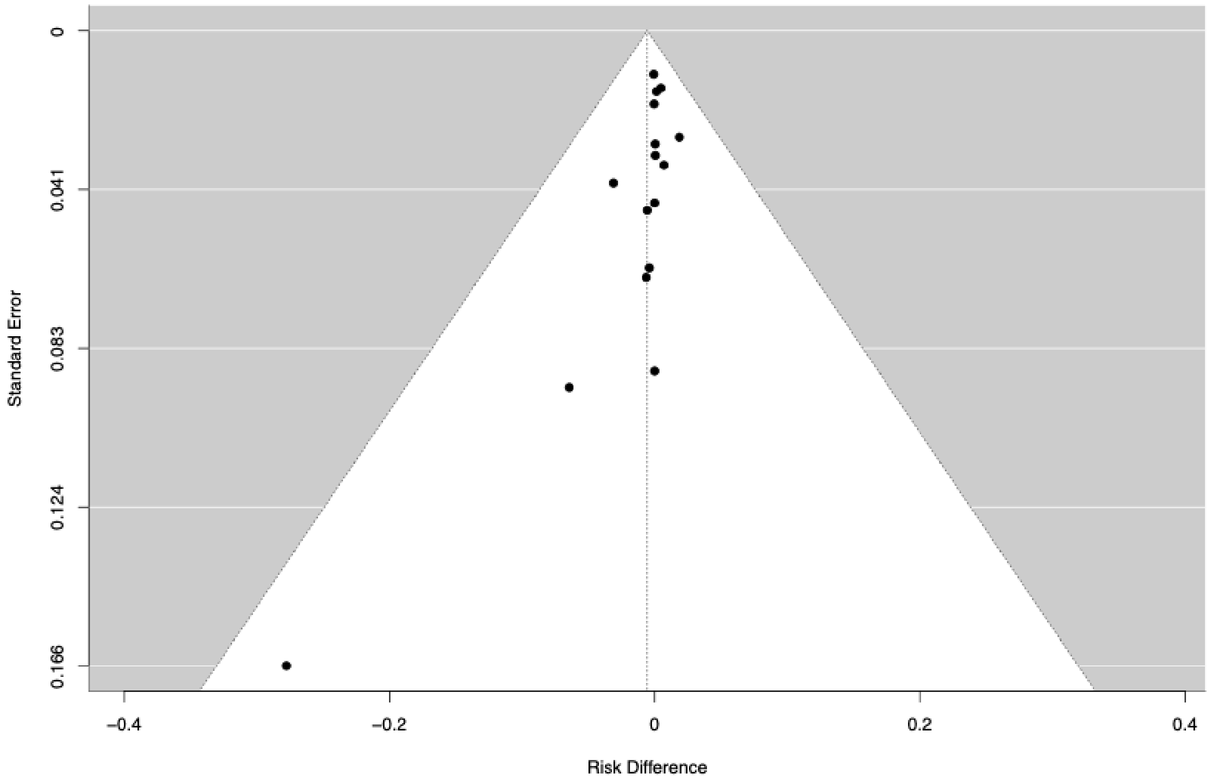

2.22 Funnel plot – Hyperglycemia

© 2025 Lima JP et al. *JAMA Network Open*.

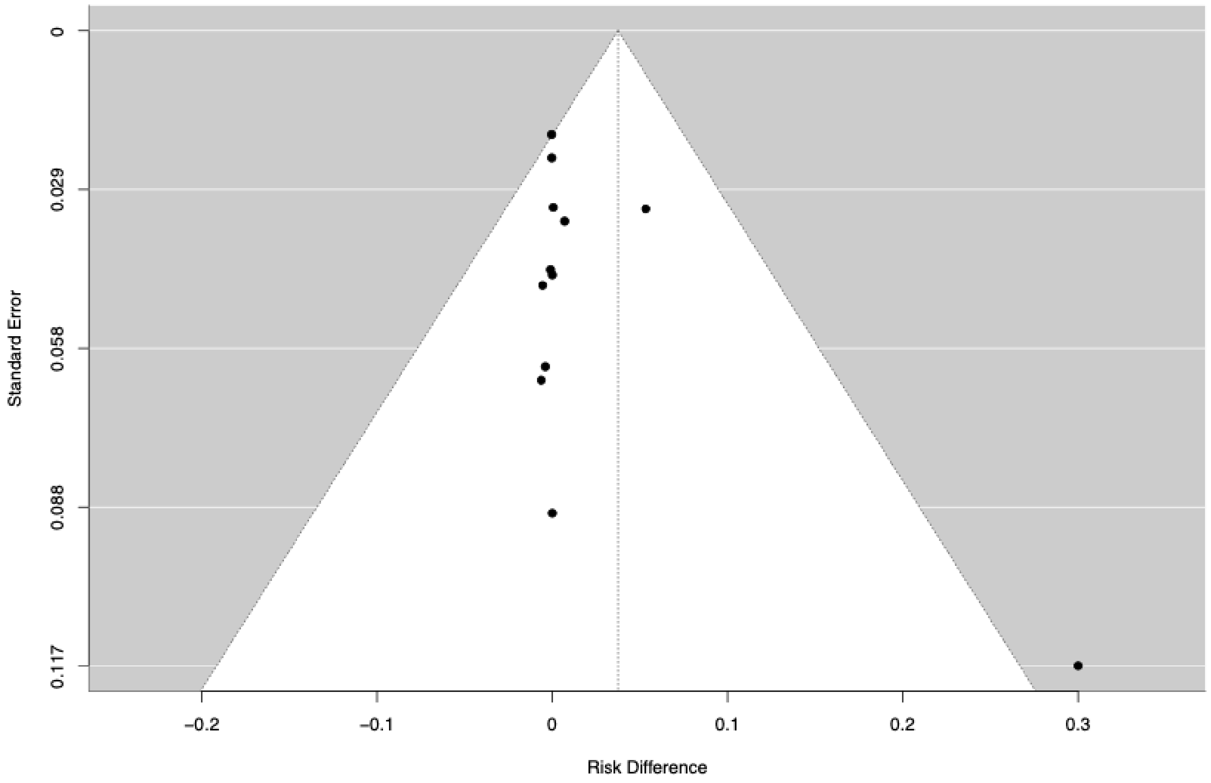

2.23 Funnel plot – Hypertension

© 2025 Lima JP et al. *JAMA Network Open*.

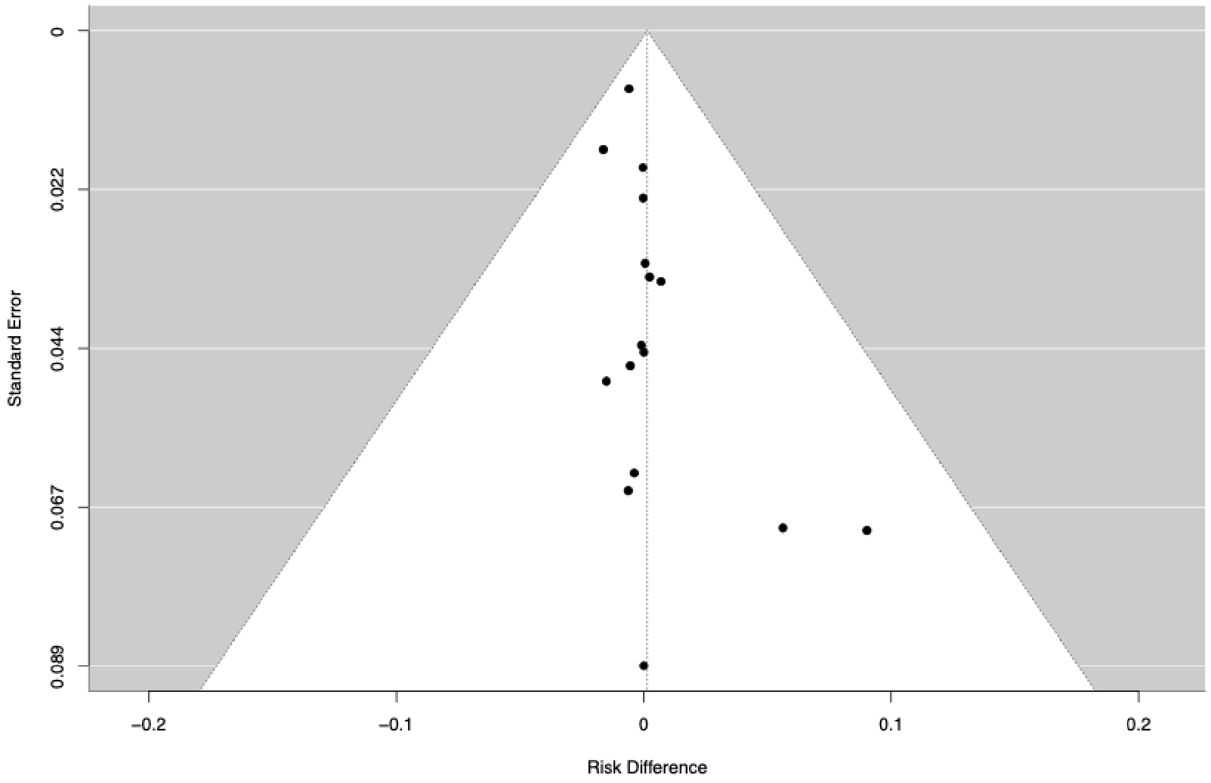

2.24 Funnel plot – Increased Appetite

© 2025 Lima JP et al. *JAMA Network Open*.

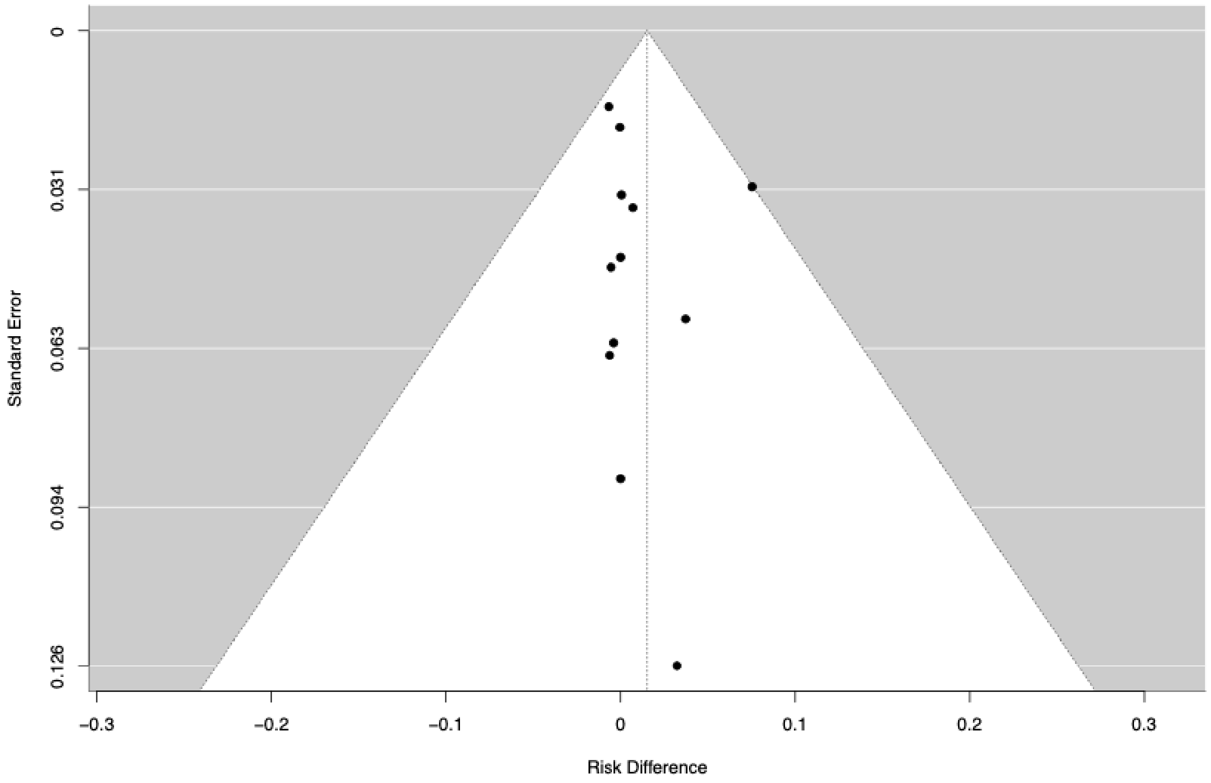

2.25 Funnel plot – Irritability

© 2025 Lima JP et al. *JAMA Network Open*.

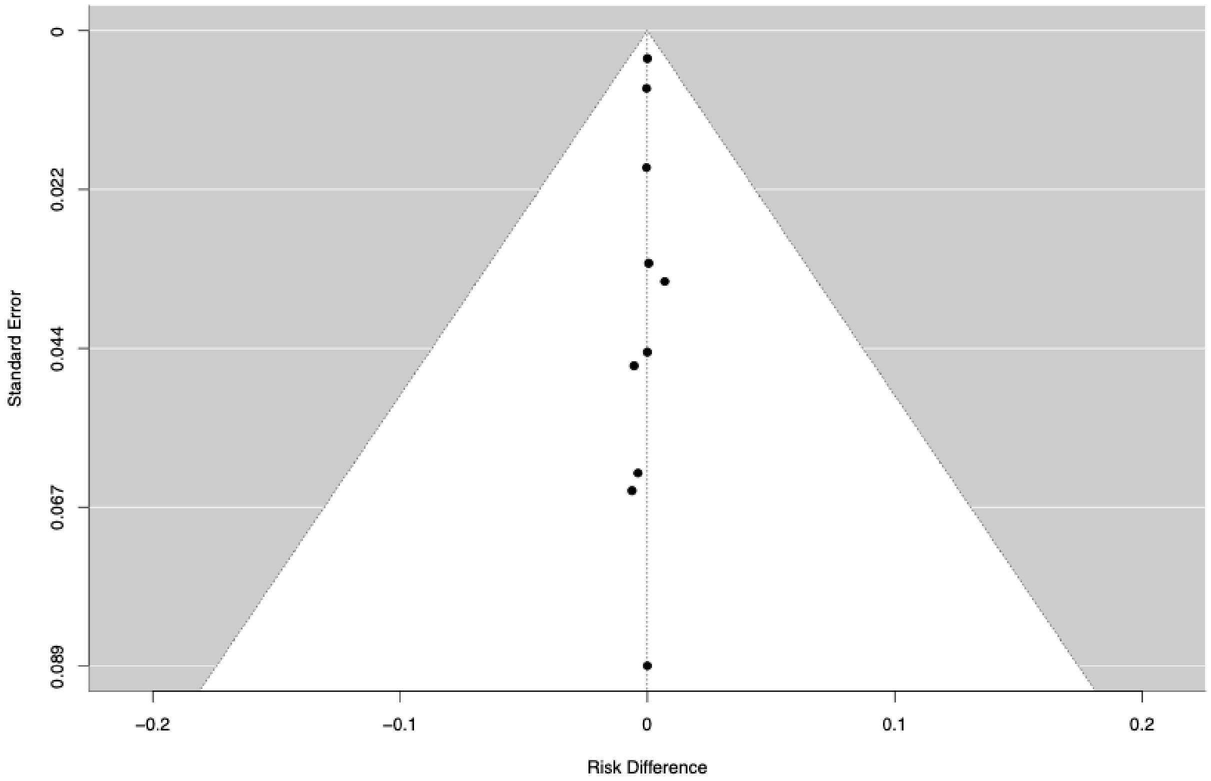

2.26 Funnel plot – Local Site Pain

© 2025 Lima JP et al. *JAMA Network Open*.

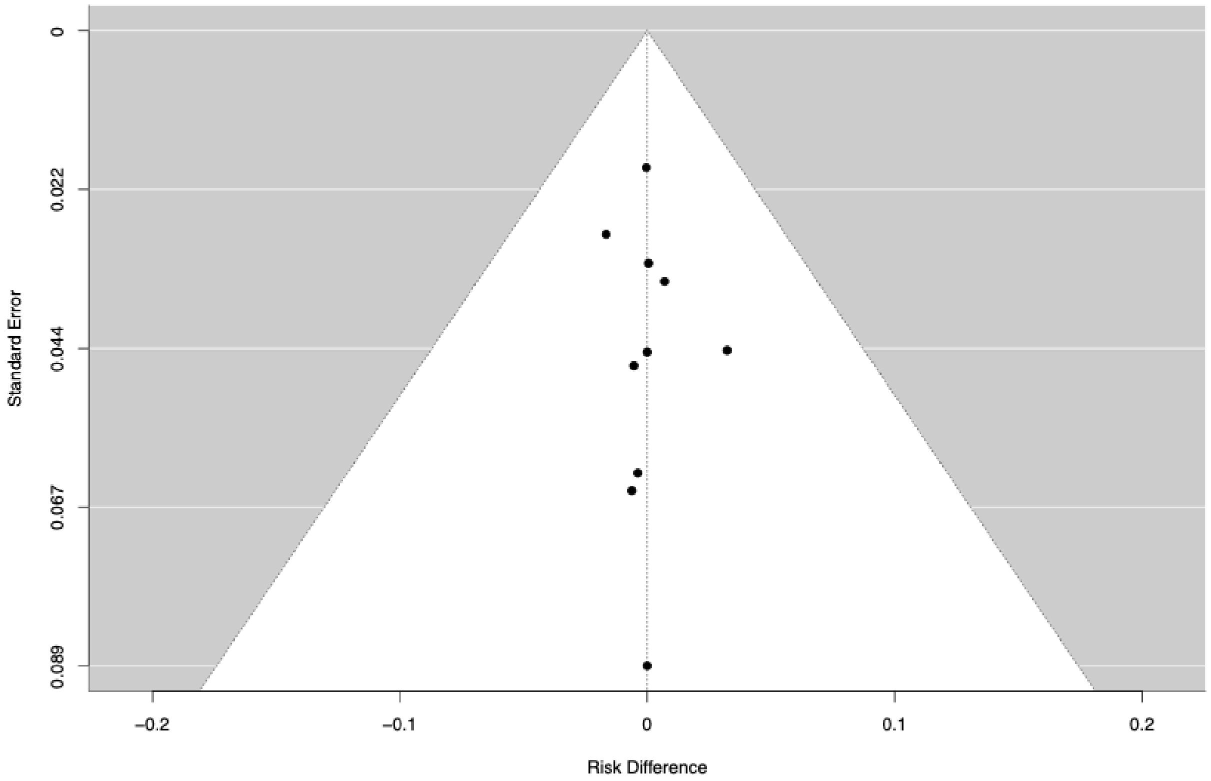

2.27 Funnel plot – Musculoskeletal Pain

© 2025 Lima JP et al. *JAMA Network Open*.

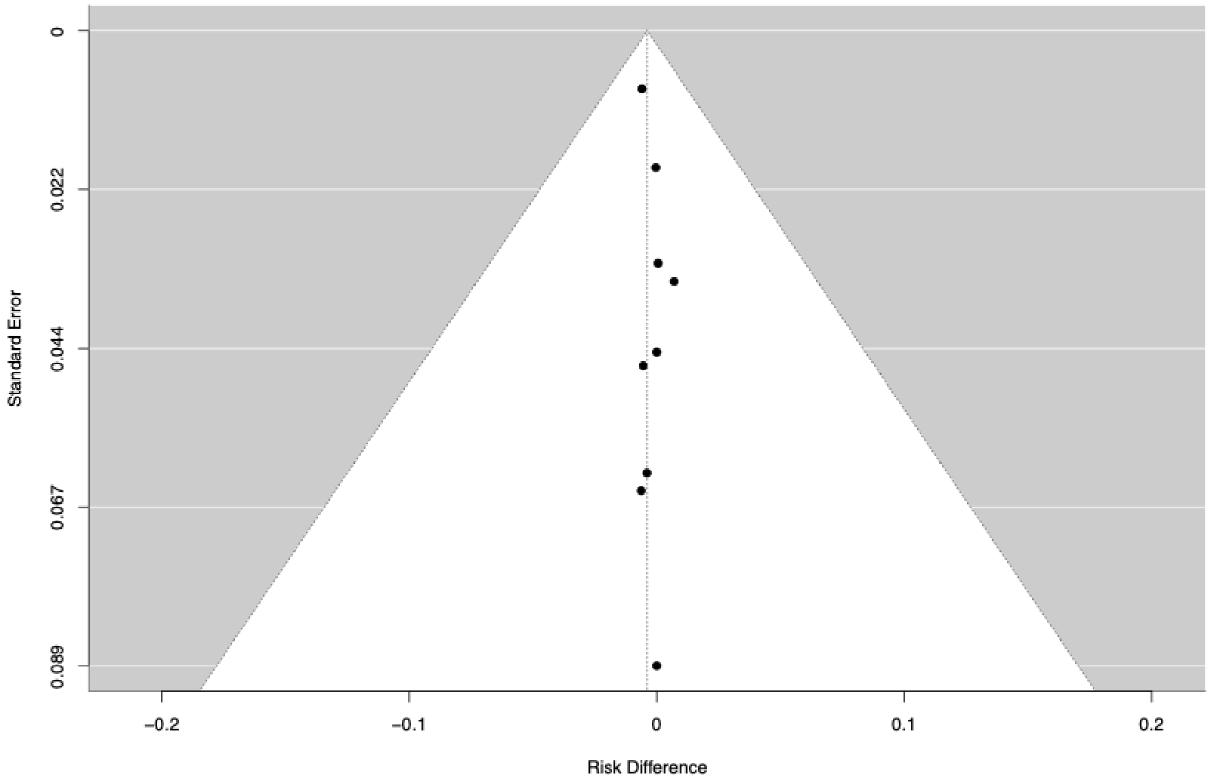

2.28 Funnel plot – Myalgia

© 2025 Lima JP et al. *JAMA Network Open*.

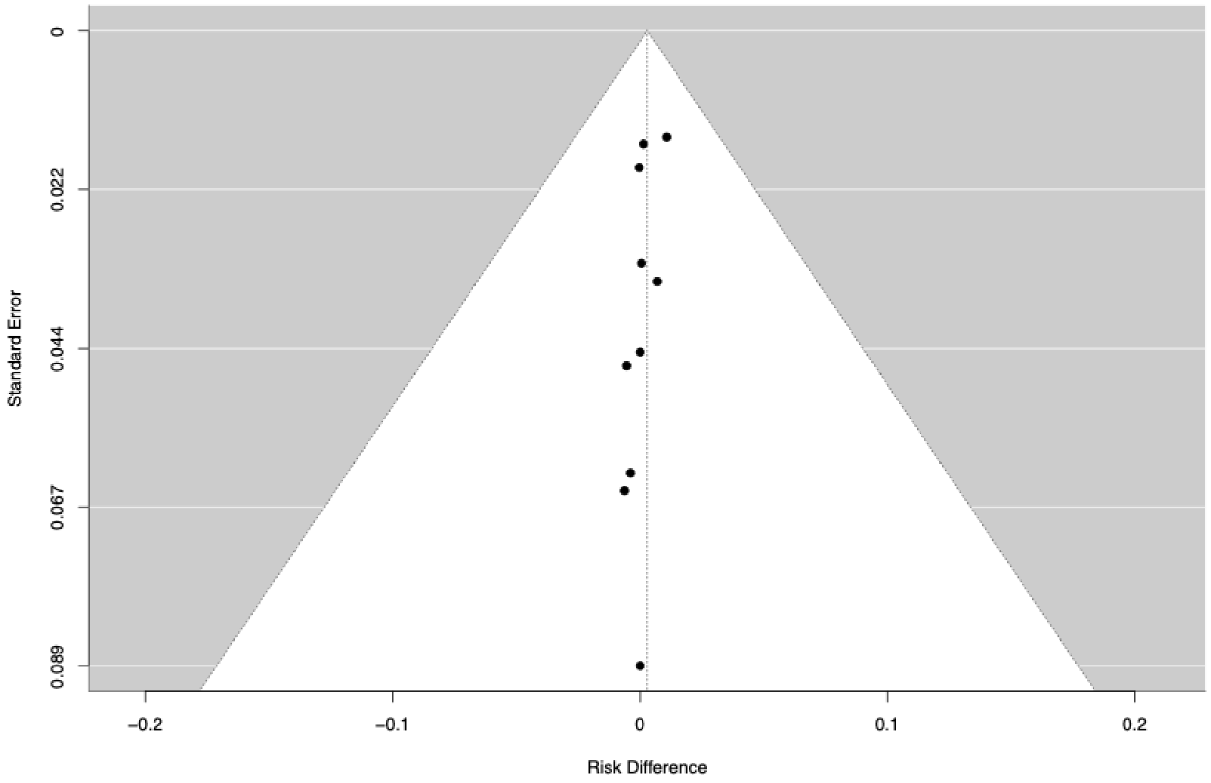

2.29 Funnel plot – Otitis Media

© 2025 Lima JP et al. *JAMA Network Open*.

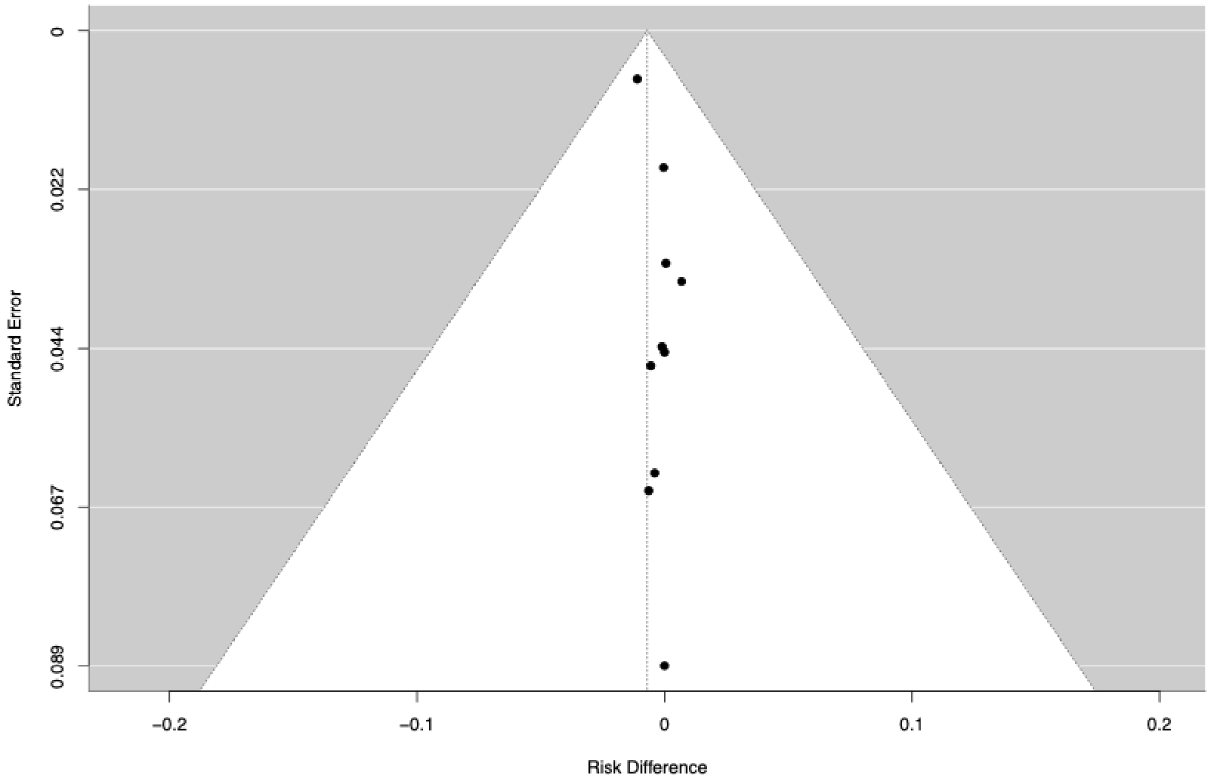

2.30 Funnel plot – Pneumonia

© 2025 Lima JP et al. *JAMA Network Open*.

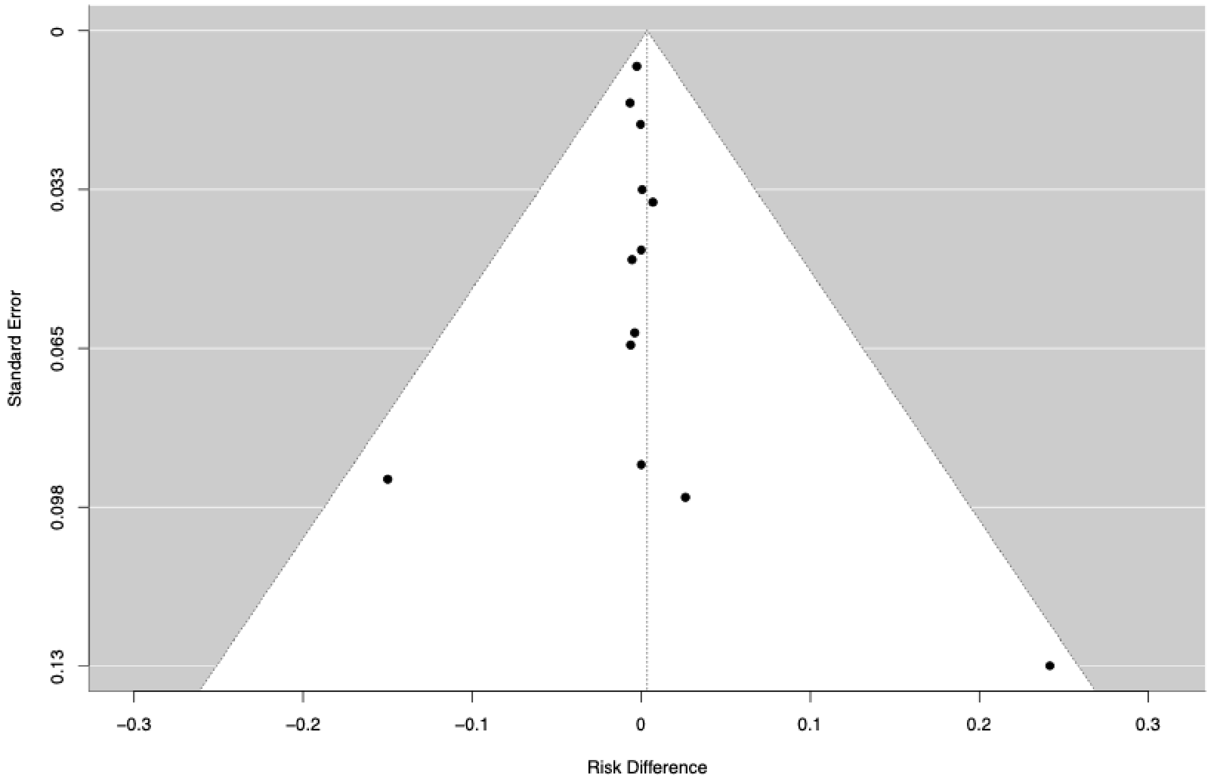

2.31 Funnel plot – Polyuria

© 2025 Lima JP et al. *JAMA Network Open*.

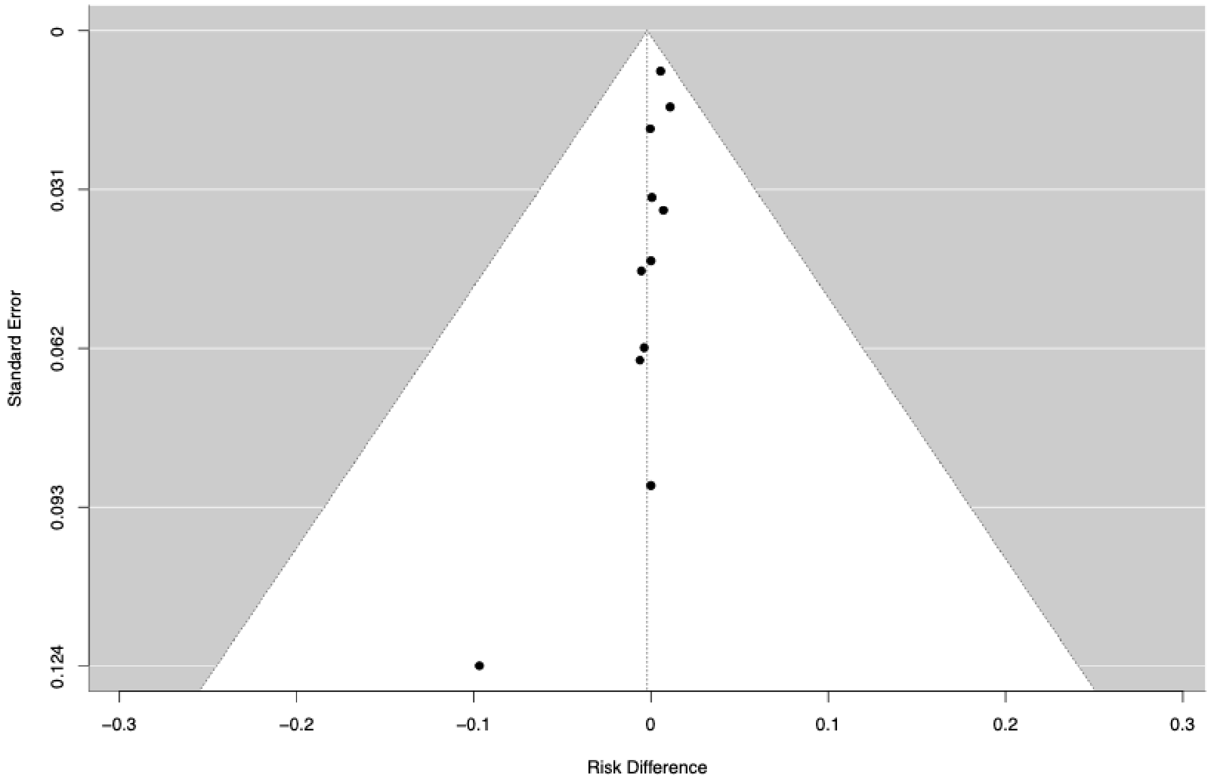

2.32 Funnel plot – Rash or Urticaria

© 2025 Lima JP et al. *JAMA Network Open*.

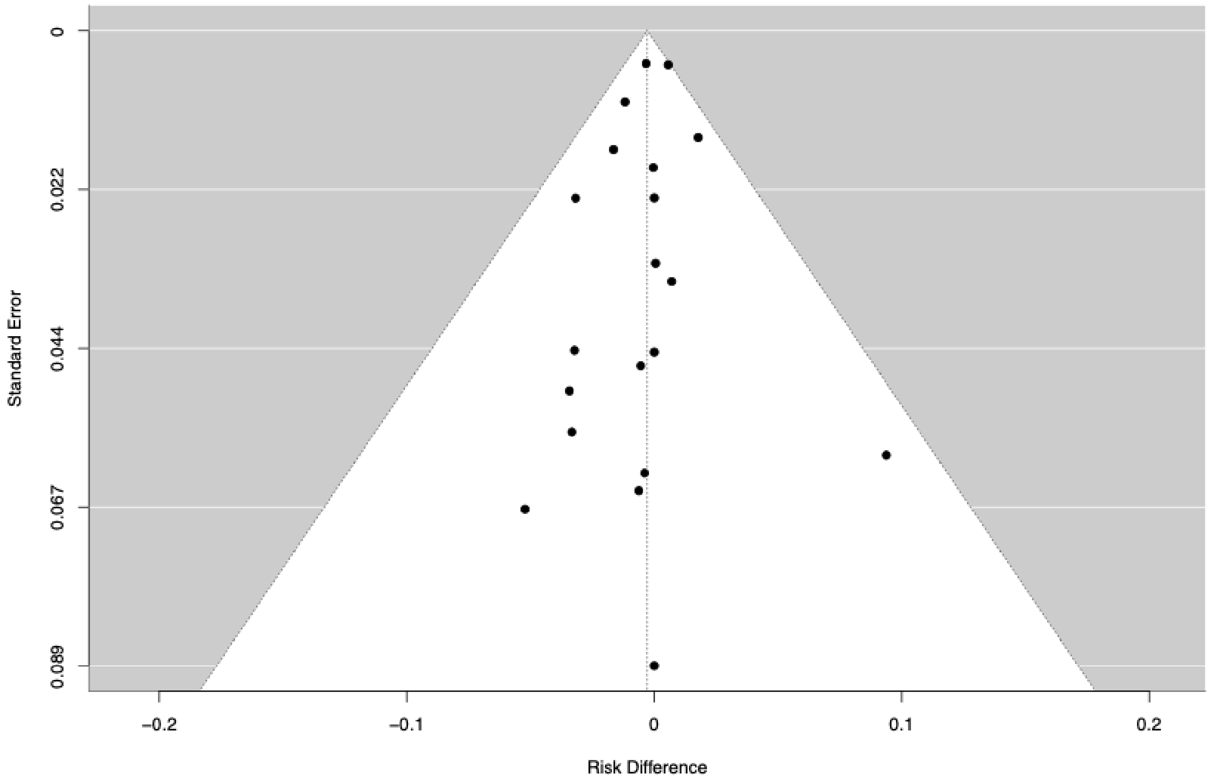

© 2025 Lima JP et al. *JAMA Network Open*.

2.33 Funnel plot – Secondary (Opportunistic) Infection

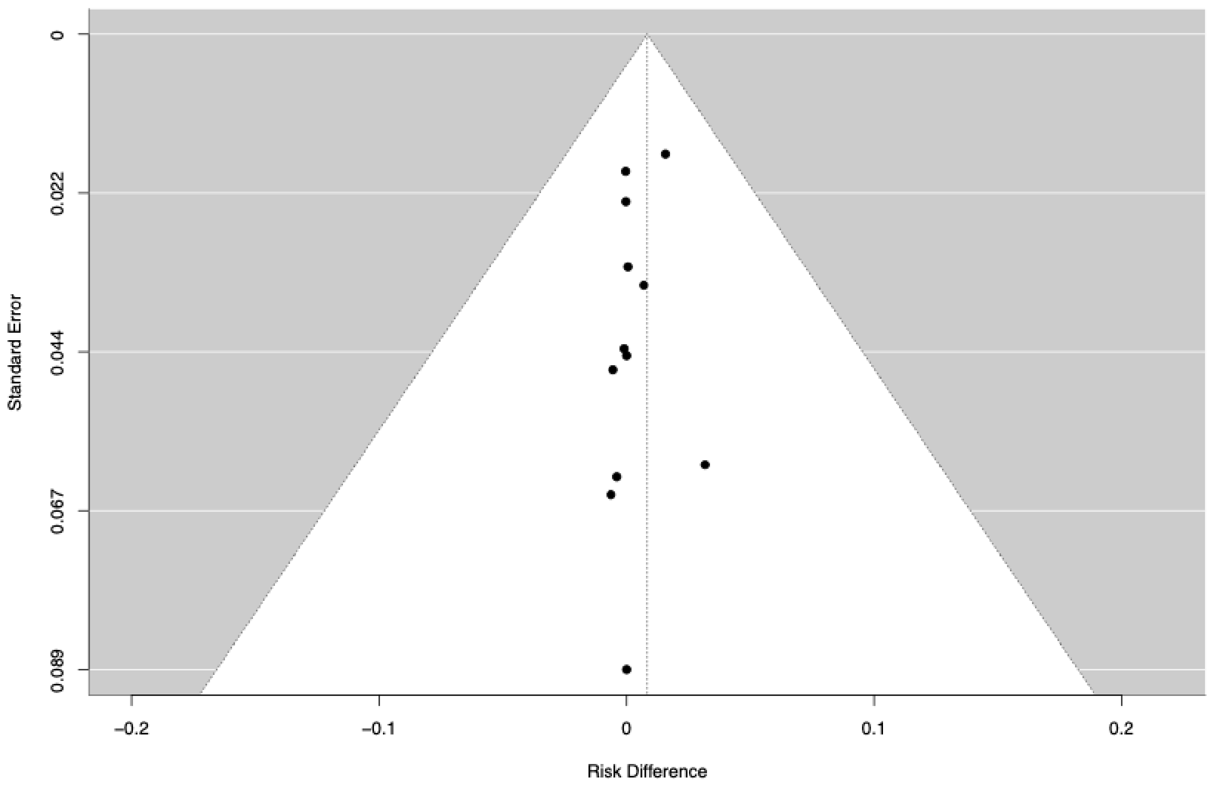

2.34 Funnel plot – Secondary Fever

© 2025 Lima JP et al. *JAMA Network Open*.

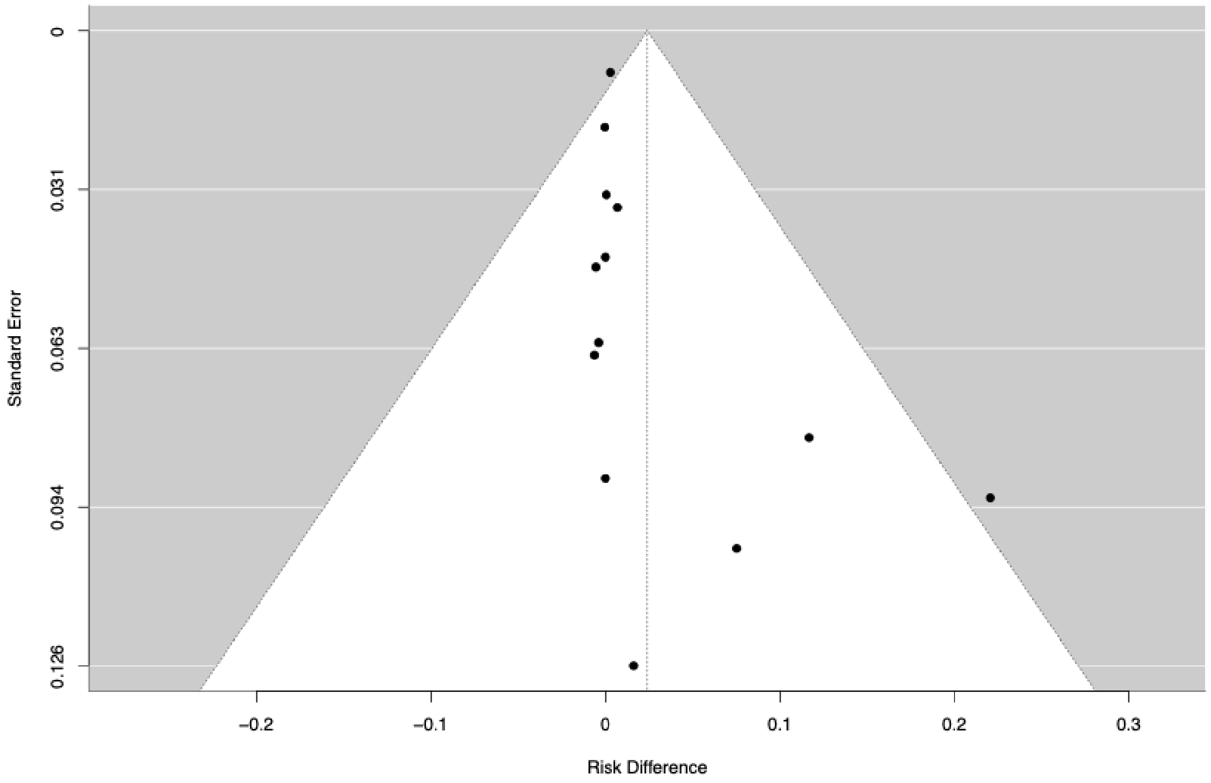

2.35 Funnel plot – Sleep Problems

© 2025 Lima JP et al. *JAMA Network Open*.

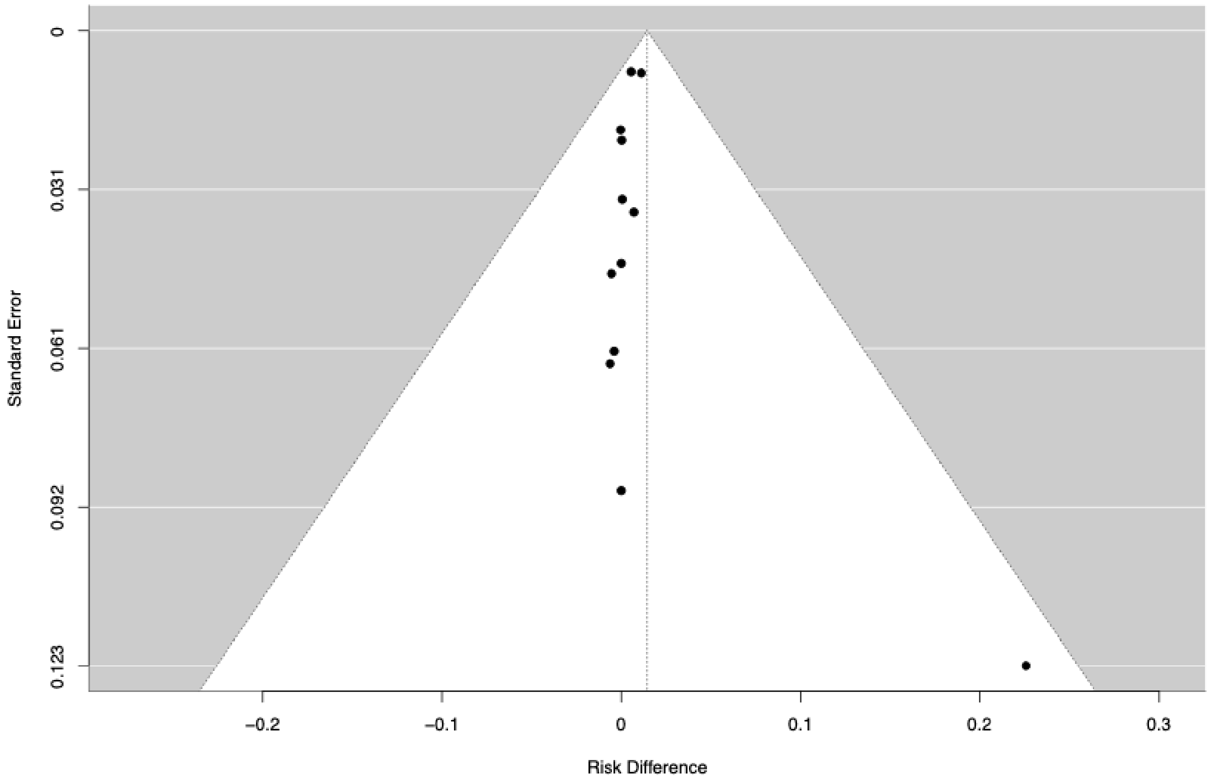

2.36 Funnel plot – Tremor or Hyperactivity

© 2025 Lima JP et al. *JAMA Network Open*.

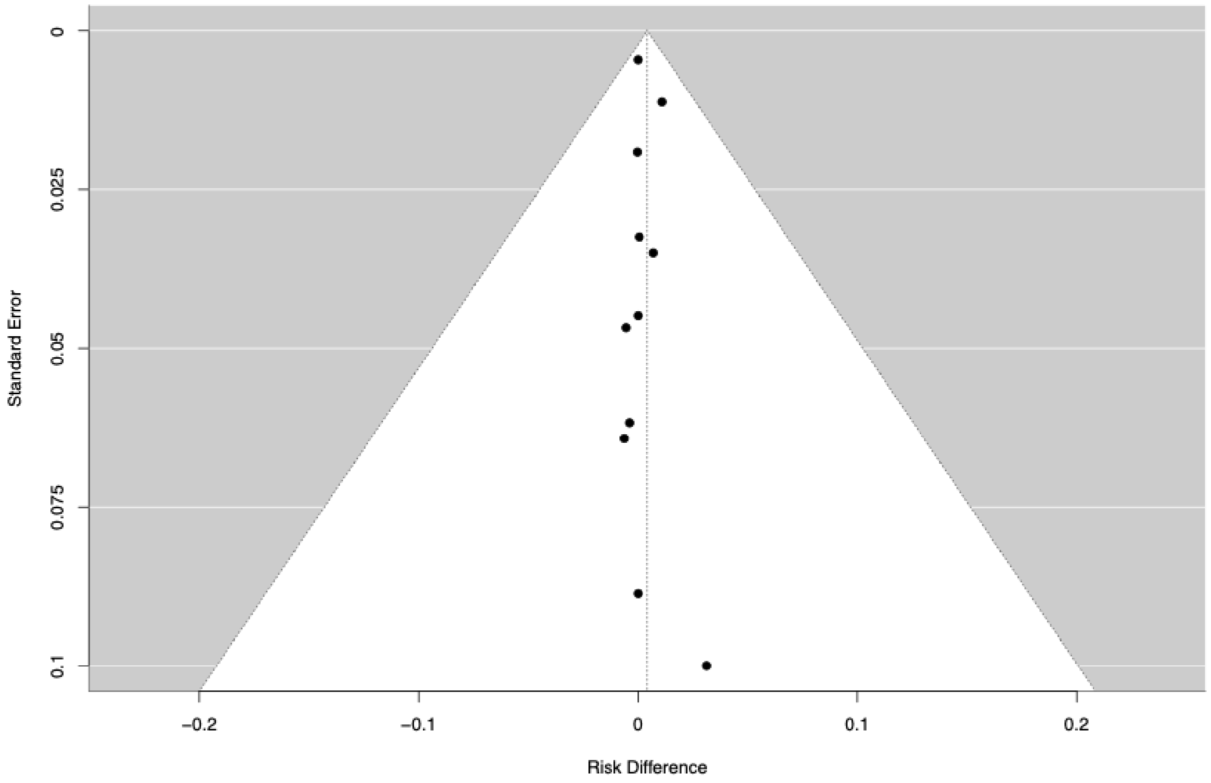

2.37 Funnel plot – Urinary Tract Infection

© 2025 Lima JP et al. *JAMA Network Open*.

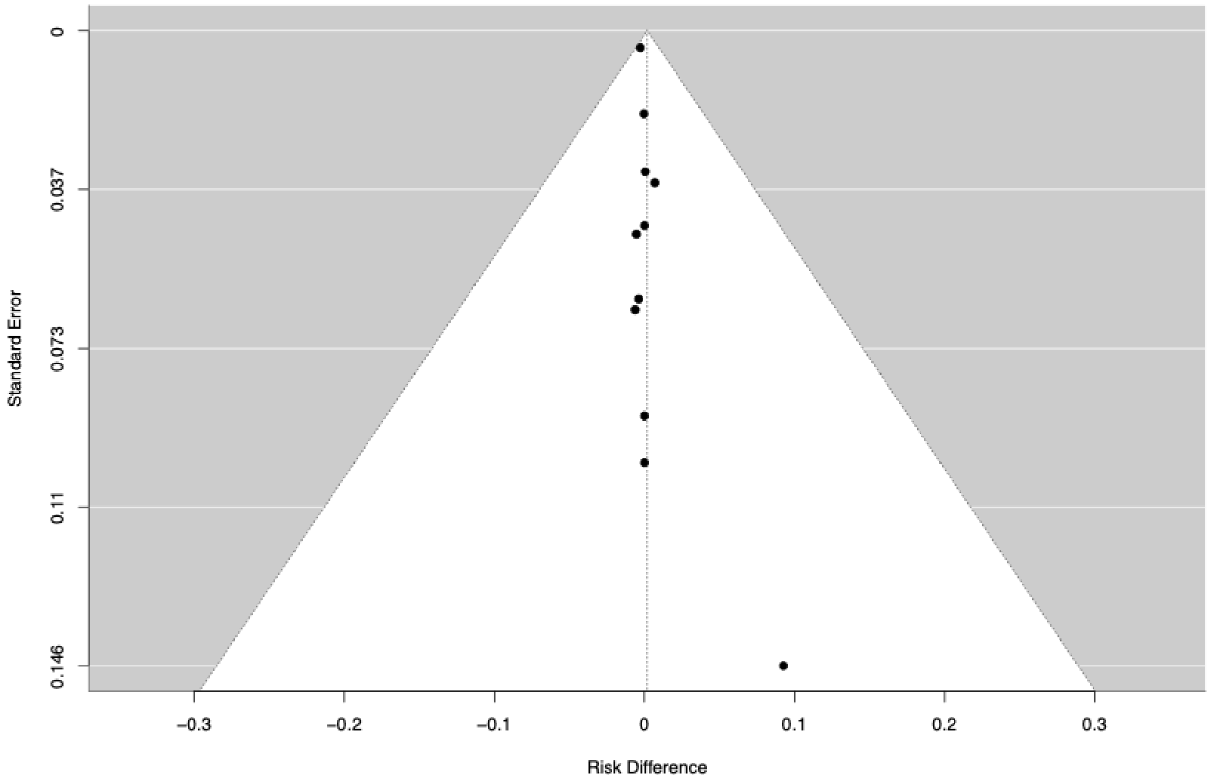

© 2025 Lima JP et al. *JAMA Network Open*.

eTable1. Non-poolable adverse events

Author, Year	Adverse Events	Corticosteroids (events/n)	Placebo or usual care (events/n)
Babl 2022	Change in vision	1/93	0/94
	Hair loss	0/93	2/18
Bernini 1998	Osteonecrosis	0/22	1/97
	Psychosis	0/22	6/31
Bjornson 2004	Bleeding from ear	0/359	1/361
	Bronchitis	3/359	1/361
	Dehydration	1/359	0/361
	Eye discharge	1/359	0/361
	Nasal discharge	1/359	3/170
	Respiratory syncytial virus (RSV) infection	1/259	0/94
	Sinusitis	0/359	1/170
	Sore throat	1/359	0/361
	Streptococcal throat infection	1/359	13/31
	Uncomplicated varicella	0/359	6/31

© 2025 Lima JP et al. *JAMA Network Open*.

Carcao 2020	Allergic reaction	0/14	2/18
Csonka 2003	Restlessness	3/113	0/41
Dudley 2013	Bruising/skin problems	5/180	3/170
	Jaundice	1/180	1/170
	Malaise	1/180	0/170
	Nose Bleed	2/180	1/170
	Stevens Johnson syndrome	1/180	8/275
Francis 2018	Constipation	1/179	1/170
	Ear Pain	1/179	1/170
	Finger infection	0/179	1/170
	Flushed cheeks	1/179	0/55
	Frustration	0/179	1/19
	Parotitis	0/179	0/15
Hoffman 1988	Bacteremia	1/19	2/19
	Hypoglycemia	0/19	0/26
	Pulmonary edema	1/19	0/21
Newburger 2007	Anaphylaxis	0/101	1/97
	Hypotension	5/101	5/170

© 2025 Lima JP et al. *JAMA Network Open*.

	Shock	1/101	0/15
Ogata 2012	Bradycardia	2/22	0/26
	Hypothermia	6/22	0/170
Olympia 2005	Swollen legs	0/57	0/21
Pierson 1974	Hypokalemia	0/30	1/170
	Muscular weakness	0/30	0/15
	Psychological reactions	0/30	0/21
Ranakusuma 2020	Anxiety	4/31	6/31
	Drowsiness	23/31	13/31
	Dry mouth	7/31	6/31
	Weight gain	13/31	2/117
Schaad 1993	Haemopoietic Abnormatities	0/60	3/21
	Hepatic dysfunction	0/60	0/97
	Renal Abonormatities	0/60	1/361
Shaikh 2020	Fussiness	25/271	2/361
Sundel 2003	Idiopathic thrombocytopenic purpura	1/18	0/170
	Rigors	1/18	4/31
Tagarro 2017	Tranfusion	1/30	1/361

© 2025 Lima JP et al. *JAMA Network Open*.

Tam 2012	Upper respiratory infection	4/150	0/68
Tassniyom 1993	Abscess at cut-down area	1/32	1/31
	Gingivitis	1/32	1/361
	Stiff joint	2/32	1/75
Usta 2014	Electrolyte Disturbances	0/42	0/41

© 2025 Lima JP et al. *JAMA Network Open*.

eTable2. Instrument to assess the Credibility of Effect Modification Analyses (ICEMAN) Version 1.1- Outcome: Gastrointestinal Bleeding

Consider the following important instructions informed by common misapplications of ICEMAN in studies using the instrument

Complete a separate credibility assessment per each effect modifier (e.g., age, comorbidity, drug dose, etc.), outcome (e.g., mortality, stroke, duration of hospital stay), time-point (e.g., 3 months, 6 months), and effect measure (e.g. relative risk, risk difference).

Do not apply ICEMAN if the interaction p-value is 0.1 or larger, i.e., provides very little statistical support for the existence of an effect modification (ICEMAN is designed to address the possible claim of an effect modification rather than the claim of no effect modification).

Response options on the left indicate definitely or probably reduced credibility, response options on the right probably or definitely increased credibility

Completely unclear should be interpreted as probably reduced credibility.

To ensure transparency, provide a supporting comment under each question that provides a rationale for the rating.

To ensure transparency, provide a copy of the completed ICEMAN instrument in the supplement of your article.

CREDIBILITY ASSESSMENT

Essential preliminary considerations to define the possible effect modification of interest

State a single candidate effect modifier (e.g., age or comorbidity): Route (Intravenous or Intramuscular vs Oral)

Was the effect modifier measured before or at randomization? [**X**] yes, continue [ ] no, stop here and refer to manual for further instructions

State a single outcome and time-point (e.g., mortality at 1 year follow-up): Gastrointestinal Bleeding, latest follow-up

State a single effect measure (e.g., relative risk or risk difference): Risk Diffence

© 2025 Lima JP et al. *JAMA Network Open*.

1: Is the analysis of effect modification based on comparison within rather than between trials?

[X] Completely between	[ ] Mostly between or unclear	[ ] Mostly within	[ ] Completely within
Subgroup analysis or meta-regression comparing overall effects of each individual trial. This is typical for aggregate data meta-analysis.	Subgroup analysis or meta-regression with most information coming from overall effects, but some trials providing within-trial subgroup information	Most trials providing within-trial subgroup information; or individual participant data analysis that combines within and between trial information	All trials providing within-trial subgroup information or individual participant data; and the analysis separates within from between trial information, e.g., meta-analysis of interactions

Comment: NA

2: For within-trial comparisons, is the effect modification similar from trial to trial? [X] Not applicable: no or one within-RCT comparison

[ ] Definitely not similar	[ ] Probably not similar or unclear	[ ] Mostly similar	[ ] Definitely similar
Effect modification reported for two or more trials and clearly different directions	Effect modification not reported for individual trials or too imprecise to tell	Effect modification reported for two or more trials, mostly similar in direction, but considerable differences in magnitude	Effect modification reported for two or more trials, similar in direction, only some differences in magnitude

Comment: NA

3: For between-trial comparisons, is the number of trials large? [ ] Not applicable: no between RCT comparison

[ ] Very small	[ ] Rather small or unclear	[X] Rather large	[ ] Large
1 or 2 or in smallest subgroup; 5 or less in continuous meta-regression	3-4 in smallest subgroup; 6-10 in continuous meta-regression	5-9 in smallest subgroup; 11 to 15 in continuous meta-regression	10 or more in smallest subgroup; more than 15 in continuous meta-regression

Comment: NA

4: Was the direction of effect modification correctly hypothesized a priori?

[ ] Definitely no	[ ] Probably no or unclear	[ ] Probably yes	[X] Definitely yes
Clearly post-hoc or results inconsistent with hypothesized direction or biologically very implausible	Vague hypothesis or hypothesized direction unclear	No prior protocol available but unequivocal statement of a priori hypothesis with correct direction of effect modification	Prior protocol available and includes correct specification of direction of effect modification, e.g., based on a biologic rationale

Comment: NA

© 2025 Lima JP et al. *JAMA Network Open*.

5: Does a test for interaction suggest that chance is an unlikely explanation of the apparent effect modification? (consider irrespective of number of effect modifiers)			
[ ] Chance a very likely explanation	[ ] Chance a likely explanation or unclear	[X] Chance may not explain	[ ] Chance an unlikely explanation
Interaction or meta-regression p-value >0.05	Interaction or meta-regression p-value ≤0.05 and >0.01, or no test of interaction reported and not computable	Interaction or meta-regression p-value ≤0.01 and >0.005	Interaction or meta-regression p-value ≤0.005
Comment: NA			
6: Did the authors test only a small number of effect modifiers or consider the number in their statistical analysis?			
[ ] Definitely no	[ ] Probably no or unclear	[ ] Probably yes	[X] Definitely yes
Explicitly exploratory analysis or large number of effect modifiers tested (e.g., greater than 10) and multiplicity not considered in analysis	No mention of number or 4-10 effect modifiers tested and number not considered in analysis	No protocol available but unequivocal statement of 3 or fewer effect modifiers tested	Protocol available and 3 or fewer effect modifiers tested or number considered in analysis
Comment:			
7: Did the authors use a random effects model?			
[X] Definitely no	[ ] Probably no or unclear	[ ] Probably yes	[ ] Definitely yes
Fixed (or common) effect or fixed effects model explicitly stated	Probably fixed effect(s) model	Probably random (or mixed) effects	Random (or mixed) effects explicitly stated
Comment:			
8: If the effect modifier is a continuous variable, were arbitrary cut points avoided? [X] not applicable: not continuous			
[ ] Definitely no	[ ] Probably no or unclear	[ ] Probably yes	[ ] Definitely yes
Analysis based on exploratory cut point(s), e.g., picking cut point associated with highest interaction p-value	Analysis based on cut point(s) of unclear origin	Analysis based on pre-specified cut point(s), e.g., suggested by prior RCT	Analysis based on the full continuum, e.g., assuming a linear or logarithmic relationship
Comment: NA			

© 2025 Lima JP et al. *JAMA Network Open*.

9 Optional: Are there any additional considerations that may increase or decrease credibility? (manual section 3.9) [X] not applicable

[ ] Yes, probably decrease [ ] Yes, probably increase

Comment: NA

10: How would you rate the overall credibility of the proposed effect modification?
The overall rating should be driven by the items that decrease credibility. The following provides a sensible strategy:
All responses definitely or probably decrease credibility or unclear → very low
Two or more responses definitely decrease credibility → maximum usually low even if all other responses satisfy credibility criteria
One response definitely decreases credibility → maximum usually moderate even if all other responses satisfy credibility criteria
Two responses probably decrease credibility → maximum usually moderate even if all other responses satisfy credibility criteria
No response options definitely or probably decrease credibility → high very likely

Place a mark on the continuous line (or type "x" in editable version)

Very low credibility	**Low credibility**	Moderate credibility	High credibility
Minimal to no support for effect modification;<br>Use overall effect for each subgroup	**Some but insufficient support for effect modification;<br>Use overall effect for each subgroup but note remaining uncertainty**	Likely effect modification;<br>Use separate effects for each subgroup but note remaining uncertainty	Very likely effect modification;<br>Use separate effects for each subgroup

Comment: NA

© 2025 Lima JP et al. *JAMA Network Open*.

eFigure3. Subgroup Analyses

3.1 Gastrointestinal bleeding (Subgroup - route: intravenous/intramuscular vs oral)

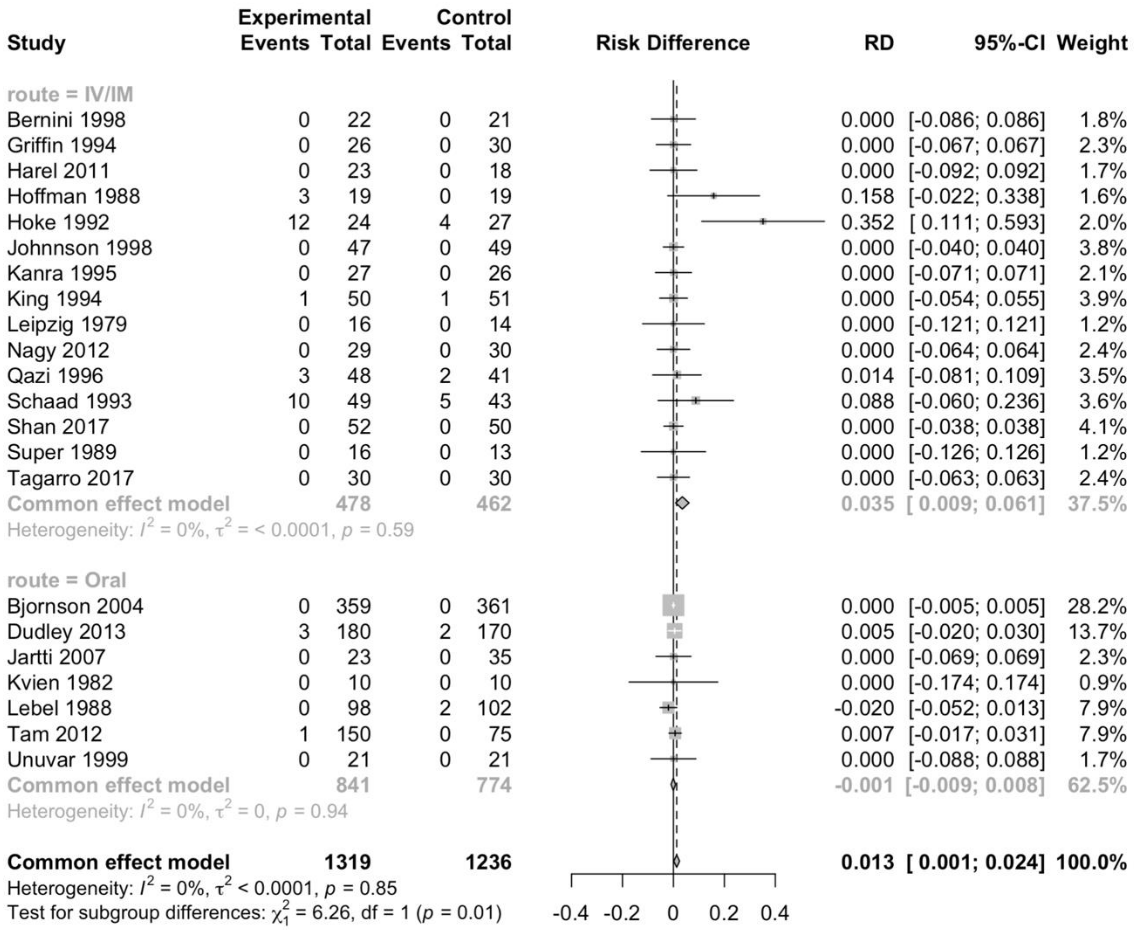

© 2025 Lima JP et al. *JAMA Network Open*.

CI: confidence interval IV/IM: intravenous/intramuscular; RD: risk difference

3.2 Gastrointestinal bleeding (subgroup: condition)

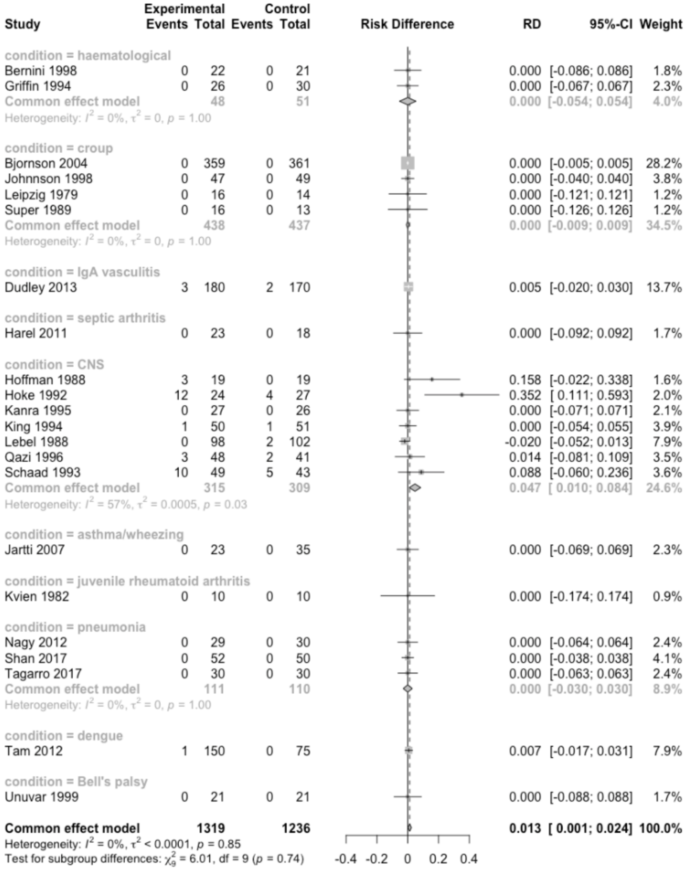

© 2025 Lima JP et al. *JAMA Network Open*.

CNS= central nervous system; CI= confidence interval RD=risk difference

3.3 Sleep problems (subgroup: condition)

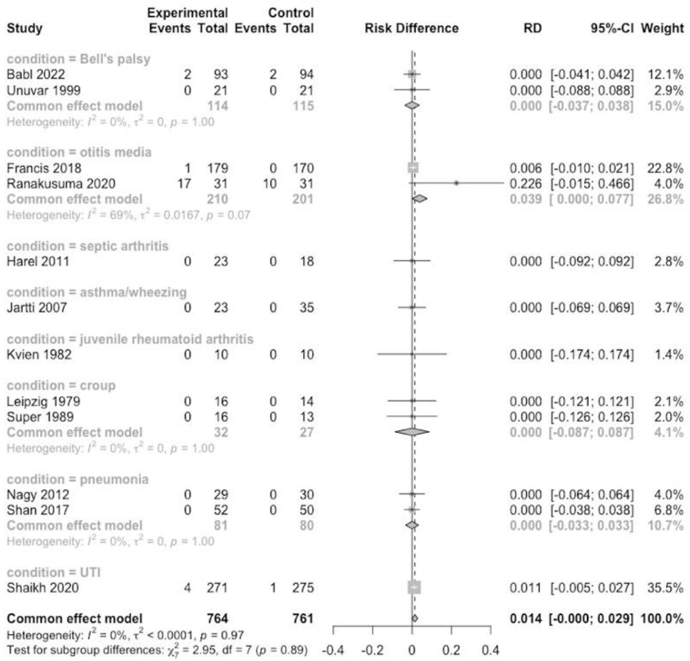

3.4 Change in behavior (subgroup: condition)

© 2025 Lima JP et al. *JAMA Network Open*.

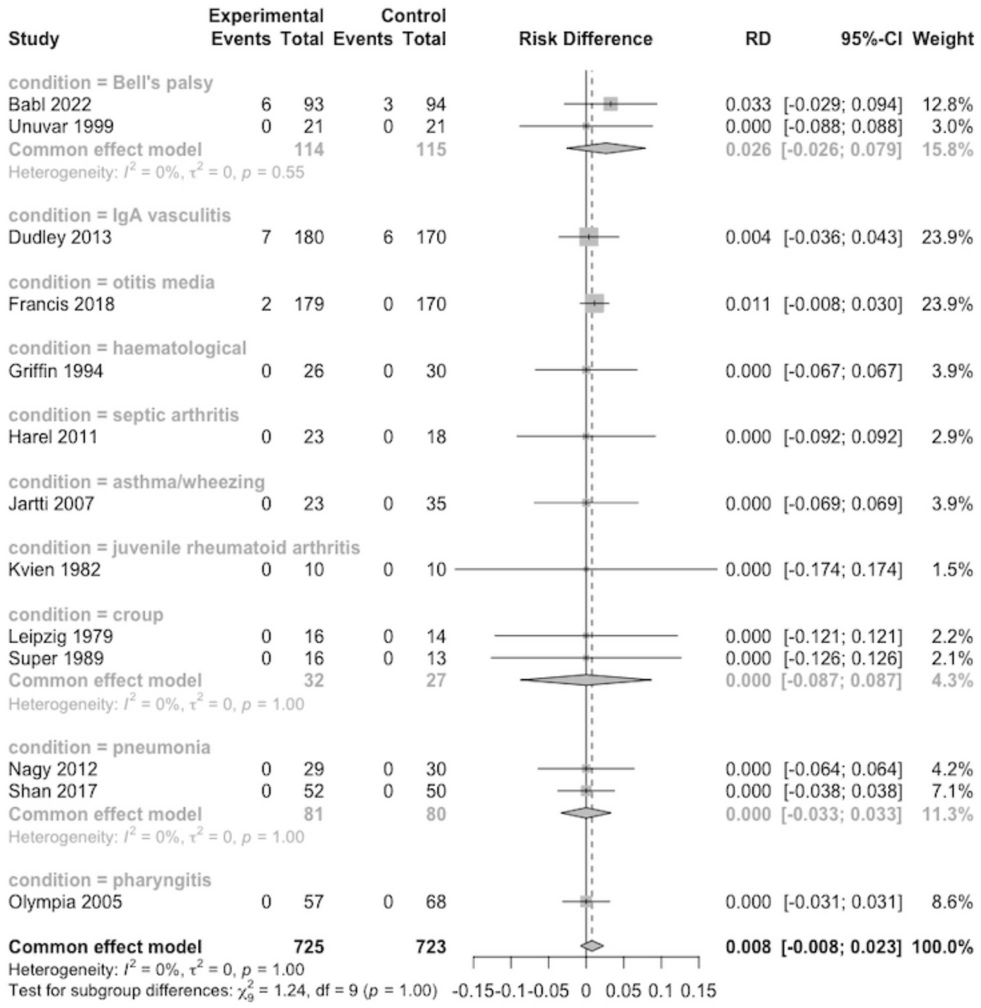

3.5 Hyperglycemia (subgroup: condition)

© 2025 Lima JP et al. *JAMA Network Open*.

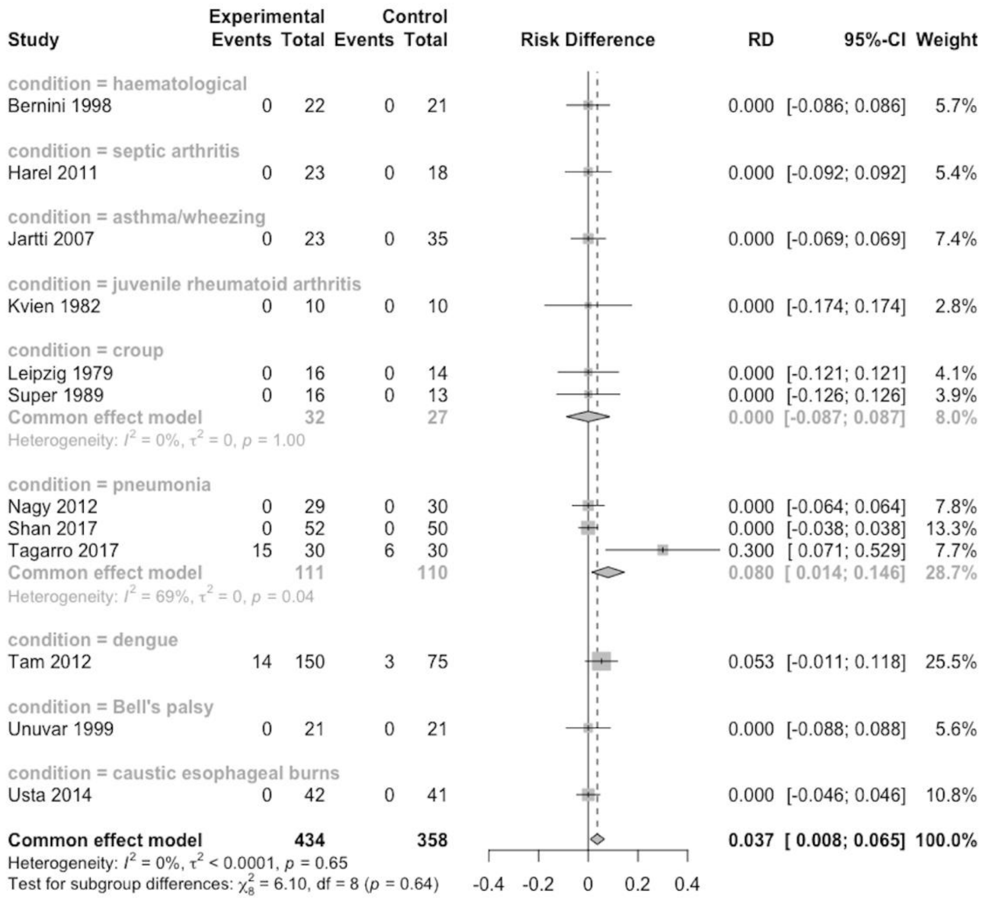

3.6 Serious Adverse Events (subgroup: condition)

© 2025 Lima JP et al. *JAMA Network Open*.

Study	Experimental Events	Experimental Total	Control Events	Control Total	RD	95%-CI	Weight
condition = Bell's palsy							
Babl 2022	0	93	0	94	0.000	[-0.021; 0.021]	5.5%
Unuvar 1999	0	21	0	21	0.000	[-0.088; 0.088]	1.3%
Common effect model		114		115	0.000	[-0.024; 0.024]	6.8%
Heterogeneity: $I^2 = 0\%$, $\tau^2 = 0$, $p = 1.00$							
condition = croup							
Bjornson 2004	0	359	0	361	0.000	[-0.005; 0.005]	21.1%
Leipzig 1979	0	16	0	14	0.000	[-0.121; 0.121]	0.9%
Super 1989	0	16	0	13	0.000	[-0.126; 0.126]	0.9%
Common effect model		391		388	0.000	[-0.009; 0.009]	23.0%
Heterogeneity: $I^2 = 0\%$, $\tau^2 = 0$, $p = 1.00$							
condition = haematological							
Carcao 2020	0	14	0	18	0.000	[-0.116; 0.116]	1.0%
condition = asthma/wheezing							
Foster 2018	0	308	0	304	0.000	[-0.006; 0.006]	18.0%
Jartti 2007	0	23	0	35	0.000	[-0.069; 0.069]	1.7%
Common effect model		331		339	0.000	[-0.008; 0.008]	19.6%
Heterogeneity: $I^2 = 0\%$, $\tau^2 = 0$, $p = 1.00$							
condition = otitis media							
Francis 2018	0	179	1	170	-0.006	[-0.022; 0.010]	10.3%
Ranakusuma 2020	0	31	0	31	0.000	[-0.061; 0.061]	1.9%
Common effect model		210		201	-0.005	[-0.021; 0.011]	12.1%
Heterogeneity: $I^2 = 0\%$, $\tau^2 = 0$, $p = 0.85$							
condition = septic arthritis							
Harel 2011	0	23	0	18	0.000	[-0.092; 0.092]	1.2%
condition = juvenile rheumatoid arthritis							
Kvien 1982	0	10	0	10	0.000	[-0.174; 0.174]	0.6%
condition = pneumonia							
Nagy 2012	0	29	0	30	0.000	[-0.064; 0.064]	1.8%
Shan 2017	0	52	0	50	0.000	[-0.038; 0.038]	3.0%
Tagarro 2017	1	29	1	29	0.000	[-0.094; 0.094]	1.7%
Common effect model		110		109	0.000	[-0.035; 0.035]	6.5%
Heterogeneity: $I^2 = 0\%$, $\tau^2 = 0$, $p = 1.00$							
condition = kawasaki disease							
Newburger 2007	2	101	2	97	-0.001	[-0.040; 0.038]	5.8%
Ogata 2012	0	22	0	26	0.000	[-0.078; 0.078]	1.5%
Common effect model		123		123	-0.001	[-0.036; 0.034]	7.2%
Heterogeneity: $I^2 = 0\%$, $\tau^2 = 0$, $p = 0.99$							
condition = UTI							
Shaikh 2020	7	271	8	275	-0.003	[-0.031; 0.024]	16.0%
condition = dengue							
Tam 2012	20	150	10	75	0.000	[-0.094; 0.094]	5.9%
**Common effect model**		**1747**		**1671**	**-0.001**	**[-0.010; 0.008]**	**100.0%**
Heterogeneity: $I^2 = 0\%$, $\tau^2 = 0$, $p = 1.00$							
Test for subgroup differences: $\chi^2_{10} = 0.35$, df = 10 ($p = 1.00$)							

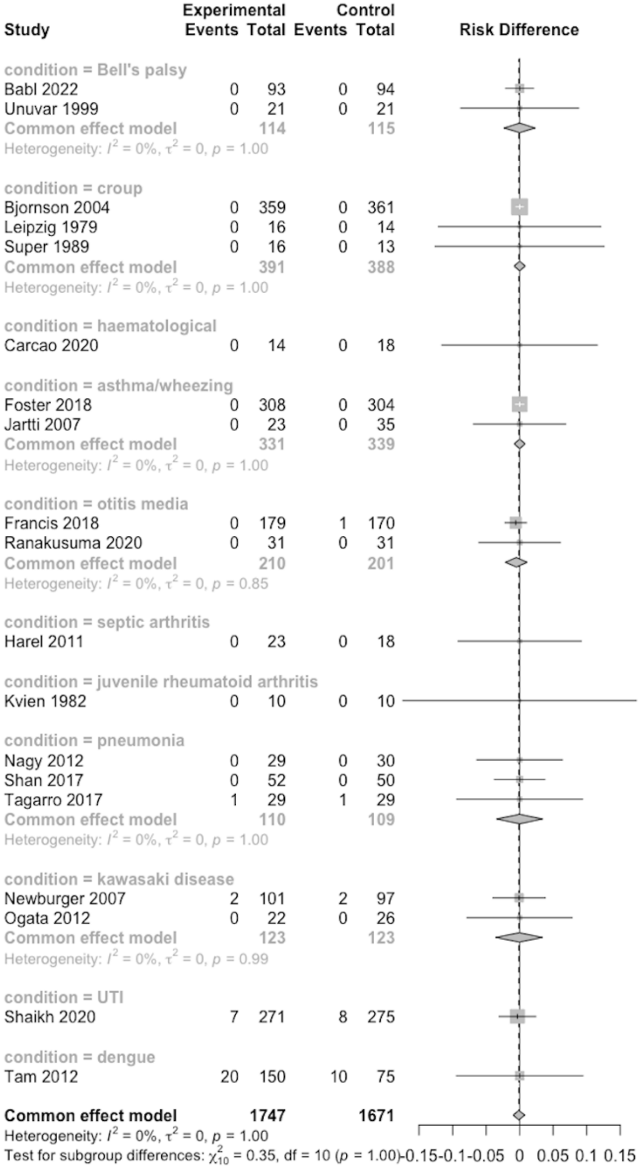

© 2025 Lima JP et al. *JAMA Network Open*.

3.7 Adverse events leading to discontinuation (subgroup: condition)

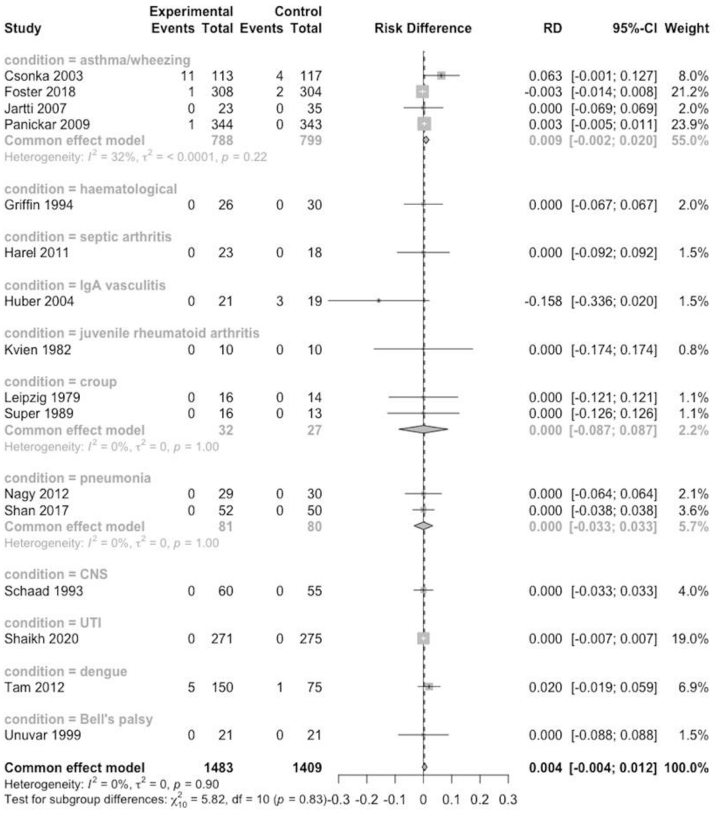

eFigure4. Sensitivity Analyses – Peto Odds Ratio

© 2025 Lima JP et al. *JAMA Network Open*.

4.1 Serious Adverse Events (SAE)

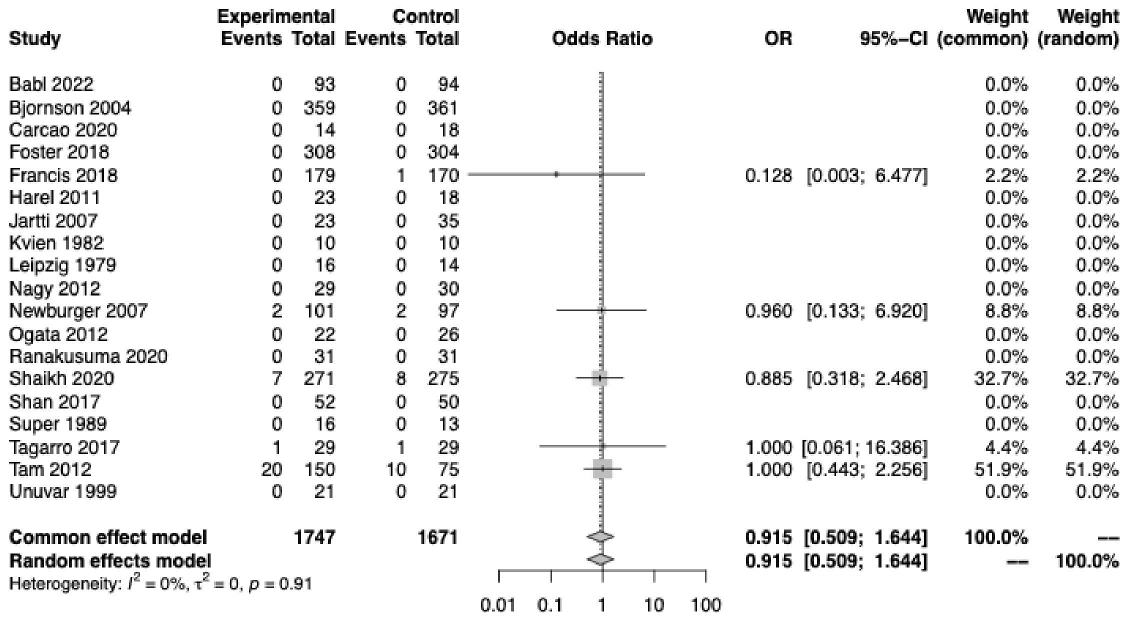

4.2 Adverse events leading to discontinuation

© 2025 Lima JP et al. *JAMA Network Open*.

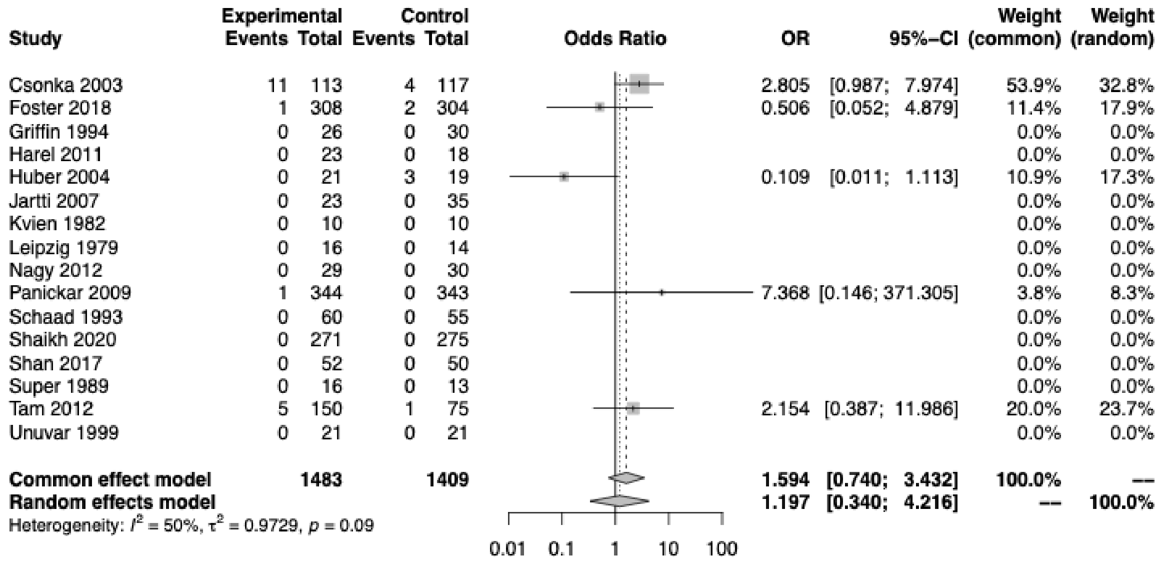

4.3 Gastrointestinal Bleeding

© 2025 Lima JP et al. *JAMA Network Open*.

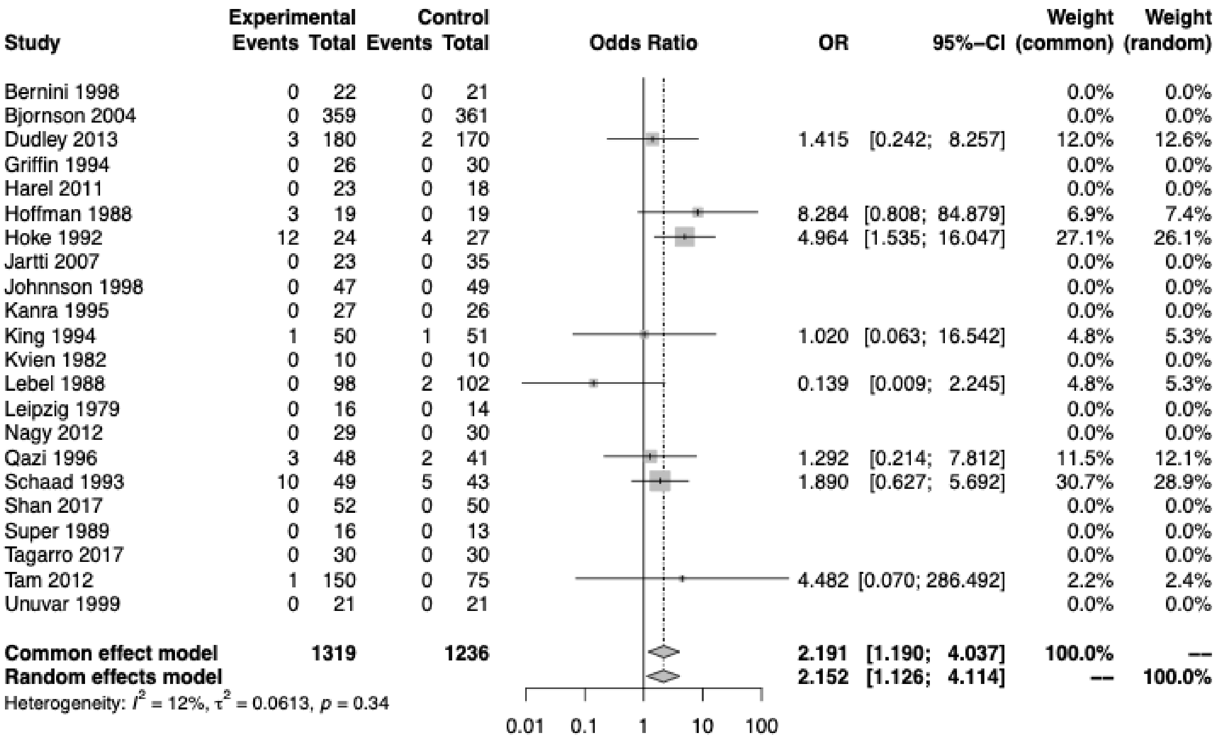

4.4 Sleep Problems

© 2025 Lima JP et al. *JAMA Network Open*.

Study	Experimental Events	Experimental Total	Control Events	Control Total	OR	95%-CI	Weight (common)	Weight (random)
Babl 2022	2	93	2	94	1.011	[0.140; 7.293]	15.5%	15.5%
Francis 2018	1	179	0	170	7.027	[0.139; 354.587]	3.9%	3.9%
Harel 2011	0	23	0	18			0.0%	0.0%
Jartti 2007	0	23	0	35			0.0%	0.0%
Kvien 1982	0	10	0	10			0.0%	0.0%
Leipzig 1979	0	16	0	14			0.0%	0.0%
Nagy 2012	0	29	0	30			0.0%	0.0%
Ranakusuma 2020	17	31	10	31	2.469	[0.912; 6.683]	61.0%	61.0%
Shaikh 2020	4	271	1	275	3.400	[0.585; 19.752]	19.5%	19.5%
Shan 2017	0	52	0	50			0.0%	0.0%
Super 1989	0	16	0	13			0.0%	0.0%
Unuvar 1999	0	21	0	21			0.0%	0.0%
**Common effect model**		**764**		**761**	**2.385**	**[1.095; 5.191]**	**100.0%**	**--**
**Random effects model**					**2.385**	**[1.095; 5.191]**	**--**	**100.0%**

Heterogeneity: $I^2 = 0\%$, $\tau^2 = 0$, $p = 0.76$

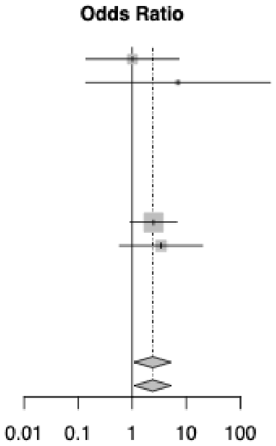

4.5 Change in Behaviour

© 2025 Lima JP et al. *JAMA Network Open*.

Study	Experimental Events	Experimental Total	Control Events	Control Total	OR	95%-CI	Weight (common)	Weight (random)
Babl 2022	6	93	3	94	2.030	[0.534; 7.718]	37.2%	37.2%
Dudley 2013	7	180	6	170	1.105	[0.365; 3.344]	54.2%	54.2%
Francis 2018	2	179	0	170	7.066	[0.440; 113.529]	8.6%	8.6%
Griffin 1994	0	26	0	30			0.0%	0.0%
Harel 2011	0	23	0	18			0.0%	0.0%
Jartti 2007	0	23	0	35			0.0%	0.0%
Kvien 1982	0	10	0	10			0.0%	0.0%
Leipzig 1979	0	16	0	14			0.0%	0.0%
Nagy 2012	0	29	0	30			0.0%	0.0%
Olympia 2005	0	57	0	68			0.0%	0.0%
Shan 2017	0	52	0	50			0.0%	0.0%
Super 1989	0	16	0	13			0.0%	0.0%
Unuvar 1999	0	21	0	21			0.0%	0.0%
**Common effect model**		**725**		**723**	**1.626**	**[0.720; 3.672]**	**100.0%**	**--**
**Random effects model**					**1.626**	**[0.720; 3.672]**	**--**	**100.0%**

Heterogeneity: $I^2 = 0\%$, $\tau^2 = 0$, $p = 0.44$

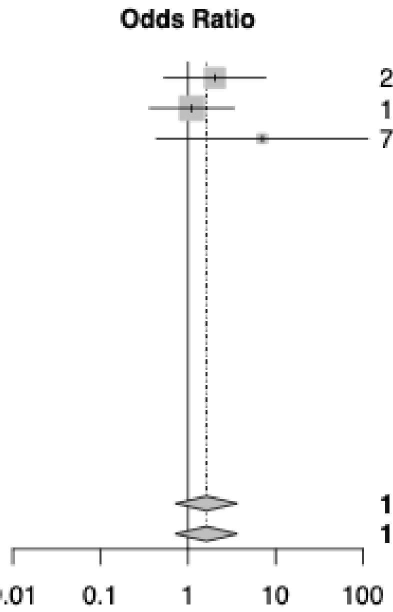

© 2025 Lima JP et al. *JAMA Network Open*.

4.6 Hyperglycemia

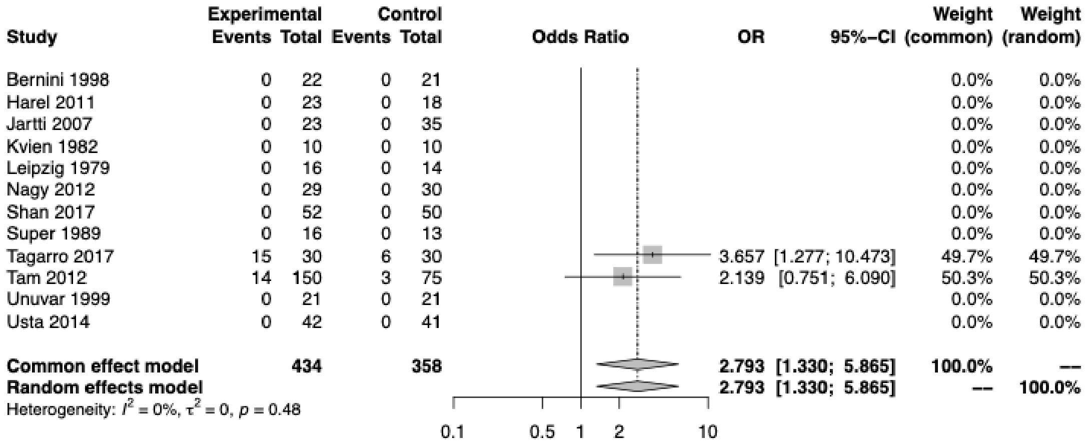

© 2025 Lima JP et al. *JAMA Network Open*.

eTable3. Previous Systematic Reviews

Author, Year	Main findings	Limitations
Aljebab, 2016	Among the most frequent AEs with short-course oral corticosteroids in children were vomiting, behavioral changes and sleep disturbance were among the most frequent AEs.<br>Increase in infection rates	Limited to oral interventions,<br>Exclusion of studies at high risk of bias<br>Fails to assess the certainty of evidence<br>Included observational studies and did not perform meta-analyses<br>Inclusion of observational studies<br>No meta-analyses performed
Fernandes, 2019	Among young children (<6 years) with respiratory conditions using inhaled or systemic corticosteroids (<15 days), there was little to difference on the risk of secondary infections.	Fails to assess the certainty of evidence<br>Limited number of clinical conditions<br>Inclusion of observational studies
Chaudhuri, 2024	In children and adults with sepsis, acute respiratory distress syndrome and community-accquired pneumonia, corticosteroids are associated with hyperglycemia and hypernatremia with no effect on gastrointestinal bleeding or secondary infections.	Limited number of clinical conditions
Kulkarni, 2022	In adults using systemic corticosteroid therapy, is associated with increased risk of metabolic adverse events (e.g., hyperglycemia, hypertension, weight gain and hyperlipidaemia)	Limited number of adverse events studied<br>Fails to assess the certainty of evidence
Efraij, 2018	The use of oral corticosteroids in adults with asthma is associated with complications such as diabetes, hypertension and bone and muscle complications.	Limited number of clinical conditions<br>Fails to assess the certainty of evidence

© 2025 Lima JP et al. *JAMA Network Open*.

2 **eTable4. Method used to capture adverse events in included studies**

Author, Year	Method
Babl 2022	Fourteen days after randomization, the parent/guardian/ participant received a phone call from the study team to assess AEs. One month after randomization, participants attended a visit at the study site where they had been recruited. If the participant was unable to attend the study site, this was completed through videoconferencing. A specialist clinician reviewed the participant for AEs.
Bernini 1998	NR
Carcao 2020	Follow-up AE questionnaires were completed by the parents/children in clinic or by telephone by local study coordinators.
Connett 1994	NR
Csonka 2003	Diary card recordings were made twice daily for 14 days. Children were examined by the study physician 14 to 21 days after the initial ED visit. Diary entries and patient files were reviewed, and patients who did not return for reassessment were contacted by telephone.
Dudley 2013	AEs assessed by clinician ar the end of the 4-week visit.
Foster 2018	AEs were captured at follow-up via completion of a 7-day symptom diary and at a 10-day and 3-month phone call by masked research assistants. During follow-up phone calls, legal guardians were asked for the presence of vomiting or any other reasons for not completing the study drug.

© 2025 Lima JP et al. *JAMA Network Open*.

Francis 2018	Legal guardians were provided with a symptom diary to complete at home during the first 5 weeks used to record AEs.
Griffin 1994	NR
Harel 2011	NR
Hoffman 1988	NR
Hoke 1992	NR
Huber 2004	Structured diary was completed by legal guardians to document AEs during 14 days.
Jartti 2007	NR
Johnnson 1998	NR
Kanra 1995	NR
King 1994	NR
Kuusela 1988	NR
Kvien 1982	NR
Lebel 1988	NR
Leipzig 1979	NR
Lv 2022	NR
Medeiros 2007	NR
Nagy 2012	NR

© 2025 Lima JP et al. *JAMA Network Open*.

Newburger 2007	NR
Ogata 2012	AEs were documented in the daily medical record reviews by nurses.
Olympia 2005	The child or child’s legal guardian was contacted by telephone daily by the research team from the time of discharge to the time of complete resolution of the sore throat.
Panickar 2009	NR
Pierson 1974	NR
Prakash 2006	NR
Qazi 1996	The presence of AEs, including death,and their possible relation to the administration of the study drugs was assessed daily. These events included secondary fever (defined as recurrence of temperatures higher than 37.8°C after 24hours or more and evidence of blood testing at least three times per week) gastrointestinal bleeding assessed by occult.
Ranakusuma 2020	NR
Ruohola 1999	NR
Schaad 1993	NR
Shaikh 2020	Staff reviewed the child's medical record for any medical care visits since their last study contact for any AEs. If child was seen or had any new symptoms within 2 weeks of taking study product, the symptoms were reported as AEs. After 2 weeks of taking study product, if a child was seen for fever or urinary symptoms but not diagnosed with a urinary tract infection these symptoms were reported as an AE.

© 2025 Lima JP et al. *JAMA Network Open*.

Shan 2017	NR
Sundel 2003	Safety of treatment was assessed by the occurrence of prospectively defined aAEs that were documented during daily medical record reviews by the study nurse.
Super 1989	NR
Tagarro 2017	NR
Tam 2012	NR
Tassniyom 1993	NR
Unuvar 1999	NR
Usta 2014	Patients in the study group were monitored for the AEs of the treatment including hyperglycemia, high blood pressure, electrolyte disturbances, and infections.
Wang 2022	NR

NR: Not reported

© 2025 Lima JP et al. *JAMA Network Open*.
