# Supplementary material for: Adverse Events Following Short-Course Systemic Corticosteroids Among Children and Adolescents: A Systematic Review and Meta-Analysis
Source: JAMA Netw Open. 2025 Sep 30;8(9):e2534953. doi: 10.1001/jamanetworkopen.2025.34953 (PMC12485646; doi:10.1001/jamanetworkopen.2025.34953)
Supplement: Supplement 2. — Data Sharing Statement [file jamanetwopen-e2534953-s002.pdf]

Data Sharing Statement

Lima. Adverse Events Following Short-Course Systemic Corticosteroids Among Children and Adolescents. *JAMA Netw Open*. Published October 01, 2025.
doi:10.1001/jamanetworkopen.2025.34953

Data
Data available: No

Additional Information
Explanation for why data not available: Data and statistical code will be made available from the corresponding author upon reasonable request.
